# A genome-wide CRISPR screen identifies interactors of the autophagy pathway as conserved coronavirus targets

**DOI:** 10.1371/journal.pbio.3001490

**Published:** 2021-12-28

**Authors:** Annika Kratzel, Jenna N. Kelly, Philip V’kovski, Jasmine Portmann, Yannick Brüggemann, Daniel Todt, Nadine Ebert, Neeta Shrestha, Philippe Plattet, Claudia A. Staab-Weijnitz, Albrecht von Brunn, Eike Steinmann, Ronald Dijkman, Gert Zimmer, Stephanie Pfaender, Volker Thiel

**Affiliations:** 1 Institute of Virology and Immunology, Bern and Mittelhäusern, Switzerland; 2 Department of Infectious Diseases and Pathobiology, Vetsuisse Faculty, University of Bern, Bern, Switzerland; 3 Graduate School for Biomedical Science, University of Bern, Bern, Switzerland; 4 European Virus Bioinformatics Center, Jena, Germany; 5 Department for Molecular & Medical Virology, Ruhr-Universität Bochum, Germany; 6 Division of Neurological Sciences, Vetsuisse Faculty, University of Bern, Bern, Switzerland; 7 Comprehensive Pneumology Center, Institute of Lung Biology and Disease, Helmholtz-Center Munich, Member of the German Center of Lung Research (DZL), Munich, Germany; 8 Max von Pettenkofer-Institute, Ludwig-Maximilians-Universität München, Munich, Germany; 9 German Center for Infection Research, Munich Site, Munich, Germany; 10 Institute for Infectious Diseases, University of Bern, Bern, Switzerland; New York University School of Medicine, UNITED STATES

## Abstract

Over the past 20 years, 3 highly pathogenic human coronaviruses (HCoVs) have emerged—Severe Acute Respiratory Syndrome Coronavirus (SARS-CoV), Middle East Respiratory Syndrome Coronavirus (MERS-CoV), and, most recently, Severe Acute Respiratory Syndrome Coronavirus 2 (SARS-CoV-2)—demonstrating that coronaviruses (CoVs) pose a serious threat to human health and highlighting the importance of developing effective therapies against them. Similar to other viruses, CoVs are dependent on host factors for their survival and replication. We hypothesized that evolutionarily distinct CoVs may exploit similar host factors and pathways to support their replication cycles. Herein, we conducted 2 independent genome-wide CRISPR/Cas-9 knockout (KO) screens to identify MERS-CoV and HCoV-229E host dependency factors (HDFs) required for HCoV replication in the human Huh7 cell line. Top scoring genes were further validated and assessed in the context of MERS-CoV and HCoV-229E infection as well as SARS-CoV and SARS-CoV-2 infection. Strikingly, we found that several autophagy-related genes, including TMEM41B, MINAR1, and the immunophilin FKBP8, were common host factors required for pan-CoV replication. Importantly, inhibition of the immunophilin protein family with the compounds cyclosporine A, and the nonimmunosuppressive derivative alisporivir, resulted in dose-dependent inhibition of CoV replication in primary human nasal epithelial cell cultures, which recapitulate the natural site of virus replication. Overall, we identified host factors that are crucial for CoV replication and demonstrated that these factors constitute potential targets for therapeutic intervention by clinically approved drugs.

## Abstract 2

This study identifies essential host dependency factors for human coronavirus replication, showing that these can be directly targeted by clinically approved inhibitors and that treatment leads to effective inhibition of coronavirus replication in primary human nasal epithelial cell cultures.

## Introduction

Coronaviruses (CoVs) are positive-sense single-stranded enveloped RNA viruses with a broad host tropism and, in case of the 3 highly pathogenic zoonotic CoVs, the ability to cross species barriers and infect humans. Since 1960, 7 human coronaviruses (HCoVs) with a suspected zoonotic origin in bats, mice, or domestic animals have been identified, including 4 seasonally circulating well-established human pathogens (HCoV-229E, HCoV-OC43, HCoV-NL63, and HCoV-HKU1) that usually cause mild symptoms like the common cold and diarrhea in immunocompetent patients [1–4]. HCoV infections have therefore generally been considered harmless; however, the relatively recent emergence of 3 highly pathogenic HCoVs, which infect the upper and also lower respiratory tract and cause severe disease in humans, has demonstrated that HCoVs can deleteriously impact human health. Between 2002 and 2003, the highly pathogenic Severe Acute Respiratory Syndrome Coronavirus (SARS-CoV) was responsible for an outbreak of severe viral pneumonia causing disease in over 8,000 patients [5]. Moreover, the emergence of Middle East Respiratory Syndrome Coronavirus (MERS-CoV) in 2012 marked the second occurrence of a highly pathogenic CoV in humans and has persistently caused endemics in the Middle East via zoonotic transmissions from dromedary camels and nosocomial outbreaks [6–8]. The newly emerged Severe Acute Respiratory Syndrome Coronavirus 2 (SARS-CoV-2), the causative agent of Coronavirus Disease 2019 (COVID-19), continues to create an imminent threat to global health, with almost 250 Mio individuals currently infected in >200 countries and more than 5 Million (Mio) fatalities (November 6, 2021) (Johns Hopkins University and Medicine Coronavirus Resource Center).

The lack of specific pharmaceutical intervention options and/or prevention measures against HCoVs, as well as ongoing difficulties containing the rapid global spread of SARS-CoV-2, has intensified in the current pandemic, and new therapies are urgently needed. CoVs are obligate intracellular pathogens and thus rely on selected host proteins, termed host dependency factors (HDFs), to achieve virus entry, replication, and release. The identification of HDFs is therefore crucial for understanding essential host–virus interactions required for successful viral replication and can provide a framework to guide the development of new pharmacological strategies for the treatment of CoV infection, including for COVID-19 and future emerging CoVs. CoVs encode a spike surface glycoprotein, which enables specific binding to a cellular receptor to mediate viral entry into the host cell. Known host receptors include dipeptidyl peptidase 4 (DPP4) for MERS-CoV, human aminopeptidase N (ANPEP/APN) for HCoV-229E, and angiotensin converting enzyme 2 (ACE2) for SARS-CoV and SARS-CoV-2 [9–12]. Cleavage of the spike protein by host cell proteases, such as TMPRSS2, cathepsin L, and/or furin, facilitates membrane fusion followed by release of the viral genome into the cellular cytoplasm for replication [13]. One hallmark that occurs in host cells during replication of positive-stranded RNA viruses is the extensive remodeling of host endomembranes that results in the formation of double-membrane vesicles (DMVs) and convoluted membranes (CMs) to which the viral replication and transcription complexes are targeted [14–16]_._ Notably, the host factors required for the formation of these structures remain elusive. Newly synthesized viral RNA is assembled to viral particles at the endoplasmic reticulum–Golgi intermediate compartment (ERGIC) and trafficked to the Golgi for posttranslational modifications [17]. Although little is known about how HCoVs exit from infected cells, recent work found that the β-CoVs mouse hepatitis virus (MHV) and SARS-CoV egress from cells via a lysosome-based pathway [18].

To identify key HDFs essential for CoV infection, we performed 2 independent genome-wide loss-of-function CRISPR screens with MERS-CoV, a highly pathogenic CoV, and HCoV-229E, an endemic CoV that causes mild respiratory symptoms in humans. We sought to uncover HDFs required for infection by a wide range of CoVs, including highly pathogenic CoVs with pandemic potential. Our results revealed that a number of autophagy-related genes, including FK506 binding protein 8 (FKBP8), transmembrane protein 41B (TMEM41B), vacuole membrane protein 1 (VMP1), and Membrane Integral NOTCH2 Associated Receptor 1 (MINAR1), were among the top hits in both CoV screens, suggesting that host factors involved in autophagy may also be required for CoV replication. Importantly, we found that perturbation of FKBP8 and other members of the immunophilin family by clinically approved and well-tolerated drugs, but not inhibition of late cellular autophagy, inhibited CoV infection in a dose-dependent manner. Overall, the genes and pathways identified in our CoV screens expand the current repertoire of essential HDFs known to be required for CoV replication and can be exploited to identify novel therapeutic targets for host-directed therapies against both existing and future emerging CoVs.

## Results

### Two independent genome-wide CRISPR/Cas-9 KO screens reveal CoV HDFs

We performed 2 independent genome-wide loss-of-function CRISPR screens with MERS-CoV and HCoV-229E to uncover unknown HDFs required for CoV replication. To conduct these CRISPR screens, we employed the well-established human GeCKOv2 genome-wide library, which includes 65,386 unique single guide RNAs (sgRNAs) targeting 19,052 protein-coding genes [19]. As a screening platform, we selected human hepatoma Huh7 cells for several reasons. First, Huh7 cells endogenously express DPP4 and ANPEP, the host cell receptors for MERS-CoV and HCoV-229E, respectively [9,10]. Huh7 cells are thus susceptible to infection with both viruses. Second, both MERS-CoV and HCoV-229E induce cytopathic effects in Huh7 cells following viral infection, which allows for rapid selection of CRISPR knockout (KO)–mediated nonsusceptible cells. Finally, several recent studies have also selected Huh7 cells for the CRISPR-based screening of other CoVs, including the novel, pathogenic SARS-CoV-2 [20–22].

**Fig 1:**
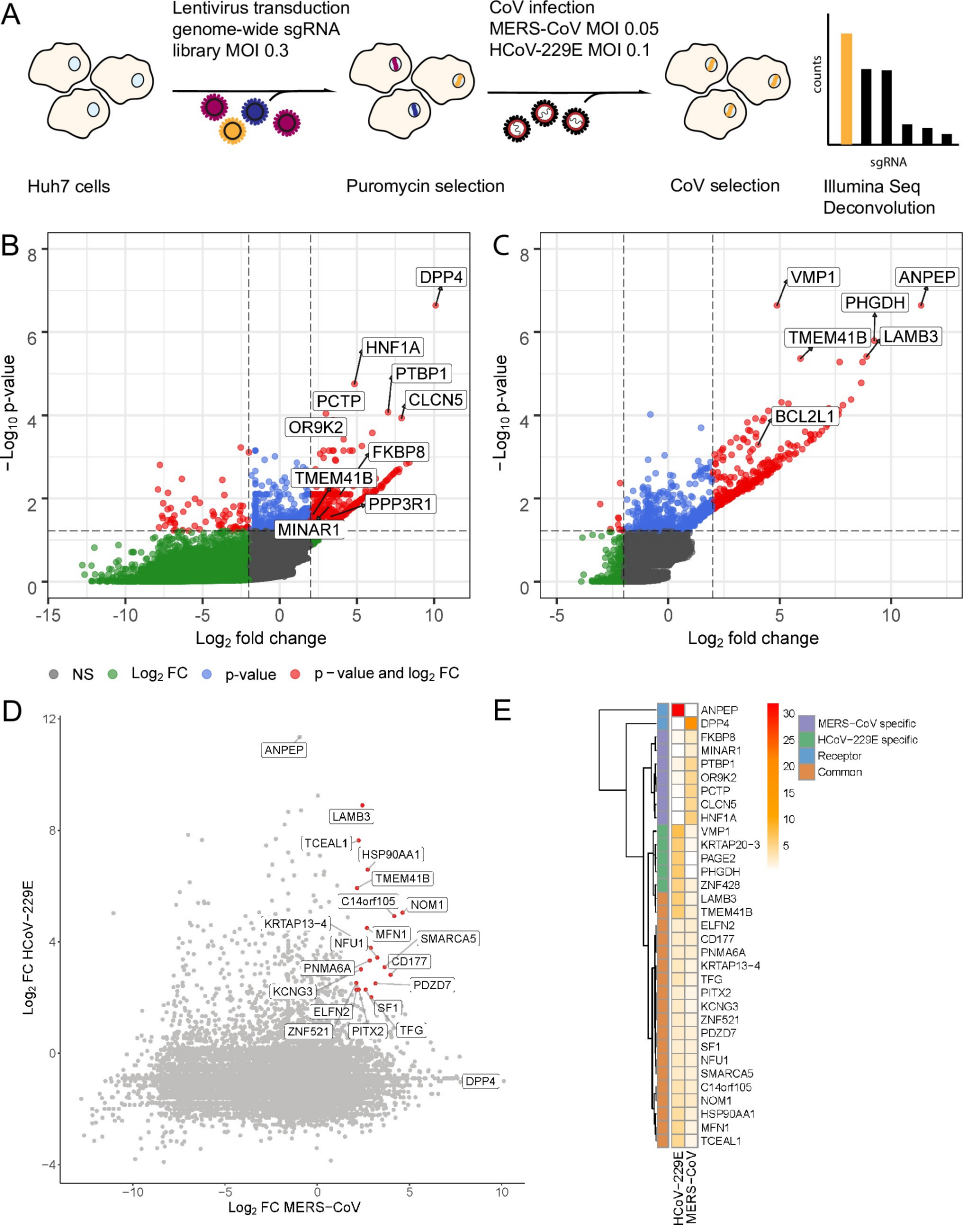
MERS-CoV and HCoV-229E genome-wide CRISPR/Cas-9–mediated KO screens. **(A)** Native Huh7 cells were transduced with the GeCKOv2 lentiviral genome-wide CRISPR library, ensuring a coverage of approximately 500 cells per sgRNA. Transduced cells were selected and then infected with either MERS-CoV or HCoV-229E at indicated MOIs and temperatures. Surviving cells were harvested and prepared for deep sequencing. Deconvolution identified both virus-specific and pan-coronavirus HDFs. **(B)** Volcano plot showing the LFC (Log_2_ FC) and log_10_ *p*-value for each gene in the MERS-CoV CRISPR screen. Genes with a FC ≥2 and *p*-value <0.05 are highlighted in red. Selected top genes are annotated in the plot, including the MERS-CoV receptor (DPP4) and the 5 most highly ranked genes in the MERS-CoV screen. **(C)** Volcano plot showing the Log_2_ FC and log_10_ *p*-value for each gene in the HCoV-229E CRISPR screen. Genes with a FC ≥ 2 and *p*-value <0.05 are highlighted in red. Selected top genes are annotated, including the HCoV-229E receptor (ANPEP) and the 5 most highly ranked genes in the HCoV-229E screen. **(D)** Pairwise comparison of enriched genes in the HCoV-229E and MERS-CoV CRISPR screens. Dotted lines indicate a Log_2_ FC ≥ 2. Genes with a Log_2_ FC ≥ 2 and *p*-value <0.05 in both screens are highlighted in red and annotated. **(E)** Heatmap comparing the log RRA *p*-values for selected top virus-specific and common hits in both CoV screens. CoV receptors (DPP4 and ANPEP) are demarcated by the blue boxes, MERS-CoV specific genes by the purple boxes, and HCoV-229E specific genes by the green boxes. Common significantly enriched genes, which are also annotated in Fig 2D, are demarcated by the orange boxes. Heatmap clustering was performed using the complete linkage method and Euclidean distance. Raw data for B–E can be found in Supporting information S1 Data, tab 1. CoV, coronavirus; DPP4, dipeptidyl peptidase 4; HCoV, human coronavirus; HDF, host dependency factor; KO, knockout; LFC, log fold change; MOI, multiplicity of infection; MERS-CoV, Middle East Respiratory Syndrome Coronavirus; RRA, robust rank aggregation; sgRNA, single guide RNA.

Genome-wide CRISPR/Cas-9 KO screens were performed by transducing Huh7 cells with the human GeCKOv2 library, selecting for library-transduced cells with puromycin, followed by infection with either MERS-CoV (37°C, multiplicity of infection [MOI] 0.05) or HCoV-229E (33°C, MOI 0.1). Surviving cells were harvested 14 days postinfection, genomic DNA was extracted, and sgRNA abundance was quantified using amplicon-based Illumina next-generation sequencing (NGS) (Fig 1A). Technical performance was evaluated using a number of quality control metrics, including an area under the curve (AUC) analysis of all sgRNAs found in samples from each screen. AUC analysis confirmed that library representation was diverse and properly maintained in uninfected samples from both screens. As expected, AUC analysis also revealed a much greater level of sgRNA guide dropout following infection with either MERS-CoV or HCoV-229E (S1A Fig). Pairwise correlation analysis showed that biological replicates from each screen clustered together and shared a high correlation coefficient (S1B Fig).

Using the MAGeCK pipeline [23], we performed paired analyses on uninfected and infected samples from each screen and computed gene-level scores using sgRNA log fold changes (LFCs) to identify KO genes that were significantly enriched in our MERS-CoV and HCoV-229E infected samples. Overall, we identified 1,149 significantly enriched genes in the MERS-CoV screen and 517 significantly enriched genes in the HCoV-229E screen using the robust rank aggregation (RRA) algorithm implemented in the MAGeCK pipeline and the “alpha median” method to calculate gene-level LFCs between samples. RRA analysis using the second-best LFC method identified 989 significantly enriched genes in the MERS-CoV screen and 332 significantly enriched genes in the HCoV-229E screen (S1 Table). To prioritize genes and generate a more robust data set, we focused on genes identified as significantly enriched by both LFC methods (i.e., RRA *p*-value ≤ 0.05 and LFC ≥ 2; S1C and S1D Fig). In total, 944 genes from the MERS-CoV screen and 332 genes from the HCoV-229E screen met these criteria, including 19 genes that were identified by both methods in both screens (Fig 1B and 1D, S1D Fig). Top scoring genes from both screens are shown in Fig 1E, including several virus-specific genes as well as the 19 aforementioned common genes. Importantly, in the MERS-CoV screen, the DPP4/CD26 host cell receptor was identified as the top scoring gene, whereas in the HCoV-229E screen, the top scoring gene was ANPEP/CD13. Moreover, the known DPP4 transcription factor HNFA1 was ranked second in the MERS-CoV screen, demonstrating the robustness of our CoV screens. We did not observe significant enrichment of the cellular proteases TMPRSS2 (*p*-value = 0.32526 and rank = 6,626 for HCoV-229E; *p*-value = 0.90798 and rank = 19,707 for MERS-CoV), TMPRSS4 (*p*-value = 0.60519 and rank = 10,916 for HCoV-229E; *p*-value = 0.60285 and rank = 12,909 for MERS-CoV), furin (*p*-value = 0.60519 and rank = 13,518 for HCoV-229E; *p*-value = 0.17977 and rank = 6,118 for MERS-CoV), or cathepsin L (*p*-value 0.29553 and rank = 6,102 for HCoV-229E and *p*-value = 0.34682 and rank = 9,302 for MERS-CoV) in our CoV screens, suggesting that despite the importance of these factors for CoV infection, they might not be essential, or their specific function may be carried out by other functionally similar proteins [24]. Indeed, one limitation of genome-wide CRISPR screens is that genes with redundant functions that may normally contribute to a particular cellular process are often missed. Interestingly, we did find that TMPRSS9 and cathepsin H were significantly enriched in our HCoV-229E and MERS-CoV screens, respectively. TMPRSS9 is highly expressed in lungs [25], and a possible role of TMPRSS9 in biological pathways leading to respiratory symptoms has been suggested [26]. Its role in HCoV infection needs to be further elucidated. Moreover, cathepsin H plays a role in surfactant processing in alveolar type II cells, a major CoV target cell type in the lung [27].

**Fig 2:**
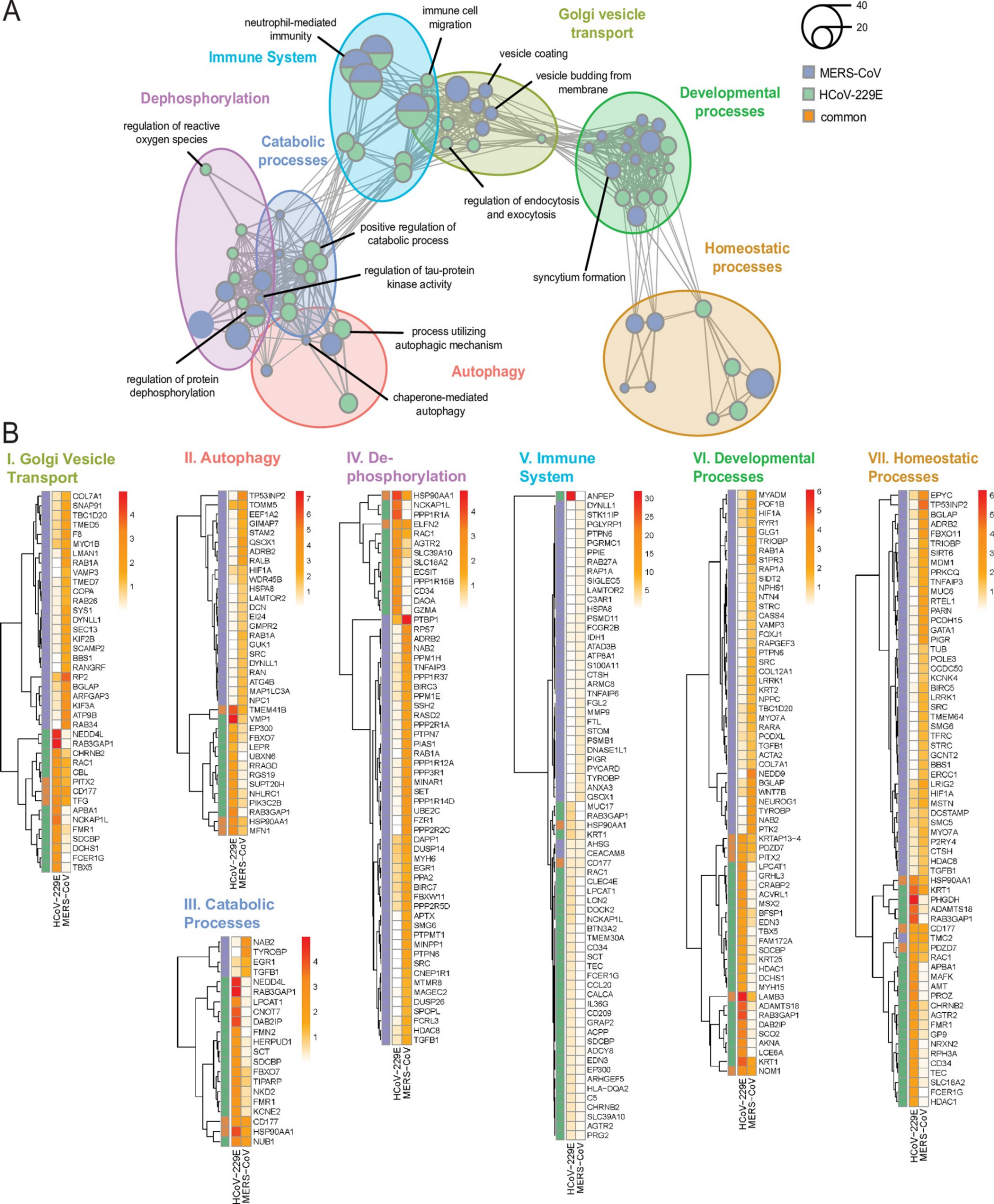
Enrichment analysis uncovers host biological networks crucial for CoV replication. **(A)** Enrichment map summarizing major host biological networks co-opted by CoVs during infection. GO enrichment analysis was performed using hits from both MERS-CoV and HCoV-229E CRISPR screens and filtered to contain conserved representative GO terms and genes. Each node represents an individual GO term and nodes that are functionally related cluster together into a larger network. Node size reflects number of significantly enriched genes in the node, and color indicates the CoV screen for which the node was significant. Raw data for (A) can be found in Supporting information S1 Data, tab 2. A complete list of significant GO terms can be found in S2 Table. **(B)** Heatmaps of individual biological clusters displayed in (A). Heatmaps contain significantly enriched genes from both CoV screens that were associated with significantly enriched GO terms found within the individual biological clusters in (A). Colored panels on the left-hand side of heatmaps show which CoV screen contained specific enriched genes (purple: MERS-CoV, green: HCoV-229E, and orange: enriched in both CoV screens). Colors in each legend represent the log RRA *p*-values for each gene in each CoV screen. Heatmap clustering was performed using the complete linkage method and Euclidean distance. Genes associated with significant GO terms are listed in Supporting information S1 Data, tab 2. Raw data for these genes can be found in Supporting information S1 Data, tab 1. CoV, coronavirus; GO, Gene Ontology; HCoV, human coronavirus; MERS-CoV, Middle East Respiratory Syndrome Coronavirus; RRA, robust rank aggregation.

To identify and compare host cell biological processes (BPs) that may be required for CoV replication, we next performed Gene Ontology (GO) enrichment analysis on each screen using the enriched genes identified above. This analysis uncovered multiple BPs that were significantly enriched in both CoV screens, many of which clustered together into 7 overarching biological themes (Fig 2A). Next, we calculated the semantic similarity among the 636 unique GO terms (BP) that were identified as significantly enriched in one or both screens (*p*-value <0.05; S2 Table). Hierarchical clustering was then used to group similar GO terms together, and a representative term for each group was selected based on scores assigned to each term. The latter analysis led to the identification of 44 conserved representative GO terms and 51 virus-specific representative GO terms (S2A Fig). Representative GO terms found in both MERS-CoV and HCoV-229E screens included a number of immune-related terms as well as terms related to the regulation of phosphorylation, kinase activity, autophagy, and lipid transport. Several specific GO terms were also significantly enriched in both screens, including neutrophil-mediated immunity, regulation of protein dephosphorylation, and regulation of the c-Jun N-terminal kinase (JNK) cascade (S2B Fig). GO terms specific to our MERS-CoV screen included regulation of exit from mitosis, protein glycosylation, and syncytium formation via plasma membrane fusion. By contrast, GO terms specific to HCoV-229E included regulation of coagulation and nitric oxide biosynthesis (S2A Fig).

To establish which pathways and/or processes may be particularly important for CoV replication, we next focused on conserved representative GO terms that included 1 or more of the 19 genes that were significantly enriched in both of our CoV screens (Fig 1D and 1E). The resulting 70 unique GO terms and their relationships to each other are the terms illustrated in Fig 2A. The 7 prominent biological themes these 70 terms clustered into are also shown and include autophagy, immunity, dephosphorylation, Golgi vesicle transport, catabolic processes, homeostatic processes, and developmental processes. To examine each biological cluster in more detail, we constructed cluster-specific heatmaps showing all enriched genes from both CoV screens associated with that cluster (Fig 2B). Furthermore, for each cluster, we inspected the network of functionally related GO terms that comprise the cluster (S3–S9 Figs). Overall, our results indicate the involvement of diverse BPs in both MERS-CoV and HCoV-229E replication cycle.

### Components of the autophagy pathway are involved in CoV infection

**Fig 3:**
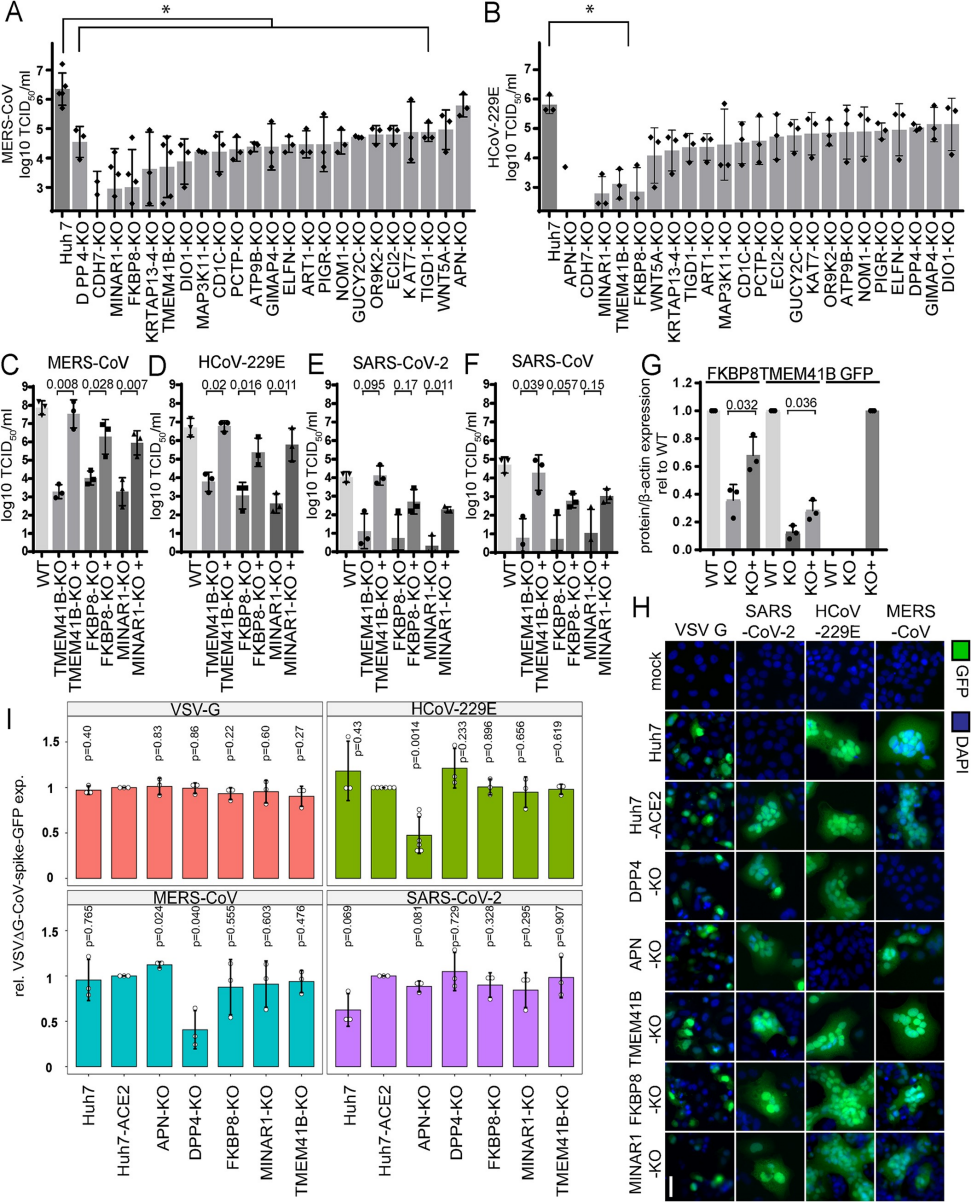
Top scoring HDFs are components of the autophagy pathway. MERS-CoV **(A)** and HCoV-229E **(B)** titers upon KO of top scoring HDFs are displayed in Log_10_ TCID_50_/ml. Raw data can be found in Supporting information S1 Data, tabs 6 and 7. MERS-CoV **(C)**, HCoV-229E **(D)**, SARS-CoV **(E)**, and SARS-CoV-2 **(F)** titers upon reconstitution of TMEM41B, FKBP8, and MINAR1 in respective KO cell lines. Titers are shown relative to WT cell line. Results are displayed as a mean of 3 independent experiments with SD represented by error bars. Raw data can be found in Supporting information S1 Data, tab 8. **(G)** Quantification of western blot analysis of FKBP8 and TMEM41B in Huh7 WT, KO, and reconstituted cells relative to beta actin. Quantification of MINAR-GFP utilizing an anti-GFP antibody in WT, KO, and MINAR1-GFP reconstituted cells relative to beta actin. Raw western blot images can be found in Supporting information S1 Raw Images. Quantification data can be found in Supporting information S1 Data, tab 9. **(H)** Immunofluorescence analysis of VSVΔG-CoV-spike-GFP and VSVΔG complemented with G in respective CTRL and KO cells. Scale bar is 40 μm. GFP is depicted in green, and DAPI is shown in blue. A total of 20 to 26 images per condition in 3 independent experiments were acquired using an Evos FL Auto 2 imaging system with a 10× air objective, analyzed and quantified in Fiji. Representative images of 1 out of 3 independent replications are shown. **(I)** Quantification of CoV-mediated entry visualized in (H). Raw data can be found in Supporting information S1 Data, tab 10. In A and B, statistical analysis was determined by ordinary 1-way ANOVA, Dunnett multiple comparison test, using Nev 2020 version 9.0. In C–G, statistical significance was determined in R (version 4.0.2) using a 2-tailed paired *t* test to compare titers between each KO and KO+ (reconstituted) cell line. A 2-tailed unpaired *t* test was performed in I, but in this case, each KO was compared to the Huh7-ACE2 control cells. Used reagents are listed in detail in Table 1. CoV, coronavirus; FKBP8, FK506 binding protein 8; HCoV, human coronavirus; HDF, host dependency factor; KO, knockout; MERS-CoV, Middle East Respiratory Syndrome Coronavirus; MINAR1, Membrane Integral NOTCH2 Associated Receptor 1; SARS-CoV, Severe Acute Respiratory Syndrome Coronavirus; SARS-CoV-2, Severe Acute Respiratory Syndrome Coronavirus 2; TCID_50_, 50% tissue culture infectious dose; TMEM41B, transmembrane protein 41B; VSV, vesicular stomatitis virus; WT, wild-type.

Based on our initial gene enrichment results from the MERS-CoV and HCoV-229E screens, as well as a comparison of the respective results with previously published data [28–30], we selected 21 hits for further experimental validation. Focusing on the highly pathogenic MERS-CoV screen, but also with an interest in examining common hits between both screens, we chose 17 genes that were significantly enriched in the MERS-CoV screen and 4 genes (TMEM41B, ELFN2, NOM1, and KRTAP13-4) that were significantly enriched in both MERS-CoV and HCoV-229E screens. For these 21 hits, stable CRISPR/Cas-9 KO cell lines were generated for each gene and then challenged with either HCoV-229E or MERS-CoV. Specific KO of the MERS-CoV receptor DPP4 and the HCoV-229E receptor APN served as controls. MERS-CoV replication could be significantly reduced in all KO cell lines, except for WNT5A and APN, thus confirming our screen and validating our data analysis (Fig 3A, S10A Fig). In contrast to MERS-CoV, HCoV-229E replication was significantly impaired upon deletion of APN as well as CDH7, MINAR1, TMEM41B, and FKBP8. Interestingly, KO of WNT5A significantly reduced HCoV-229E titers (Fig 3B, S10D Fig). Importantly, TMEM41B, FKBP8, and MINAR1 KO resulted in impaired titers for both MERS-CoV and HCoV-299E. Strikingly, SARS-CoV and SARS-CoV-2 also replicated to lower titers in respective KO cell lines expressing the specific entry receptor ACE2, confirming a conserved function in the CoV replication cycle for these 3 genes (Fig 3E and 3F, S10D Fig). To further validate the effect of the CRISPR/Cas-9–mediated KO of all the 3 host factors, we expressed CRISPR-resistant variants of these host factors and observed a rescue of virus titers for MERS-CoV, HCoV-229E, SARS-CoV, and SARS-CoV-2, thereby confirming the pan-CoV antiviral effect of TMEM41B, FKBP8, and MINAR1 KO (Fig 3C–3F). Western blot analysis confirmed stable KO of both FKBP8 and TMEM41B (Fig 3G), as well as rescue of protein expression in the transfected condition. As MINAR1 antibodies are not available, reconstituted protein expression is shown using a GFP antibody, detecting the sgRNA-resistant MINAR1-GFP fusion protein. Moreover, CRISPR/Cas-9–mediated genome editing in MINAR1, FKBP8, and TMEM41B KO cell lines were confirmed via Sanger sequencing (S10F Fig). To investigate the step of the viral replication cycle for which these factors are required, we employed a recombinant vesicular stomatitis virus (VSV) infection system, which is depleted in its surface protein G and instead stably expresses the spike protein from HCoV-229E, MERS-CoV, or SARS-CoV-2. This system specifically enables the analysis of CoV spike-mediated entry independent of CoV replication [31,32]. We found that the KO of TMEM41B, FKBP8, or MINAR1 did not alter CoV spike-mediated entry, whereas MERS-CoV spike-mediated entry was significantly reduced in DPP4-KO cells and HCoV-229E spike-mediated entry, as well as SARS-CoV-2 spike-mediated entry, was significantly impaired in APN-KO and ACE2-deficient Huh7 cells, respectively (Fig 3I and 3H). Collectively, these findings show that there is a conserved requirement for the host factors TMEM41B, FKBP8, and MINAR1 during CoV replication, but not during CoV entry.

**Fig 4:**
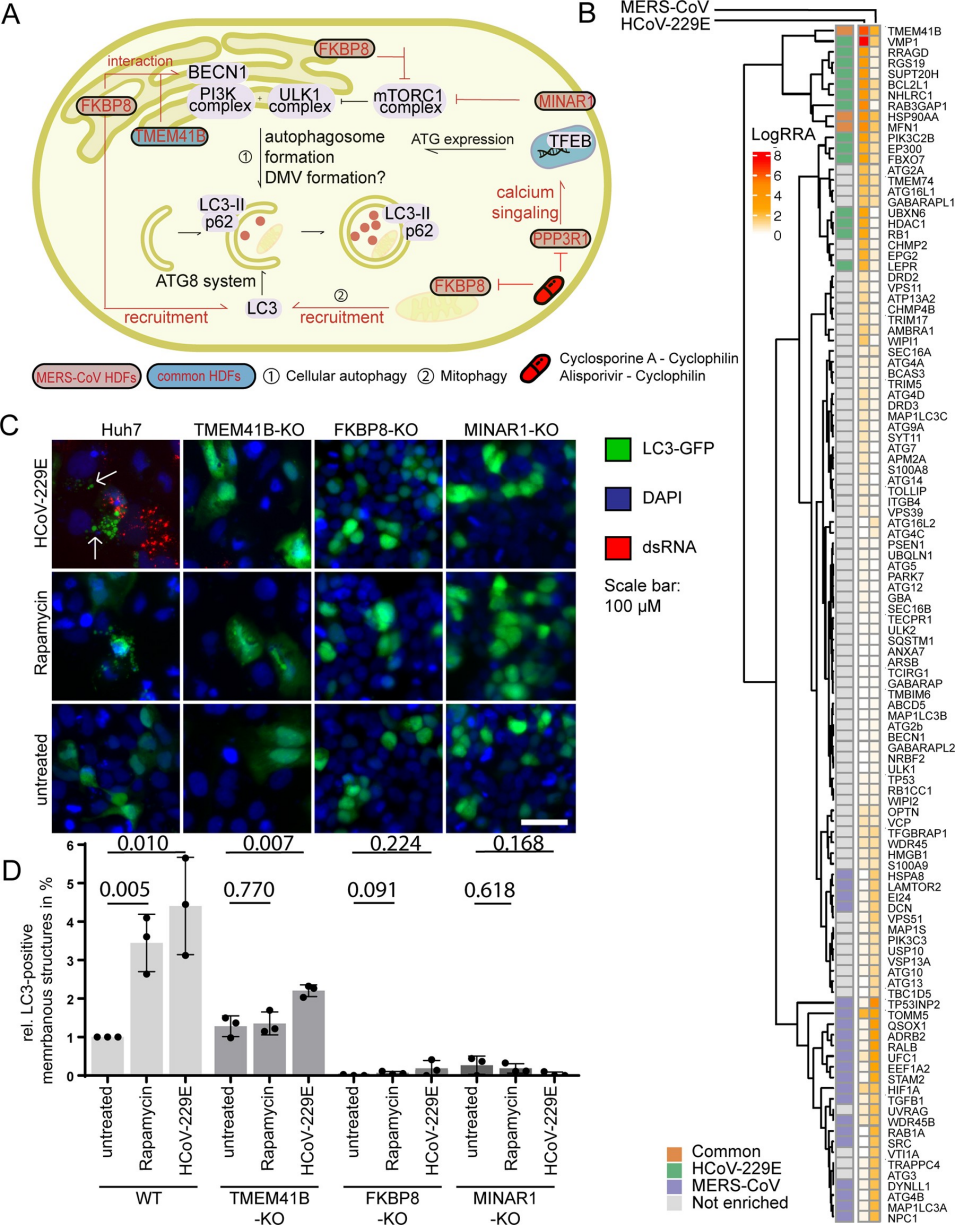
LC3-GFP puncta formation is impaired in TMEM41B, FBKP8, and MINAR1-KO cells. **(A)** Upon starvation, the mTORC1 complex is blocked and activation of the PI3K complex, as well as the ULK1 complex, leads to the initiation of phagophore formation, as an initial step in the autophagy pathway. MERS-CoV and HCoV-229E top scoring CRISPR KO screen hits FKBP8, MINAR1, TMEM41B, and VMP1 are involved in this early pathway. Furthermore, the ATG8 system containing among others LC3, which is recruited by VPM1 or FBKP8, is necessary for targeting cellular cargo to the autophagosome. PPP3R1 is up-regulated and initiates TFEB translocalization to the nucleus, where it catalyzes transcription of ATGs. MERS-CoV or conserved HDFs are indicated in respective colors. Inhibitor intervention in this pathway is shown in red. **(B)** Heatmap of canonical autophagy genes showing how host factors, including Beclin-1, ATG5, ATG7, and ATG14 are regulated in this CRISPR KO screen. MERS-CoV–specific host factors are shown in purple, HCoV-229E–specific host factors are indicated in green, and common host factors are marked in orange. Raw data for (B) can be found in Supporting information S1 Data, tab 1. **(C)** Immunofluorescence staining of LC3-GFP expressing Huh7, TMEM41B-KO, FKBP8-KO, and MINAR1-KO cells upon rapamycin treatment and HCoV-229E infection. LC3-GFP is depicted in green, dsRNA is shown in red and DAPI in blue, and scale bar is 20 μm. Representative images of 1 out of 4 independent replications are shown. Images were acquired using an EVOS FL Auto 2 imaging system with a 20× air objective and processed using Fiji. **(D)** Quantification of (C) shows LC3 puncta-positive cells upon rapamycin treatment and HCoV-229E infection in native Huh7, as well as TMEM41B-KO, FKBP8-KO, and MINAR1-KO cells. LC3-postive puncta are indicated by arrows in HCoV-229E–infected WT cells. Five images per condition in 3 independent experiments were acquired using an Evos FL Auto 2 imaging system with a 4× air objective, analyzed and quantified in Fiji. Each cell that depicted LC3-positive puncta was counted as a puncta positive cell, independent of the actual number and size of puncta. Statistical significance determined using the Holm–Sidak method, with alpha = 0.05 in GraphPad Prism 8.3.1. Each row was analyzed individually, without assuming a consistent SD. Raw data can be found in Supporting information S1 Data, tab 11. Used reagents are listed in detail in Table 1. FKBP8, FK506 binding protein 8; HCoV, human coronavirus; HDF, host dependency factor; KO, knockout; MERS-CoV, Middle East Respiratory Syndrome Coronavirus; MINAR1, Membrane Integral NOTCH2 Associated Receptor 1; TMEM41B, transmembrane protein 41B; VMP1, vacuole membrane protein 1; WT, wild-type.

Despite having distinct cellular functions, TMEM41B, FKBP8, and MINAR1 are all involved in the cellular or mitochondrial autophagy pathways, albeit at different stages (Fig 4A) [28–30,33,34]. As autophagy was also identified as one of the main conserved biological clusters in our GO analysis, we next chose to focus on these factors in the context of autophagy for further analysis. To confirm the association of TMEM41B, FKBP8, and MINAR1 with cellular autophagy, we induced autophagy in LC3-GFP transfected KO cells using rapamycin and subsequently infected these cells with HCoV-229E. Under normal physiological conditions, the cytosolic protein LC3 translocates to autophagosomal membrane structures during early autophagy, illustrated in IF via LC3-GFP-positive puncta [35]. We thus analyzed the ability of LC3-GFP to translocate to such vesicles in TMEM41B-, FKBP8-, and MINAR1-KO cells infected with HCoV-229E and undergoing autophagy as described previously [35] and analyzed our results using immunofluorescence (Fig 4C and 4D). In line with previous reports, we confirmed by visualizing LC3-GFP-positive puncta formation that rapamycin treatment induced specific vesicle formation in native Huh7 cells, but not in TMEM41B-KO, FKBP8-KO, or MINAR1 KO cells, reasserting the necessity of these proteins for autophagosome formation. Similarly, LC3-GFP-positive puncta (indicated by white arrows) accumulated in Huh7 cells during HCoV-229E infection, but less so in TMEM41B-KO, FKBP8-KO, and MINAR1-KO cells (Fig 4C and 4D). To further understand the correlation between TMEM41B, FKBP8, and MINAR1 function during HCoV-229E infection and autophagy, we analyzed the protein levels of the autophagy marker LC3II in native, rapamycin-treated, and HCoV-229E–infected KO cells (S10C Fig). The accumulation of baseline LC3II in TMEM41B-, FKBP8-, and MINAR1-KO cells confirms a role for all 3 factors during autophagy. However, LC3II levels during HCoV-229E infection and rapamycin treatment of WT and TMEM41-, MINAR1-, and FBKP8-KO cells are not changed, except for a significant accumulation of LC3II in MINAR1-KO cells after rapamycin treatment (S10B and S10C Fig). Moreover, protein levels of the autophagy cargo receptor p62 were analyzed in FKBP8-KO cells. Whereas both p62 expression and LC3II expression were significantly decreased in rapamycin-treated cells and reduced in HCoV-229E infected WT cells, similar treatment did not significantly alter p62 or LC3II expression levels in FKBP8-KO cells (S10B Fig). Together, these results show that KO of TMEM41B, FKBP8, and MINAR1 impairs membrane remodeling during rapamycin-induced autophagy and compromises LC3-GFP translocation during HCoV-229E infection and that rapamycin-induced autophagy as well as HCoV-229E expression interferes with LC3II and p62 in WT cells, but not in KO cells.

### Inhibition of the immunophilin protein family with preexisting drugs

TMEM41B, FKBP8, and MINAR1 have all been implicated as interactors of the autophagy pathway (Fig 4A). Moreover, FKBP8 is part of a large immunophilin family of proteins. Interestingly, in addition to FKBP8, several cyclophilins (additional members of the immunophilin family) were also significantly enriched in our MERS-CoV and HCoV-229E CRISPR KO screens, including peptidyl-prolyl isomerase (PPI) B, PPIC, PPID, PPIE, PPIF, PPIG, and PPIH. Proteins of this family specifically bind cyclosporine A, an immunosuppressant drug that is usually applied to suppress rejection after internal organ transplantation. Notably, while there are several drugs currently available for SARS-CoV-2 treatment, such as the antiviral drug remdesivir, and neutralizing monoclonal antibodies or antibody cocktails, such as LY-CoV555 and REGN-CoV2, many of these drugs have limitations, including SARS-CoV-2 specificity or use authorization only during certain stages of disease progression. For example, SARS-CoV-2 immune evasion, seen for some emerging variants of concern, render treatment with monoclonal antibodies ineffective if the antibody-specific epitopes are affected. It is thus critically important to not only conduct further research on existing antiviral therapies, but to also identify more drugs that effectively target CoV replication. Given the results from our CRISPR KO screen, we therefore tested whether the preexisting, clinically approved immunophilin targeting drugs cyclosporine A and alisporivir (a nonimmunosuppressant derivative of cyclosporine A currently used for the treatment of hepatitis C virus (HCV) [36]) as well as bafilomycin A1, which inhibits the autophagic flux by disrupting the lysosomal proton pump V-ATPase, could inhibit HCoV replication in human cells. Importantly, cyclosporine A is also known to inhibit calcineurin (PP3R1, MERS-CoV–specific HDF, Fig 4A) in its complexed form with the respective immunophilin [37].

**Fig 5:**
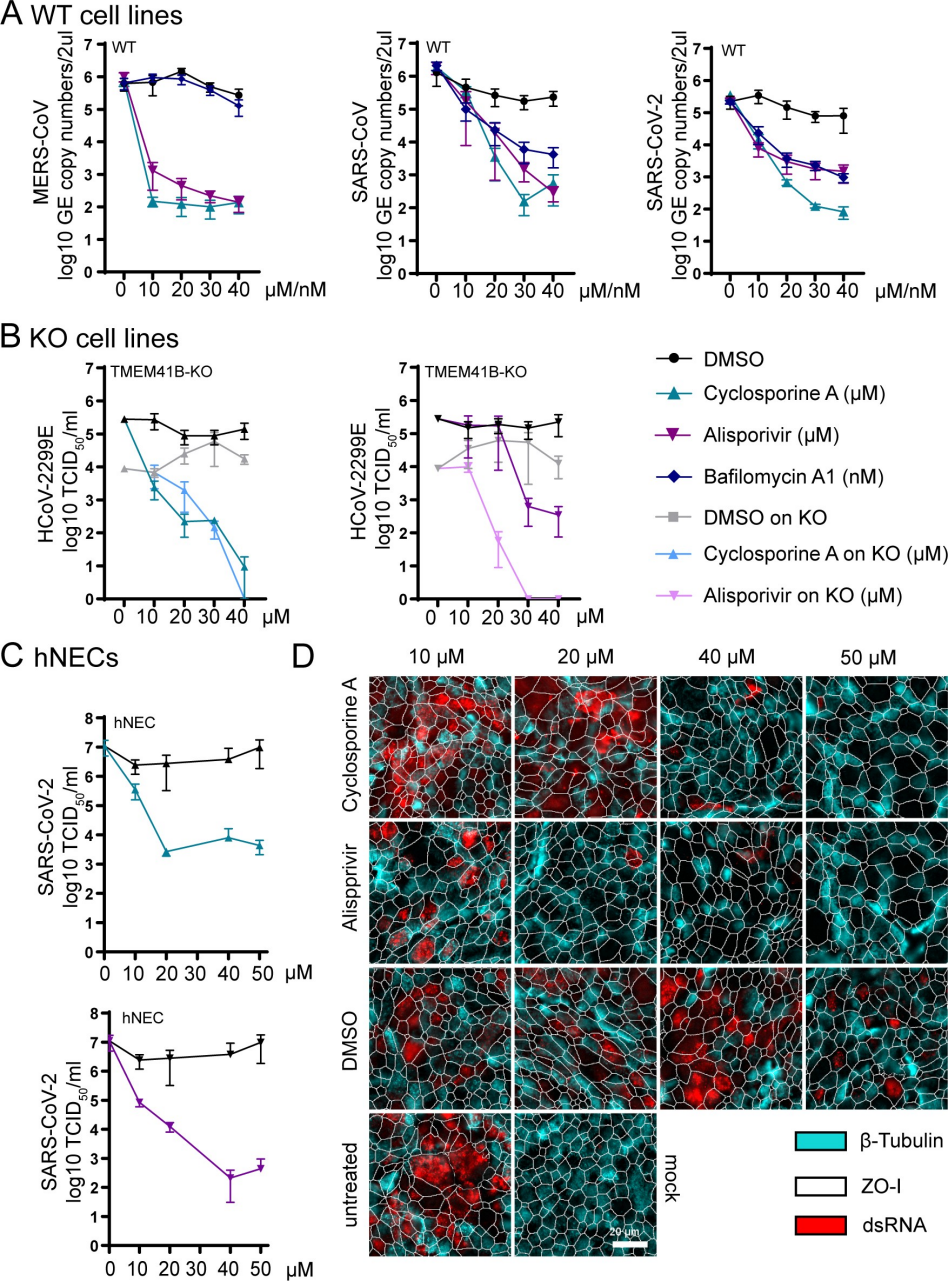
CoV HDFs are interactors of the autophagy pathway but do not depend on autophagy for replication. **(A)** Inhibitors on WT cell lines (3 graphs): MERS-CoV, SARS-CoV, and SARS-CoV-2 replication in log_10_ GE copy numbers/2 μl upon treatment of Huh7 (MERS-CoV) and VeroE6 (SARS-CoV and SARS-CoV-2) cell lines with cyclosporine A (μM), alisporivir (μM) and bafilomycin A1 (nM). GE copy numbers are shown at 24 hours postinfection/inhibitor treatment. Raw data can be found in Supporting information S1 Data, tab 12. **(B)** Inhibitors on KO cell lines (2 graphs): HCoV-229E titers in log_10_ TCID_50_/ml in cyclosporine A and alisporivir-treated TMEM41B-KO and WT cells are indicated blue and purple. Titers are shown at 24 hours postinfection/inhibitor treatment. Raw data can be found in Supporting information S1 Data, tab 13. **(C)** Inhibitors in human nasal epithelial cell (hNEC) cultures (2 graphs): SARS-CoV-2 titers in log_10_ TCID_50_/ml in primary human nasal epithelial cells at 48 hours postinfection and in presence of cylosporine A and alisporivir. Raw data can be found in Supporting information S1 Data, tab 14. **(D)** Immunofluorescence staining of SARS-CoV-2 infected human primary nasal epithelial cells, following DMSO, alisporivir, cyclosporine A, and untreated treatment, at indicated concentrations, 48 hours postinfection. dsRNA is shown in red, tight junctions (ZO-I) are shown in white, and cilia (β-tubulin) are shown in light blue. Images were acquired using an EVOS FL Auto 2 imaging system with a 40× air objective and processed using Fiji. dsRNA (red), cilia (β-tubulin, light blue), and the outline of segmented cells (ZO-I, white) of representative images are shown. Scale bar: 20 μm. Used reagents are listed in detail in Table 1. CoV, coronavirus; HDF, host dependency factor; hNEC, human nasal epithelial cell; KO, knockout; MERS-CoV, Middle East Respiratory Syndrome Coronavirus; SARS-CoV, Severe Acute Respiratory Syndrome Coronavirus; SARS-CoV-2, Severe Acute Respiratory Syndrome Coronavirus 2; TCID_50_, 50% tissue culture infectious dose; WT, wild-type.

Over the course of HCoV infection, cyclosporine A and alisporivir treatment resulted in a dose-dependent inhibition of HCoV-229E, MERS-CoV, SARS-CoV, and SARS-CoV-2 replication in cell lines 24 hours postinfection (Fig 5A, S11A–S11C Fig). Respective cytotoxicity data are depicted in S11I and S11J Fig. The most substantial decrease of genome equivalent copy numbers was up to 4 log reduction upon cyclosporine A treatment at concentrations starting at 10 μM for MERS-CoV (Fig 5A, graph 1) and 30 to 40 μM for SARS-CoV (Fig 5A, graph 2). Similar dose dependence was observed for reduction of SARS-CoV-2 replication (Fig 5A, graph 3). Interestingly, bafilomycin A1 treatment did not have an effect on MERS-CoV replication (Fig 5A, graph 1), but impaired SARS-CoV and SARS-CoV-2 replication to levels similar to the other inhibitors (Fig 5A, graph 2 and graph 3). As these immunophilin inhibitors do not specifically target TMEM41B and MINAR1, we also analyzed whether cyclosporine A and alisporivir treatment could further reduce HCoV-229E infection in TMEM41B-KO cells. We observed an additional 3 log reduction in HCoV-229E infectious titers in alisporivir-treated TMEM41B-KO cell lines (Fig 5B, graph 2). Since both inhibitors are known to inhibit a broad range of viruses [38,39], canine distemper virus (CDV), which has not been analyzed in context of these inhibitors previously, was included as a control virus to demonstrate the specificity of the compounds. Indeed, treatment with either cyclosporine A or alisporivir did not alter CDV relative infectivity at concentrations ranging from 0 to 40 uM (S11D Fig). Finally, we also analyzed the effect of treatment with cyclosporine A and alisporivir during SARS-CoV-2 infection in primary well-differentiated human nasal epithelial cell cultures, which mimic the natural site of SARS-CoV-2 infection and replication. Cyclosporine A inhibited SARS-CoV-2 replication at 48 hours postinfection by around 4 log_10_ 50% tissue culture infectious dose (TCID_50_)/ml at noncytotoxic concentrations with a half maximal inhibitory concentration (IC_50_) of 7.9 μM (Fig 5C, graph 1 and 5D, S11F and S11H Fig) and alisporivir by approximately 4 log_10_ TCID_50_/ml at noncytotoxic concentrations with an IC_50_ of 2.3 μM (Fig 5C, graph 2 and 5D, S11G and S11H Fig). Taken together, our results suggest that these immunophilin targeting drugs inhibit the function of certain CoV HDFs, thereby impairing virus replication.

## Discussion

The identification of HDFs essential for HCoV infection offers great potential to reveal novel therapeutic targets and enhance our understanding of HCoV infection and pathogenesis (e.g., COVID-19). Here, we have performed 2 independent genome-wide CRISPR/Cas-9 KO screens in Huh7 cells with HCoV-229E and MERS-CoV to identify functionally important genes during HCoV infection. Using MERS-CoV as a representative emerging virus and HCoV-229E as a representative endemic virus, we identified multiple virus-specific and conserved HDFs, including several that are required for the replication of the novel pandemic CoV SARS-CoV-2. GO enrichment analysis revealed that the conserved HDFs were involved in diverse BPs that clustered into 7 major categories. Interestingly, we found that MERS-CoV and HCoV-229E seemed to exploit different components of the same BPs, as the majority of genes involved in each biological cluster were virus specific, but the overall BPs were similar. This may be due to evolutionary differences between the viruses, as MERS-CoV is part of the betacoronavirus genus, whereas HCoV-229E is a member of alphacoronavirus genus. Furthermore, many commonly enriched genes were involved in Golgi vesicle transport, or more specifically in vesicle coating and budding from membranes, as well as regulation of endocytosis and exocytosis, which are known to be associated with virus entry and exit [40]. Moreover, Golgi vesicle markers have been found in close proximity to CoV replication compartments, suggesting another potential function for genes in this cluster during CoV replication, e.g., membrane reorganization for membranous replication compartments [41]. A second prominent category was the immune system cluster, which may be associated with direct exploitation of immunological host responses against CoVs and thus offer potential intervention strategies. These strategies may also have antiviral efficacy and work to lower dysfunctional immune responses, which is a known driver of disease progression and severe lung pathology [42]. Another major category containing enriched genes in both HCoV screens was dephosphorylation. Genes involved in phosphorylation and kinase activities were strongly enriched in our screens, suggesting that these processes are required for HCoV replication and that other CoVs also exploit the host’s phosphorylation machinery for their benefit. Importantly, recent work observed striking changes in phosphorylation on host and viral proteins during SARS-CoV-2 infection, including many changes related to dephosphorylation and altered kinase activity [43,44]. For example, the JNK signaling cascade, but also the regulation of tau-protein kinase activity, were highly enriched in our MERS-CoV screen. JNKs belong to the mitogen-activated protein kinase (MAPK) family, and SARS-CoV-2 infection was recently shown to promote p38 MAPK signaling activity [43]. Of note, the FKBP8 gene clustered into the dephosphorylation category, and the MINAR1 gene was included in regulation of tau-protein kinase activity, suggesting that these 2 genes may influence CoV replication via other BPs in addition to autophagy. Along this line, therapeutical intervention targeting AP2M1 (part of the clathrin-dependent endocytic pathway) phosphorylation using a kinase inhibitor resulted in reduced SARS-CoV, MERS-CoV, and SARS-CoV-2 infection, exemplifying the antiviral potential of targeting specific phosphorylation sites during viral infection [45]. Our analysis also found that genes involved in catabolic and homeostatic processes were significantly enriched in both CoV screens. Interestingly, a similar cluster linked to cholesterol metabolism was identified in previous studies, including SARS-CoV-2, HCoV-229E, and HCoV-OC43 genome-wide CRISPR/Cas-9–mediated KO screens and SARS-CoV-2 interactome studies [46,47] and has been linked to CoV entry and membrane fusion [48]. Furthermore, one gene that is worth mentioning, as its KO had the highest effect on MERS-CoV and HCoV-229E infectious titer reduction, is cadherin 7 (CDH7). CDH7 belongs to a family of calcium-dependent cell adhesion proteins and is expressed on the plasma membrane [49]. Interestingly, viruses such as Epstein–Barr virus (EBV), hepatitis B virus (HBV), HCV, and human herpesvirus 8 (HHV8) have previously been shown to degrade cadherins for their benefit [50]. A possible interaction between CoVs and CDH7 is therefore definitely plausible. As it is located at the plasma membrane, it might be important for HCoV entry or could possibly play a role in transmission, as has been observed for measles virus [50] and HBV [51], which use formation of adherent junctions for efficient virus transmission.

For our downstream experimental analysis, we focused on the autophagy cluster. Autophagy is a cellular stress response (e.g., to starvation or infection by pathogens) that involves the recycling of proteins and cell organelles to maintain cellular homeostasis [52]. These processes exploit a very wide-ranging group of cellular trafficking pathways required for transportation of cytoplasmic material to the lysosome for destruction. Interestingly, several autophagy-related genes were identified as top hits in our screen, including TMEM41B, FKBP8, and MINAR1. Further, we demonstrated that these host factors are required for replication of several CoVs. A similar recently published genome-wide CRISPR screen for MERS-CoV HDFs [53] identified TMEM41B as a proviral gene specific for MERS-CoV replication in African Green Monkey VeroE6-Cas-9 cells, underlining the importance of this host factor for MERS-CoV replication among different model systems. The same study identified genes involved in diverse biological pathways such as chromatin remodeling, histone modification, cellular signaling, and RNA regulation as essential for MERS-CoV replication. Another recent genome-wide CRISPR-mediated KO screen for HCoV-229E and SARS-CoV-2 host factors in Huh7 cells identified TMEM41B, as well as PIK3C3 (endocytic trafficking and autophagy), as top-ranked genes for both viruses, with a higher dependency of HCoV-229E on TMEM41B, which is in line with our results. Notably, this study also shows that disruption of the autophagy genes ATG5 and ATG7, which are required for phagophore expansion, do not block SARS-CoV-2 and HCoV-229E infection, suggesting that later stages of autophagy are not required for CoV replication [54]. These results are reflected in our study since ATG5 and ATG7 were not identified as hits in our CoV screens (Fig 4B). Finally, TMEM41B was also identified as a HCoV-229E host factor in Huh7.5 cells, along with VMP1, which ranked second in our HCoV-229E host factor screen. Interestingly, SARS-CoV-2 and HCoV-OC43, as well as HCoV-NL63, are also dependent on TMEM41B for efficient replication [20].

The ER localized TMEM41B was recently identified as a gene required for early autophagosome formation and lipid mobilization in 3 independent genome-wide CRISPR KO screens that aimed to identify host factors essential for autophagy. They also observed that TMEM41B and the well-characterized early-stage autophagy protein VMP1 (top scoring HDF in our HCoV-229E screen with *p*-value = 3.1525e-08 and rank = 2; *p*-value = 3.4752e-02 and rank = 1919 for MERS-CoV) implement related functions [28–30]. Furthermore, interaction of TMEM41B with Beclin1 (PI3K complex) underscores the importance of this protein in the induction of autophagy [55]. Interestingly, the FK506-binding protein 8 (gene: FKBP8, protein: FKBP38), a member of the immunophilin protein family, is located in the outer mitochondrial membrane and plays a key role in mitophagy by inhibiting the mTORC1 complex during nutrient deprivation [56]. Moreover, FKBP8 targets Beclin-1 to ER–mitochondria membranes during mitophagy and recruits LC3A to damaged mitochondria, thereby actively inducing the removal of excess mitochondria by autophagy [33]. FKBP8 itself avoids degradation by escaping from mitochondria and is translocated to the ER [57]. MINAR1 (also known as Ubtor or KIAA1024) was the third MERS-CoV HDF identified with a possible indirect involvement in autophagy regulation. The otherwise very rudimentarily characterized protein plays a role in regulating cell growth and mTOR signaling, as MINAR1 depletion resulted in higher mTOR activity [34] (Fig 4A). In addition, the phosphatase PPP3R1, commonly referred to as calcineurin, is up-regulated during cell starvation and controls the activity of the TFEB transcriptional regulator of lysosomal biogenesis and autophagy [58]. Importantly, the interaction between autophagy components and CoVs, but also other positive-stranded RNA viruses, during viral replication has been under discussion for a long time, as parts of the autophagy process show similarities to the process of DMV formation [41,59,60]. CoVs rely on the formation of replication complexes at DMVs, the presumed site of viral genome replication and transcription. Due to a lack of conventional ER or Golgi protein markers, the exact origin of DMVs remains unclear, and studies investigating the possible involvement of the early autophagy machinery in the conversion of host membranes into DMVs reached conflicting conclusions [61,62]. Another possibility is that single components of the autophagic machinery may be hijacked by CoVs independently of their activity in autophagic processing. The nonlipidated autophagy marker LC3 has been observed to localize to DMVs, and the down-regulation of LC3, but not inactivation of host cell autophagy, protects cells from CoV infection [59,63–65]. We show that TMEM41B, MINAR1, and FKBP8 are involved in regulating LC3-positive puncta formation following chemical induction of autophagy and HCoV-229E infection and that KO of each gene distinctly impairs HCoV replication. Further, the accumulation of LC3II protein expression in TMEM41B-, FKBP8-, and MINAR1-KO cells compared to WT cells confirmed an impaired autophagic flux in all 3 KO cells, validating these host factors as components of the autophagy pathway. When analyzing LC3II and p62 protein expression during HCoV-229E infection or rapamycin treatment in WT cells, we observed a significant decrease of both LC3II and p62 expression being in line with an up-regulated autophagic flux in the rapamycin-treated condition. As neither LC3II nor p62 protein expression was changed in the analyzed rapamycin-treated or HCoV-229E infected KO-cell lines (except for an upregulation of LC3II in rapamycin-treated MINAR1-KO cells, which is possibly still connected to a decrease in autophagic flux), further studies are required to disentangle the role of these 2 proteins during CoV infection and to clarify whether the autophagy pathway is influenced by CoV infection. Our results clearly show that with TMEM41B, FKBP8, and MINAR1, components of the autophagy pathway are involved in HCoV infection. Whether these components act via their native function which they exhibit during autophagy or whether HCoVs recruit these factors to act in an autophagy-independent way remains elusive. Further roles of the 3 identified host factors have been suggested. Both TMEM41B and FKBP8 are thought to interact with Beclin-1, which is a core subunit of the PI3K complex that drives autophagy [55,66]. Captivatingly, inhibition of SKP2, another Beclin-1 interactor, reduced MERS-CoV infection [67]. Recent work suggested a putative autophagy-independent role for TMEM41B as a pan-coronavirus and flavivirus replication factor, which is recruited to flavivirus RNA replication complexes to facilitate membrane curvature and create a protected environment for viral genome replication [68,69]. Furthermore, MINAR1 serves as a regulator of mTOR signaling, which regulates numerous cellular processes including the cap-dependent mRNA translation and synthesis machinery required during viral replication. These observations add further potential layers of modulation by TMEM41B, FKBP8, and MINAR1 during CoV replication.

Independently of the exact underlying mechanism, our results suggest that the HDFs FKBP8, TMEM41B, and MINAR1 herein represent potential targets for host-directed therapeutics. Its immunomodulating component makes FKBP8 a very interesting HDF for CoV replication. Importantly, FKBP8 has further been shown to be involved in virus replication in a completely autophagy-independent way. FKBP8 interacts with retinoic acid inducible protein 1 (RIG-I), virus-induced signaling adaptor signaling (VISA), and IFN regulatory factor 3 (IRF3) during Sendai virus infection. Knockdown of FKBP8 promotes the activation of IFN-beta and the antiviral response during Sendai virus infection in HEK293T cells, suggesting a possible immunomodulatory component for its role in CoV infection [70]. In addition to FKBP8, several cyclophilins were up-regulated in both of our HCoV screens. Cyclophilins express PPI activity, which catalyzes the isomerization of peptide bonds in proline residues from *trans* to *cis*, thereby facilitating protein folding. Proteins of this family specifically bind cyclosporine A, an immunosuppressant drug that is usually applied to suppress rejection after internal organ transplantation. Moreover, FKBPs and cyclophilins have been the focus of several CoV studies showing impaired HCoV-229E, HCoV-NL63, SARS-CoV, and MERS-CoV replication upon FKBP and cyclophilin inhibitor treatment [71–76]. Given the lack of specific treatment options during the ongoing SARS-CoV-2 pandemic, we tested cyclosporine A, as well as alisporivir, a nonimmunosuppressant derivative of cyclosporine A, and showed that antiviral intervention using these clinically approved drugs inhibited the replication of the highly pathogenic CoVs MERS-CoV, SARS-CoV, and SARS-CoV-2 in a dose-dependent manner. Moreover, inhibitor treatment on top of TMEM41B-KO could further inhibit HCoV-229E. While Huh7 and VeroE6 cells are valuable model cell lines for highly pathogenic CoVs, they likely do not capture important aspects of infection compared to primary human airway epithelial cells nor fully recapitulate the complex cellular milieu present in human patients. To address these limitations, we also tested cyclosporine A and alisporivir on primary human nasal epithelial cell cultures and found that these compounds potently inhibited SARS-CoV-2 replication at concentrations known to be achievable and efficacious in patients. Together, these findings depict a promising path toward the repurposing of cyclosporine A and alisporivir as COVID-19 treatment options. Infection with highly pathogenic CoVs is frequently accompanied by inflammatory immunopathogenesis, including the virus-induced destruction of lung tissue and subsequent triggering of a host immune response. Importantly, in certain cases, a dysregulated immune response is associated with severe lung pathology and systemic pathogenesis [42]. The latter highlights the need for dual-acting antiviral drugs that also target inflammation and/or cell death. Of interest, alisporivir also blocks mitochondrial cyclophilin-D, a key regulator of mitochondrial permeability transition pore (mPTP) opening, which is a mechanism involved in triggering cell death. Hence, besides its antiviral properties, it is possible that alisporivir also reduces CoV-induced lung tissue damage [77]. Trials using either cyclosporine A in patients with moderate COVID-19 (ClinicalTrials.gov identifier: NCT04412785 and NCT04540926) or alisporivir (ClinicalTrials.gov identifier: NCT04608214) for the treatment of hospitalized COVID-19 patients have been registered.

The identification of MINAR1, TMEM41B, and FKBP8 as conserved HCoV HDFs in our MERS-CoV and HCoV-229E screens extends our knowledge of host–virus dynamics during HCoV infection. Furthermore, the involvement of FKBP8 and other members of the cyclophilin family in HCoV replication provides more information on how cyclosporin A and alisporivir are able reduce CoV replication by interfering with essential HCoV HDFs. We confirm the potential of both inhibitors as treatment against MERS-CoV and HCoV-229E infection and additionally observed similar reduction in SARS-CoV-2 replication. Altogether, our findings highlight the potential of using genome-wide CRISPR/Cas-9 KO screens to identify novel HDFs essential for HCoV infection, which can, in turn, be used in combination with clinically available drugs to identify and evaluate host-directed therapies against existing and future pandemic CoVs.

## Materials and methods

### Resources

#### Corresponding author

Further information and request for resources and reagents should be directed to and will be fulfilled by Volker Thiel (Volker.thiel@vetuisse.unibe.ch). Unique reagents generated in this study will be made available on request.

#### Methods

Reagents mentioned in the methods part are listed in Table 1 in more detail.

**Table 1:** List of reagents used in this study including source and identifier.

Reagent	Source	Identifier
**Antibodies**		
Anti-beta-actin HRP	Sigma-Aldrich	A3854
Anti-dsRNA	SCICONS	Clone J2
Anti-FKBP8	Sigma-Aldrich	AV46863
Anti-GFP	Invitrogen	A11122
Anti-LC3B	Sigma-Aldrich	L7543
Anti-MINAR1	Sigma-Aldrich	HPA011545
Anti p62	Abcam	91526
Anti-TMEM41B	Cell Signalling Technology	#68071
Donkey-anti-mouse AlexaFluor-labeled-488 IgG (H+L)	Jackson ImmunoResearch	715-545-150
Donkey-anti-rabbit HRP	Jackson ImmunoResearch	711-035-152
Mouse-anti-ZO-1 AlexaFluor-labeled-594	Thermo Fisher Scientific	1A12
Rabbit-anti-beta tubulin IV AlexaFluor-labeled-647	Cell Signalling Technology	9F3
**Virus strains**		
HCoV-229E	In [78]	N/A
MERS-CoV	In [79]	N/A
SARS-CoV	In [80]	N/A
SARS-CoV-2	Kindly provided by Daniela Niemeyer, Marcel Müller, and Christian Drosten	SARS-CoV-2/München-1.1/2020/929
VSV-ΔG	In [31]	N/A
VSV-ΔG-HCoV-229E spike-GFP	In [31]	N/A
VSV-ΔG-MERS-CoV spike-GFP	In [31]	N/A
VSV-ΔG SARS-CoV-2 spike-GFP	Kindly provided by Sean Whelan	N/A
**Experimental models: Cell lines and primary cells**		
293-LTV	Cell Biolabs	LTV-100
BHK-G43	Kindly provided by Gert Zimmer	Previously described [81]
Huh7	Kindly provided by Volker Lohmann	N/A
VeroE6	Kindly provided by Doreen Muth, Marcel Müller, and Christian Drosten	N/A
MucilAir	Epithelix	EP01MD
**Cell culture reagents**		
DMEM	Gibco	41966029
MucilAir Medium	Epithelix	EP04MN
FBS	Gibco	A4766801
HBSS	Gibco	14065056
HEPES	Gibco	15630080
Nonessential amino acids	Gibco	11140035
OptiMEM	Gibco	31985062
Pen/Strep	Gibco	15140122
**Plasmids**		
GeCKO v2 CRISPR KO library	Feng Zhang Lab	19
LC3-GFP	In [35]	N/A
FKBP8_OHu66426C_Mutant1_pcDNA3.1(+)-C-eGFP	GenScript (customized)	N/A
MINAR1_OHu10898C_Mutant1_pcDNA3.1(+)-C-eGFP	GenScript (customized)	N/A
TMEM41B_OHu12098C_Mutant1_pcDNA3.1(+)-C-eGFP	GenScript (customized)	N/A
pSCRPSY-Tag-RFP-ACE2	Kindly provided by John Schoggins	N/A
**Chemicals and other reagents**		
Blasticidin S HCL	Gibco	R21001
Bovine Serum Albumin	Roche	10735094001
Crystal Violet	Sigma-Aldrich	61135-100G
DAPI	Thermo Fisher Scientific	D1306
DMSO	Sigma-Aldrich	D5879-1L
ECL	Advansta	K-12045-D20
Lipofectamine 2000	Thermo Fisher Scientific	11668019
M-PER	Thermo Fisher Scientific	78501
Protease Inhibitor cOmplete, Mini EDTA-free	Roche	04693159001
Saponin	Sigma-Aldrich	S4521-1QG
SDS-PAGE	GenScript	M00666
Stellar competent cells	Takara	636766
Taqman Fast Virus 1-Step	Thermo Fisher Scientific	4444434
**Inhibitors and compounds**		
Alisporivir	MedChemExpress	HY-12559
Bafilomycin A1	Sigma-Aldrich	B1793
Cyclosporine A	Sigma-Aldrich	30024
Rapamyicin	Sigma-Aldrich	S-015
**Kits**		
Bright-Glo Luciferase Assay System	Promega	E2620
CellTiterGlo 2.0 Cell viability Assay	Promega	G9241
CytoTox 96 Non-Radioactive Cytotoxicity Assay	Promega	G1780
NucleoBond Xtra Midi	Macherey Nagel	740410.50
NucleoMag Vet	Macherey Nagel	744200.4
NucleoSpin Plasmid, Mini	Macherey Nagel	740588.50
NucleoSpin Tissue	Macherey Nagel	740952.50
PowerPlex 16 HS System	Promega	DC2101
**Machines**		
Applied Biosystems 7500 Fast Dx Real-Time PCR Instrument	Life Technologies	4406985
eBlotL1	GenScript	L00686
EnSpire 2300	Perkin Elmer	N/A
Evos FL2 Auto/ M7000 Imaging System	Thermo Fisher Scientific	AMF7000
FusionFX	Vilber	N/A
KingFisher Flex Purification System	Thermo Fisher Scientific	N/A
**Software and algorithms**		
Fiji	Software	ImageJ; RRID: SCR_003070 https://imagej.nih.gov/ij/download.html
Fusion	Software	Fusion Software, Copyright 2004–2018 by Vilber Lourmat SAS
GeneMarker HID	SoftGenetics	SoftGenetics—Software PowerTools for Genetic Analysis
GraphPad Prism version 8.3.1	Software	RRID: SCR_002798 https://www.graphpad.com
MAGeCK	In [23]	https://sourceforge.net/p/mageck/wiki/Home
R 4.0.2	R	https://www.r-project.org

DMEM, Dulbecco’s Modified Eagle Medium; FBS, fetal bovine serum; HBSS, Hanks’ balanced salt solution; HCoV, human coronavirus; MERS-CoV, Middle East Respiratory Syndrome Coronavirus; SARS-CoV, Severe Acute Respiratory Syndrome Coronavirus; SARS-CoV-2, Severe Acute Respiratory Syndrome Coronavirus 2.

#### Cell lines

Human hepatoma (Huh7) cell line (kindly provided by Volker Lohmann) and African green monkey kidney (VeroE6) cell line (kindly provided by Doreen Muth, Marcel Müller, and Christian Drosten, Charité, Berlin, Germany) and 293LTV cells (purchased from Cell Biolabs, San Diego, California, USA) were propagated in Dulbecco’s Modified Eagle Medium (DMEM), supplemented with 10% heat inactivated fetal bovine serum, 1% nonessential amino acids, 100 μg/mL of streptomycin and 100 IU/mL of penicillin, and 15 mMol of HEPES. Cells were maintained at 37°C in a humidified incubator with 5% CO2. Profiling of cell lines was performed using highly-polymorphic short tandem repeat loci (STRs) and amplification using PowerPlex 16 HS System (Promega, Madison, Wisconsin, USA), followed by fragment analysison an ABI3730xl (Life Technologies, Carlsbad, California, USA) and analysis with GeneMarker HID software (SoftGenetics, State College, Pennsylvania, USA) by Mircosynth. Huh7 cell line was confirmed to be of human origin without contamination, matching the reference DNA of the cell line Huh7 (Microsynth reference, Mic_ 152021) with 96.7% and the DNA profile of Huh7 (Cellosaurus, RRID:CVCL_0336) with 90%. Moreover, 293 LTV cell line was confirmed to be of human origin without contamination, matching the reference DNA of the cell line HEK293T (ATCC CRL-3216) with 93.8% and the DNA profile of HEK293 with 86.7% (Cellosaurus, RRID:CVCL_0045). Matching at ≥80% of alleles across 8 reference loci are said to be related. VeroE6 cell line was identified to be 100% identical with *Chlorocebus sabaeus*, upon amplification and blast of mitochondrial cytochrome b gene according to Irwin and colleagues [82], using the following primers:

**Table:** 

L14724	CGAAGCTTGATATGAAAAACCATCGTTG
H15149	AACTGCAGCCCCTCAGAATGATATTTGTCCTCA

BHK-G43 cells were maintained by Gert Zimmer as previously described [81]. As cells originated from a commercial source (DSMZ collection # ACC61), profiling of cell line was not performed.

#### Primary cell culture

Primary human nasal epithelium cell cultures: MucilAir were purchased from Epithelix (Plan-les-Ouates, Switzerland). Cultures are reconstituted using human primary cells from healthy nasal region from 14 donors and cultured at an air–liquid interface in ready-to-use MucilAir Culture Medium purchased from Epithelix is serum free, contains phenol red, and is supplemented with penicillin/streptomycin. The apical side was washed with Hanks’ balanced salt solution (HBSS) prior to infection. The anonymity of the donors prevents from the determination of the cells’ sex.

### Method details

#### Genome-wide CRISPR/Cas-9–mediated KO screens

The vector lentiviral human GeCKOv2 library A [83], containing 3 sgRNAs per gene, was transfected into 293 LTV cells for lentivirus production using Lipofectamine 2000 (Thermo Fisher Scientific, Darmstadt, Germany). The supernatant was collected 48 hours posttransfection and clarified by centrifugation (3,500 *rcf*, 15 minutes). Huh7 cells were subsequently transduced with GeCKO lentiviruses at an MOI of 0.3 and selected for with puromycin at a concentration of 0.25 μg/ml for 7 days. To ensure sufficient sgRNA coverage, 60 Mio selected Huh7 cells were infected with either HCoV-229E (33°C, MOI 0.1) or MERS-CoV (37°C, MOI 0.05) and then incubated until the nontransduced control cells died. Nontransduced Huh7 cells were infected with respective viruses to control for complete cytopathic effect. Both screens were performed in 3 independent biological replicates. Surviving cells were harvested approximately 2 weeks postinfectionm, and genomic DNA was isolated using the Macherey Nagel NucleoSpin Tissue Kit (Macherey Nagel, Düren, Germany) according to the manufacturer’s instructions. All sgRNAs were amplified from genomic DNA using a 2-step PCR protocol, enabling multiplexing and the addition of specific barcodes for Illumina sequencing on a NovaSeq using 60 Mio reads and paired end reads 150 Illumina Adapter Primers [84]:

**Table:** 

PCR1 fwd (F_PCR1_CRSPRv2_1–7)	ACACTCTTTCCCTACACGACGCTCTTCCGATCT*XXXXXX*CTTGTGGAAAGGACGAAACACCGG
PCR1 rev (R_PCR1_CRSPRv2)	GTGACTGGAGTTCAGACGTGTGCTCTTCCGATCTACTGACGGGCACCGGAGCCAATTCC
PCR2 fwd (F_PCR2_CRSPRv2)	AATGATACGGCGACCACCGAGATCTACACTCTTTCCCTACACGACGCTCTTCCGATCT
PCR2 rev (R_PCR2_CRSPRv2_1)	CAAGCAGAAGACGGCATACGAGATATCACGGTGACTGGAGTTCAGACGTGTGCTCTTCCGATCT

PCR products were then purified using Macherey Nagel PCR Clean Up pooled and sequenced on the Illumina NovaSeq 6000 at the NGS facility at the University of Bern. The input library was also sequenced using the Illumina NGS platform to ensure full representation of sgRNAs in the GeCKO library.

#### Computational analysis of genome-wide CRISPR/Cas-9-mediated KO screens

Demultiplexed FASTQ files were trimmed and aligned to the reference sequences in the sgRNA library file. sgRNA abundance was quantified using the “count” command from the MAGeCK pipeline, and counts were compared between uninfected and infected samples to determine positive enrichment scores for each gene. MAGeCK testing was performed using paired analysis and the RRA algorithm. Two different methods (“alpha mean” and “second best”) were used to calculate gene-level LFCs between samples. The “alpha mean” method calculates gene-level LFC by determining the mean LFC value of sgRNAs ranked in front of the alpha cutoff in RRA, while the “second-best” method uses the LFC of the second strongest sgRNA for a particular gene as the gene-level LFC [23]. Genes with a RRA *p*-value of ≤0.05 and a LFC of ≥2 using both LFC methods were considered significantly enriched. For both CoV screens, data from 3 independent biological replicates were used as the input for data analysis. The GO enrichment was performed on significantly enriched genes from each CoV screen using the “compareCluster” function in clusterProfiler with the “fun” option set to “enrichGO” and a formula of “Entrez ~ Screen.” To reduce GO term redundancy and identify a representative GO term for groups of similar terms, the rrvgo package was used in R with the similarity threshold set to 0.75. Finally, the plot in Fig 2A was created using the “emapplot_cluster” function in the enrichplot package with a filtered version of the compareCluster enrichment result (filtered to include representative GO terms found in both CoV screens that contained 1 or more of the 19 common significantly enriched genes). All heatmaps were generated using the pheatmap package in R with clustering distance set to “Euclidean” and using the complete linkage clustering method. Volcano plots and Venn diagrams were created via the EnhancedVolcano and VennDiagram packages in R, respectively.

### Characterization and analysis of top scoring HDFs

#### ACE2 expression, FKBP8, TMEM41B, and MINAR1 KO in Huh7 cells

pSCRPSY-Tag-RFP-ACE2 (kindly provided by John Schoggins) was used for lentivirus production as described above, and Huh7 cells were transduced and selected for using 0.5 ug/ml Blasticidin. ACE2 expression was confirmed via RFP expression. sgRNAs with highest scores in CRISPR KO screen were ordered as forward and reverse oligos for creation of stable KO cell lines.

**Table:** 

HFNA1_FWD	GTCGTCTCCCACCGAGACACGCACCTCCGTGACGGTTTCGAGACGTG
ATP9B_FWD	GTCGTCTCCCACCGAAGAGTTCAGACATACAAGTGTTTCGAGACGTG
CDH7_FWD	GTCGTCTCCCACCGGGTCCCGGACCAAGCGCAGCGTTTCGAGACGTG
FAM110B_FWD	GTCGTCTCCCACCGTCTCCACGTCCGCGTCCACTGTTTCGAGACGTG
GUCY2C_FWD	GTCGTCTCCCACCGGTGAAGGCCTCGACCTACTCGTTTCGAGACGTG
KIAA1024_FWD	GTCGTCTCCCACCGTGCACGGAATGCGGGCGACAGTTTCGAGACGTG
MAP3K11_FWD	GTCGTCTCCCACCGCTTCGACGAGCTGCGAGCCAGTTTCGAGACGTG
OR9K2_FWD	GTCGTCTCCCACCGCCATTATTATGACTGATCCTGTTTCGAGACGTG
PCTP_FWD	GTCGTCTCCCACCGGATCGAGAGTGACGGCAAGAGTTTCGAGACGTG
C7orf50_FWD	GTCGTCTCCCACCGGAGGGCCCAGCGCATCCGACGTTTCGAGACGTG
DIO1_FWD	GTCGTCTCCCACCGCTGCCTGCAGGCGATCCTGAGTTTCGAGACGTG
ECI2_FWD	GTCGTCTCCCACCGCCTTGTAACATGCCCAAACCGTTTCGAGACGTG
ELFN2_FWD	GTCGTCTCCCACCGGTGCCGTGCGTGCCGACTGCGTTTCGAGACGTG
GLCCI1_FWD	GTCGTCTCCCACCGAATAAGGCGAACCTCCTCTTGTTTCGAGACGTG
HOXB6_FWD	GTCGTCTCCCACCGAGACATTACCCCGCGCCCTAGTTTCGAGACGTG
KAT7_FWD	GTCGTCTCCCACCGGACAACTCACCATGTGCCGGGTTTCGAGACGTG
NOM1_FWD	GTCGTCTCCCACCGGGAGTTCGTGCACGCGACTTGTTTCGAGACGTG
PIGR_FWD	GTCGTCTCCCACCGGCAGGAAGGCTCGCCTATCCGTTTCGAGACGTG
TIGD1_FWD	GTCGTCTCCCACCGTATACTTACTCACTAAGCTGGTTTCGAGACGTG
TMEM41B_FWD	GTCGTCTCCCACCGTATACTTACTCACTAAGCTGGTTTCGAGACGTG
ART1_FWD	GTCGTCTCCCACCGGGGCCACCCCATGCTCATCGGTTTCGAGACGTG
CD1C_FWD	GTCGTCTCCCACCGTCGAGTAATCTTGACTTGCAGTTTCGAGACGTG
FKBP8_FWD	GTCGTCTCCCACCGCGTACATCTGCAGACGTCGCGTTTCGAGACGTG
GIMAP4_FWD	GTCGTCTCCCACCGGCGACAATGGCAGCCCAATAGTTTCGAGACGTG
WNT5A_FWD	GTCGTCTCCCACCGAGTATCAATTCCGACATCGAGTTTCGAGACGTG
ZNF480_FWD	GTCGTCTCCCACCGTCACTTACATCTGTCTGAACGTTTCGAGACGTG
KRTAP13-4_FWD	GTCGTCTCCCACCGAGAAATCCTGCTACCGCCCCGTTTCGAGACGTG
HFNA1_REV	CACGTCTCGAAACCGTCACGGAGGTGCGTGTCTCGGTGGGAGACGAC
ATP9B_REV	CACGTCTCGAAACACTTGTATGTCTGAACTCTTCGGTGGGAGACGAC
CDH7_REV	CACGTCTCGAAACGCTGCGCTTGGTCCGGGACCCGGTGGGAGACGAC
FAM110B_REV	CACGTCTCGAAACAGTGGACGCGGACGTGGAGACGGTGGGAGACGAC
GUCY2C_REV	CACGTCTCGAAACGAGTAGGTCGAGGCCTTCACCGGTGGGAGACGAC
KIAA1024_REV	CACGTCTCGAAACTGTCGCCCGCATTCCGTGCACGGTGGGAGACGAC
MAP3K11_REV	CACGTCTCGAAACTGGCTCGCAGCTCGTCGAAGCGGTGGGAGACGAC
OR9K2_REV	CACGTCTCGAAACAGGATCAGTCATAATAATGGCGGTGGGAGACGAC
PCTP_REV	CACGTCTCGAAACTCTTGCCGTCACTCTCGATCCGGTGGGAGACGAC
C7orf50_REV	CACGTCTCGAAACGTCGGATGCGCTGGGCCCTCCGGTGGGAGACGAC
DIO1_REV	CACGTCTCGAAACTCAGGATCGCCTGCAGGCAGCGGTGGGAGACGAC
ECI2_REV	CACGTCTCGAAACGGTTTGGGCATGTTACAAGGCGGTGGGAGACGAC
ELFN2_REV	CACGTCTCGAAACGCAGTCGGCACGCACGGCACCGGTGGGAGACGAC
GLCCI1_REV	CACGTCTCGAAACAAGAGGAGGTTCGCCTTATTCGGTGGGAGACGAC
HOXB6_REV	CACGTCTCGAAACTAGGGCGCGGGGTAATGTCTCGGTGGGAGACGAC
KAT7_REV	CACGTCTCGAAACCCGGCACATGGTGAGTTGTCCGGTGGGAGACGAC
NOM1_REV	CACGTCTCGAAACAAGTCGCGTGCACGAACTCCCGGTGGGAGACGAC
PIGR_REV	CACGTCTCGAAACGGATAGGCGAGCCTTCCTGCCGGTGGGAGACGAC
TIGD1_REV	CACGTCTCGAAACCAGCTTAGTGAGTAAGTATACGGTGGGAGACGAC
TMEM41B_REV	CACGTCTCGAAACCAGCTTAGTGAGTAAGTATACGGTGGGAGACGAC
ART1_REV	CACGTCTCGAAACCGATGAGCATGGGGTGGCCCCGGTGGGAGACGAC
CD1C_REV	CACGTCTCGAAACTGCAAGTCAAGATTACTCGACGGTGGGAGACGAC
FKBP8_REV	CACGTCTCGAAACGCGACGTCTGCAGATGTACGCGGTGGGAGACGAC
GIMAP4_REV	CACGTCTCGAAACTATTGGGCTGCCATTGTCGCCGGTGGGAGACGAC
WNT5A_REV	CACGTCTCGAAACTCGATGTCGGAATTGATACTCGGTGGGAGACGAC
ZNF480_REV	CACGTCTCGAAACGTTCAGACAGATGTAAGTGACGGTGGGAGACGAC
KRTAP13-4_REV	CACGTCTCGAAACGGGGCGGTAGCAGGATTTCTCGGTGGGAGACGAC

Oligonucleotides were denatured for 5 minutes at 99°C in TE buffer and then slowly adapted to room temperature (RT) and assembled with pLentiCRISPRv2 vector using Golden Gate cloning. Plasmids were transformed in Stellar cells (Takara, Kusatsu, Shiga, Japan) and prepped for Sanger sequencing and lentivirus production. ACE2-expressing Huh7 cells were transduced with pLentiCRISPRv2 containing sgRNAs for top scoring hits and selected with 0.25 ug/ml puromycin. Bulk KO of FKBP8, TMEM41B, and MINAR1 was verified using Sanger sequencing and western blot.

### Western blot

A total of 500,000 cells were lysed in M-PER Mammalian Protein Extraction Reagent (Thermo Fisher Scientific 78501) containing 1x protease inhibitor (cOmplete Tablets, Mini EDTA-free, EASYpack, Roche (Basel, Switzerland), 04693159001), mixing at 600 rpm for 10 minutes at RT in a ThermoMixer. Lysed cells were denatured with SDS at 95°C for 5 minutes and separated on an 10% SDS PAGE (SurePAGE Bis-Tris, 10x8, GenScript (Piscataway, New Jersey, United States), M00666) at 200 V for 30 minutes. eBlot L1—Fast Wet Protein Transfer System (GenScript, Piscataway, New Jersey, United States) was used for blotting, and proteins were stained using the following antibodies: FKBP8 (Sigma-Aldrich (St. Louis, Missouri, USA), AV46863), TMEM41B (Cell Signalling Technology (Danvers, Massachusetts, USA), #68071), GFP (Invitrogen (Waltham, Massachusetts, USA), A11122), LC3B (Sigma-Aldrich (St. Louis, Missouri, USA), L7543), p62 (Abcam (Cambridge, UK), 91526), β-Actin-HRP (Sigma-Aldrich (St. Louis, Missouri, USA), A3854), as well as donkey anti rabbit-HRP (Jackson ImmunoResearch (West Grove, Pennsylvania, USA), 711-035-152). Proteins were visualized using WesternBright ECL HRP substrate (Advansta (San Jose, California, USA), K-12045-D20) and the Fusion FX (Vilber, Collégien, France) imaging system. Protein band quantification was performed using the Fusion Software, Copyright 2004 to 2018 by Vilber Lourmat SAS.

### Propagation-competent chimeric VSV harboring the spike proteins of MERS-CoV, HCoV-229E, or SARS-CoV-2

The propagation-competent chimeric viruses VSV*ΔG(MERS S) and VSV*ΔG(229E S), which express the eGFP reporter along with the spike proteins of MERS-CoV and HCoV-229E, respectively, have been recently described [31]. VSV-SARS-CoV-2-spike-eGFP (S gene of SARS-CoV-2 isolate Wuhan-Hu-1: GenBank MN908947.3) was kindly provided by Sean Whelan.

### VSV pseudotype particles bearing CoV spike proteins

Approximately 6*10^5^ 293LTV cells were seeded into a 6-well plate and transfected with expression plasmids encoding either the VSV G envelope protein (positive control, VSV-G; GenBank accession number NC_001560), HCoV-229E spike protein (pCAGGS-229E S; GenBank accession number X16816), MERS-CoV spike protein (pCAGGS-MERS S; GenBank accession number JX869059, with the silent point mutation C4035A removing an internal XhoI endonuclease restriction site), SARS-CoV spike (pCAGGS-SARS S; GenBank accession number: AY291315.1, with 2 silent mutations T2568G and T3327C), or SARS-CoV-2 spike [31] using the transfection reagent Lipofectamine 2000 as described previously [31]. At 20 hours posttransfection, cells were infected at 37°C with VSV*ΔG(FLuc) (MOI of 5 ffu/cell), which has been *trans*-complemented with VSV-G protein. After an inoculation time of 30 minutes, the cells were washed with PBS and incubated for 24 hours with DMEM medium containing a neutralizing monoclonal antibody directed to the VSV-G protein (clone I1, ATCC, 1:100). The cell culture supernatant was harvested and cleared by centrifugation (3,000 g for 10 minutes) and used to inoculate Huh7 native and KO cell lines for 24 hours, prior to measurement of luciferase using Bright-Glo Luciferase Assay System (Promega, E2620) and using a plate luminometer (EnSpire 2300 Multilabel reader; Perkin Elmer, Waltham, Massachussetts, USA).

### Viruses

HCoV-229E [78] was propagated on Huh7 cells. MERS-CoV strain EMC [79] was propagated in VeroB4 cells. SARS-CoV strain Frankfurt-1 [80] and SARS-CoV-2 (SARS-CoV-2/München-1.1/2020/929, kindly provided by Daniela Niemeyer, Marcel Müller, and Christian Drosten) were propagated on VeroE6 cells. CDV recombinant strain A75/17_nlucP was propagated as described previously [85].

### Virus infection

Huh7 cells were plated to 15,000 cells, and VeroE6 cells were plated to 20,000 per 96 well 24 hours prior to infection. Cells were infected with HCoV-229E (33°C), MERS-CoV (37°C), SARS-CoV (37°C), and SARS-CoV-2 (37°C) at an MOI of 0.01 (MOI 0.1 for HCoV-229E) for 2 hours. The virus inoculum was removed, and cells were washed 3 times with PBS. Primary human nasal epithelial cell cultures were infected with SARS-CoV-2 at an MOI of 0.1 at 37°C for 1 hour from the apical side. Inoculum was removed and cell 3 times with HBBS. In case of inhibitor treatment, cyclosporin A or alisporivir were added to the cell supernatant/basolateral medium directly after the removal of the inoculum and the washing of the cells at following concentrations: 0 uM, 10 uM, 20 uM, 30 uM, 40 uM, 50 uM, and 60 uM. DMSO solvent control was added at respective volumes. The inhibitor was not removed during the course of infection. At 24 to 48 hours postinfection, the cells/supernatant were/was harvested and analyzed using titration, immunofluorescence staining, or quantitative RT-PCR (qRT-PCR). For the virus infection experiments using CDV, the recombinant strain A75/17_nlucP was used and treated with inhibitors as mentioned above. Virus replication was determined using the NanoLuc luciferase tag as previously described [85].

### Virus titration

In order to determine the TCID_50_ per milliliter (apical), supernatant was serially diluted at indicated hours postinfection, Huh7 (MERS-CoV and HCoV-229E) VeroE6 cells (SARS-CoV(-2)) were inoculated with serial dilution, and TCID_50_ per milliliter was visualized using Crystal Violet and calculated by the Spearman–Kärber algorithm after 72 hours to 120 hours as described [86].

### qRT-PCR

Virus replication was analyzed via qRT-PCR, and viral RNA was isolated from the supernatant at indicated hours postinfection using the NucleoMag Vet Kit (Macherey Nagel) and a Kingfisher Flex Purification System (Thermo Fisher Scientific) according to the manufacturer’s guidelines. Extracted RNA was amplified using TagMan Fast Virus 1-Step Master Mix (Thermo Fisher Scientific). The following primers were used for detection of MERS-CoV [87]:

**Table:** 

forward	5′-GCAACGCGCGATTCAGTT-3′
reverse	5′-GCCTCTACACGGGACCCATA-3′
probe	5′-FAM-CTCTTCACATAATCGCCCCGAGCTCG-BHQ1—3′

and SARS-CoV and SARS-CoV-2:

**Table:** 

forward	5′-ACAGGTACGTTAATAGTTAATAGCGTACTTCT-3′
reverse	5′-ATATTGCAGCAGTACGCACACA-3′
probe	5′-FAM-ATCCTTACTGCGCTTCGA-BHQ1-3′

targeting the Envelope gene of SARS-CoV-2 (MN908947.3). The primers were adapted from Corman and colleagues [88]. A serial dilution of in vitro transcribed (IVT) MERS-CoV RNA (kindly provided by Marcel Müller and Christian Drosten) [87] and RdRp-E-N RNA mixture derived from a SARS-CoV-2 synthetic construct (MT108784) was included to determine the genome copy number [89]. Five IVT RNA preparations were produced from 5 different DNA fragments to cover the regions used for real-time RT-qPCR methods for the detection of SARS-CoV-2 and SARS-CoV viral RNA. Measurements and analysis were performed with the Applied Biosystems 7500 Fast Dx Real-Time PCR Systems and associated software (Applied Biosystems, Foster City, California, USA).

### Immunofluorescence staining

For immunofluorescence staining, cells were fixated with 4% formalin. Fixated cells were permeabilized in PBS supplemented with 50 mM NH_4_Cl, 0.1% (w/v) Saponin, and 2% (w/v) Bovine Serum Albumin and stained with a mouse monoclonal antibody against dsRNA (SCICONS (Hungary), clone J2). Alexa-Fluor 488-labeled donkey-anti mouse IgG (H+L) (Jackson ImmunoResearch, 715-545-150) was used as a secondary antibody. Alexa-Fluor 647-labeled rabbit anti-beta-tubulin IV (Cell Signalling Technology, 9F3) and Alexa-Fluor 594-labeled mouse anti ZOI-1 (Thermo Fisher Scientific, 1A12) were used to visualize cilia and tight junctions in nasal epithelial cell cultures. Cells were counterstained using DAPI (Thermo Fisher Scientific) to visualize the nuclei. Images were acquired using an EVOS FL Auto 2 Imaging System, using 10×, 20×, and 40× air objectives. Brightness and contrast were adjusted identically to the corresponding controls using the Fiji software packages [90], and figures were assembled using FigureJ [91]. Segmentation of individual cells was based on the ZO-1 staining and performed using CellPose [92]. Outlines were imported and overlaid in Fiji.

### Cytotoxicity and cell viability assay

Cytotoxicity in Huh7 KO cell lines and upon inhibitor treatment of Huh7 and VeroE6 cell lines was monitored using CytoTox 96 Non-Radioactive Cytotoxicity Assay (Promega, G1780). Relative cytotoxicity compared to lysed control cells was analyzed. Cell viability of primary human nasal epithelial cells was analyzed during inhibitor only treatment at highest concentrations (50 uM and 60 uM) using the CellTiter-Glo 2.0 Cell Viability Assay (Promega, G9241) and related to DMSO treated cells.

### LC3-GFP autophagy assay, LC3II, and p62 analysis during rapamycin treatment and HCoV-229E infection

Autophagosome formation was assessed in native Huh7 and Huh7-KO cell lines. Huh7, TMEM41B-KO, MINAR1-KO, and FKBP8-KO cells were seeded in a 96-well formation (1.5 Mio cells per plate). LC3-GFP was transfected using Lipofectamine 2000 for 24 hours. After 24 hours, cells were treated with 100 nM rapamycin (Sigma-Aldrich, S-015) or an equal volume of DMSO for 6 hours, and GFP was analyzed using an EVOS FL Auto 2 Imaging System, using 10× and processed as mentioned above. Alternatively, transfected cells were infected with HCoV-229E at an MOI 0.1 for 24 hours, and GFP expression was analyzed. Images were quantified for autophagosome formation by manual counting using 5 images per condition and 3 replicates in Fiji. Autophagosome formation was normalized to number of transfected cells. In order to determine LC3II and p62 protein expression, 1 Mio cells were seeded in a 24 well plate at day 0. At day 1, cells were infected with HCoV-229E at an MOI of 1 for 24 hours or treated with rapamycin at a final concentration of 100 pmol/ml for 6 hours. At indicated time points, cells were lysed for western blotting as mentioned in the respective method section.

### Quantification and statistical analysis

#### Genome-wide CRISPR/Cas-9-mediated KO screen

For the CRISPR screens, positive enrichment scores, RRA *p*-values, LFC, and false discovery rates were calculated using the MAGeCK algorithm. In S1B Fig, the mean normalized sgRNA counts for each biological replicate were used as input to calculate pairwise correlation. The correlation matrix was generated using the “cor” function in R with the Pearson correlation method and visualized using pheatmap with the clustering performed using correlation as distance metrics.

#### Characterization and analyses of top scoring HDFs

Significant difference in data was tested using Nev 2020, version 9.0 or GraphPad Prism version 8.3.1 for Windows (GraphPad, San Diego, California, USA). Please refer to figure captions for details regarding the statistical tests applied. *p*-Values <0.05 were considered significant.

#### Additional resources

No additional resources have been created during this study.

## Supporting information

S1 Fig Quality control metrics and enriched gene identification for MERS-CoV and HCoV-229E genome-wide CRISPR screens. **(A)** AUC analysis of MERS-CoV and HCoV-229E CRISPR screens evaluating sgRNA library representation in surviving Huh7 cells from uninfected (Mock) and MERS-CoV (left 2 panels) or HCoV-229E (right 2 panels) infected samples. For each CRISPR screen, sgRNA abundance was calculated based on average sgRNA abundance over 3 independent biological replicates. Raw data for calculations can be found in Supporting information S1 Data, tabs 3 and 4. **(B)** Correlation matrix depicting the Pearson correlation for guide-level normalized read counts among biological replicates and samples from both screens. R1, R2, and R3 represent the biological replicates 1, 2, and 3, respectively. Clustering was performed in pheatmap using correlation as a distance metric. Raw data can be found in Supporting information S1 Data, tab 5. **(C)** RRA *p*-value distribution of all genes in the GeCKOv2 library for both MERS-CoV (left) and HCoV-229E (right) CRISPR screens. Genes that met the criteria for significance (RRA *p*-value ≤0.05 and FC ≥ 2) are highlighted in red. Raw data can be found in Supporting information S1 Data, tab 1. **(D)** Venn diagram illustrating the overlap between significantly enriched genes from both CRISPR screens that were identified via 2 different RRA-based analysis methods (alpha median and second best). A total of 19 genes were identified by both methods in both MERS-CoV and HCoV-229E CRISPR screens. Raw data can be found in Supporting information S1 Data, tab 1. AUC, area under the curve; HCoV, human coronavirus; MERS-CoV, Middle East Respiratory Syndrome Coronavirus; RRA, robust rank aggregation; sgRNA, single guide RNA.
(TIF)

S2 Fig GO of MERS-CoV and HCoV-229E host factors. **(A)** Representative GO terms identified using full list of enriched GO terms for MERS-CoV and HCoV-229E screens (S2 Table). Representative terms found in both screens are shown in the top panel, whereas virus-specific terms are shown in the bottom panel. BP, CC, and MF represent different GO term categories. **(B)** Specific GO terms enriched in both CoV screens (individual GO terms, not representative GO terms). Raw data can be found in S2 Table. CoV, coronavirus; GO, Gene Ontology; HCoV, human coronavirus; MERS-CoV, Middle East Respiratory Syndrome Coronavirus.
(TIF)

S3 Fig Cnet plot for biological clusters up-regulated in the MERS-CoV and HCoV-229E. Cnet plot for the Golgi vesicle transport cluster shown in Fig 2A. The plot includes both GO terms that contain 1 or more of the 19 common significantly enriched genes found in both CoV screens (as in Fig 2A and 2B) as well as representative GO terms found in both screens that do not contain these genes. The plot shows the relationship among individual GO terms and genes found in the Golgi vesicle transport cluster. Larger nodes represent individual GO terms, and smaller nodes represent individual gene. Nodes that are functionally related cluster together into a larger network. Node size reflects the number of significantly enriched genes in the node, and color indicates the CoV screen for which the node was significant. Raw data can be found in S2 Table. CoV, coronavirus; GO, Gene Ontology; HCoV, human coronavirus; MERS-CoV, Middle East Respiratory Syndrome Coronavirus.
(TIFF)

S4 Fig Cnet plot for biological clusters up-regulated in the MERS-CoV and HCoV-229E. Cnet plot for the autophagy cluster shown in Fig 2A. The plot includes both GO terms that contain 1 or more of the 19 common significantly enriched genes found in both CoV screens (as in Fig 2A and 2B) as well as representative GO terms found in both screens that do not contain these genes. Each plot shows the relationship among individual GO terms and genes found in each biological cluster. Larger nodes represent individual GO terms, and smaller nodes represent individual gene. Nodes that are functionally related cluster together into a larger network. Node size reflects the number of significantly enriched genes in the node, and color indicates the CoV screen for which the node was significant. Raw data can be found in S2 Table. CoV, coronavirus; GO, Gene Ontology; HCoV, human coronavirus; MERS-CoV, Middle East Respiratory Syndrome Coronavirus.
(TIF)

S5 Fig Cnet plot for biological clusters up-regulated in the MERS-CoV and HCoV-229E. Cnet plot for the catabolic processes cluster shown in Fig 2A. Plots include both GO terms that contain 1 or more of the 19 common significantly enriched genes found in both CoV screens (as in Fig 2A and 2B) as well as representative GO terms found in both screens that do not contain these genes. The plot shows the relationship among individual GO terms and genes found in the catabolic processes cluster. Larger nodes represent individual GO terms, and smaller nodes represent individual gene. Nodes that are functionally related cluster together into a larger network. Node size reflects the number of significantly enriched genes in the node, and color indicates the CoV screen for which the node was significant. Raw data can be found in S2 Table. CoV, coronavirus; GO, Gene Ontology; HCoV, human coronavirus; MERS-CoV, Middle East Respiratory Syndrome Coronavirus.
(TIF)

S6 Fig Cnet plot for biological clusters up-regulated in the MERS-CoV and HCoV-229E. Cnet plot for the dephosphorylation cluster shown in Fig 2A. The plot includes both GO terms that contain 1 or more of the 19 common significantly enriched genes found in both CoV screens (as in Fig 2A and 2B) as well as representative GO terms found in both screens that do not contain these genes. The plot shows the relationship among individual GO terms and genes found in the dephosphorylation cluster. Larger nodes represent individual GO terms, and smaller nodes represent individual gene. Nodes that are functionally related cluster together into a larger network. Node size reflects the number of significantly enriched genes in the node, and color indicates the CoV screen for which the node was significant. Raw data can be found in S2 Table. CoV, coronavirus; GO, Gene Ontology; HCoV, human coronavirus; MERS-CoV, Middle East Respiratory Syndrome Coronavirus.
(TIFF)

S7 Fig Cnet plot for biological clusters up-regulated in the MERS-CoV and HCoV-229E. Cnet plot for the immunity cluster shown in Fig 2A. The plot includes both GO terms that contain 1 or more of the 19 common significantly enriched genes found in both CoV screens (as in Fig 2A and 2B) as well as representative GO terms found in both screens that do not contain these genes. The plot shows the relationship among individual GO terms and genes found in the immunity cluster. Larger nodes represent individual GO terms, and smaller nodes represent individual gene. Nodes that are functionally related cluster together into a larger network. Node size reflects the number of significantly enriched genes in the node, and color indicates the CoV screen for which the node was significant. Raw data can be found in S2 Table. CoV, coronavirus; GO, Gene Ontology; HCoV, human coronavirus; MERS-CoV, Middle East Respiratory Syndrome Coronavirus.
(TIFF)

S8 Fig Cnet plot for biological clusters up-regulated in the MERS-CoV and HCoV-229E. Cnet plot for the developmental processes cluster shown in Fig 2A. The plot includes both GO terms that contain 1 or more of the 19 common significantly enriched genes found in both CoV screens (as in Fig 2A and 2B) as well as representative GO terms found in both screens that do not contain these genes. The plot shows the relationship among individual GO terms and genes found in the developmental processes cluster. Larger nodes represent individual GO terms, and smaller nodes represent individual gene. Nodes that are functionally related cluster together into a larger network. Node size reflects the number of significantly enriched genes in the node, and color indicates the CoV screen for which the node was significant. Raw data can be found in S2 Table. CoV, coronavirus; GO, Gene Ontology; HCoV, human coronavirus; MERS-CoV, Middle East Respiratory Syndrome Coronavirus.
(TIFF)

S9 Fig Cnet plot for biological clusters up-regulated in the MERS-CoV and HCoV-229E. Cnet plot for the homeostatic processes cluster shown in Fig 2A. The plot includes both GO terms that contain 1 or more of the 19 common significantly enriched genes found in both CoV screens (as in Fig 2A and 2B) as well as representative GO terms found in both screens that do not contain these genes. The plot shows the relationship among individual GO terms and genes found in the homeostatic processes cluster. Larger nodes represent individual GO terms, and smaller nodes represent individual gene. Nodes that are functionally related cluster together into a larger network. Node size reflects the number of significantly enriched genes in the node, and color indicates the CoV screen for which the node was significant. Raw data can be found in S2 Table. CoV, coronavirus; GO, Gene Ontology; HCoV, human coronavirus; MERS-CoV, Middle East Respiratory Syndrome Coronavirus.
(TIFF)

S10 Fig CRISPR-mediated KO of top scoring HDFs impairs CoV replication. **(A)** Immunofluorescence staining of MERS-CoV infected of Huh7 cells containing KO of top scoring HDFs. dsRNA is shown in green, and DAPI is shown in blue. **(B)** Western blot analysis quantification of LC3II and p62 expression. Protein expression is normalized to beta actin and relative to the untreated condition in WT and FKBP8-KO cell treated with rapamycin or infected with HCoV-229E. Quantification data can be found in Supporting information S1 Data, tab 15. Western blot raw images are depicted in S1 Raw Images. **(C)** Western blot analysis quantification of LC3II expression normalized to beta actin and relative to WT. Protein expression is depicted in TMEM41B-, MINAR1-, and FKBP8-KO cells after treatment with rapamycin and HCoV-229E infection. Statistical significance was determined using the Holm–Sidak method in GraphPad Prism. *p*-Values are indication, and *p*-values <0.05 are defined as statistically significant. Quantification data can be found in Supporting information S1 Data, tab 16. Western blot raw images are shown in S1 Raw Images. **(D)** Immunofluorescence staining of HCoV-229E, SARS-CoV, and SARS-CoV-2 infected Huh7 cells with TMEM41B, FKBP8, and MINAR1-KO, as well as a stable ACE2 expression. dsRNA is shown in green, DAPI is shown in blue, and ACE2 is shown in red. Scale bar is 50 μm. All images were acquired using an Evos Auto FL2 and processed in Fiji. **(E)** Relative cytotoxicity of TMEM41-KO, FKBP8-KO, and MINAR1-KO is depicted in %. Two-tailed unpaired Student *t* test was used to determine significance in GraphPad Prism 8.3.1. **(F)** Sanger sequencing of FKBP8-KO, MINAR1-KO, and TMEM41B-KO verifies Cas-9–mediated double-strand break in multiple alleles of the KO cells. PAM sequence is indicated in red, and binding site of sgRNA is indicated in blue. Used reagents are listed in detail in Table 1. ACE2, angiotensin converting enzyme 2; CoV, coronavirus; FKBP8, FK506 binding protein 8; HCoV, human coronavirus; HDF, host dependency factor; KO, knockout; MERS-CoV, Middle East Respiratory Syndrome Coronavirus; MINAR1, Membrane Integral NOTCH2 Associated Receptor 1; SARS-CoV, Severe Acute Respiratory Syndrome Coronavirus; SARS-CoV-2, Severe Acute Respiratory Syndrome Coronavirus 2; sgRNA, single guide RNA; TMEM41B, transmembrane protein 41B; WT, wild-type.
(TIFF)

S11 Fig Cyclosporine A and alisporivir inhibit CoV infection in a dose-dependent manner in cell lines and primary human nasal epithelial cells at noncytotoxic concentrations. Immunofluorescence staining of SARS-CoV **(A)**, as well as SARS-CoV-2 **(B)** infected VeroE6 cells and MERS-CoV **(C)** infected Huh7 cells following cyclosporine A and alisporivir treatment at 10 μM to 40 μM and bafilomycin A1 treatment at 10 nM to 40 nM, as well as DMSO CTRL at respective volumes 24 hours postinfection/inhibitor treatment. dsRNA is shown in green, and DAPI is shown in blue. Scale bar is 50 μm. All images were acquired using an EVOS FL Auto 2 imaging system with a 10× air objective. **(D)** Relative infectivity of CDV (raw data can be found in Supporting information S1 Data, tab 17) and **(E)** VSVΔG in % after treatment with cyclosporine A (teal) and alisporivir (purple) (raw data can be found in Supporting information S1 Data, tab 18). **(F, G)** Inhibitor treated primary nasal epithelial cell cultures displayed as inhibitor versus normalized response. IC_50_ value is marked with dotted line and indicated on y-axis. Calculations were performed in GraphPad Prism 8.3.1. Raw data can be found in Supporting information S1 Data, tab 19. **(H)** Relative nasal epithelial cell culture viability upon treatment of 60 μM cyclosporine A and alisporivir normalized to DMSO. Raw data can be found in Supporting information S1 Data, tab 20. Cyclosporine A, alisporivir, and bafilomycin A1, treatment-mediated cytotoxicity in Huh7 cells **(J)**, and VeroE6 cells **(K)** shown relative to dead cell control. Raw data can be found in Supporting information S1 Data, tabs 21 and 22. Used reagents are listed in detail in Table 1. MERS-CoV, Middle East Respiratory Syndrome Coronavirus; SARS-CoV, Severe Acute Respiratory Syndrome Coronavirus; SARS-CoV-2, Severe Acute Respiratory Syndrome Coronavirus 2.
(TIF)

S1 Data Data set contain all raw data shown in the main figures and Supporting information figures. Tab 1: Raw data for Figs 1B–1E, 2B, and 4B and S1C and S1D Fig. Tab 2: Raw data for Fig 2A and 2B. Tab 3: Raw data for S1A Fig (MERS-CoV data). Tab 4: Raw data for S1A Fig (HCoV-229E data). Tab 5: Raw data for S1B Fig. Tab 6: Raw data for Fig 3A. Tab 7: Raw data for Fig 3B. Tab 8: Raw data for Fig 3C–3F. Tab 9: Raw data for Fig 3G. Tab 10: Raw data for Fig 3I. Tab 11: Raw data for Fig 4D. Tab 12: Raw data for Fig 5A. Tab 13: Raw data for Fig 5B. Tab 14: Raw data for Fig 5C. Tab 15: Raw data for S4B Fig. Tab 16: Raw data for S4C Fig. Tab 17: Raw data for S11D Fig. Tab 18: Raw data for S11E Fig. Tab 19: Raw data for S11G Fig and S11G Fig. Tab 20: Raw data for S11H Fig. Tab 21: Raw data for S11J Fig. Tab 22: Raw data S11J Fig. HCoV, human coronavirus; MERS-CoV, Middle East Respiratory Syndrome Coronavirus.
(XLSX)

S1 Table MAGeCK results. Paired analyses using the MAGeCK pipeline comparing uninfected and infected samples from each screen. Gene-level scores were computed using sgRNA LFCs to identify KO genes that were significantly enriched in the MERS-CoV and HCoV-229E infected samples. HCoV, human coronavirus; KO, knockout; LFC, log fold change; MERS-CoV, Middle East Respiratory Syndrome Coronavirus; sgRNA, single guide RNA.
(XLSX)

S2 Table GO enrichment analysis. Results from GO enrichment analysis for each screen using the significantly enriched genes identified by the MAGeCK analysis. GO, Gene Ontology.
(XLSX)

S1 Raw Images Data file contains raw images. Page 1 (upper panel): Western blots for Fig 3G, TMEM41B. (Middle panel) Western blots for Fig 3G, FKBP8. (Lower panel) Western blots for Fig 3G, MINAR1. Page 2: Western blots for S10B Fig graph 1. Page 3: Western blots for S10B Fig graph 2. Page 4: Western blots for S10C Fig, rapamycin treatment. Page 5: Western blots for S10C Fig, HCoV-229E infection. FKBP8, FK506 binding protein 8; HCoV, human coronavirus; MINAR1, Membrane Integral NOTCH2 Associated Receptor 1; TMEM41B, transmembrane protein 41B.
(PDF)

## Abbreviations

ACE2: angiotensin converting enzyme 2
ANPEP/APN: aminopeptidase
AUC: area under the curve
BP: biological process
CDH7: cadherin 7
CDV: canine distemper virus
CM: convoluted membrane
CoV: coronavirus
COVID-19: Coronavirus Disease 2019
DMEM: Dulbecco’s Modified Eagle Medium
DMV: double-membrane vesicle
DPP4: dipeptidyl peptidase 4
EBV: Epstein–Barr virus
ERGIC: endoplasmic reticulum–Golgi intermediate compartment
FKBP8: FK506 binding protein 8
GO: Gene Ontology
HBSS: Hanks’ balanced salt solution
HBV: hepatitis B virus
HCoV: human coronavirus
HCV: hepatitis C virus
HDF: host dependency factor
HHV8: human herpesvirus 8
IC_50_: half maximal inhibitory concentration
IRF3: IFN regulatory factor 3
IVT: in vitro transcribed
JNK: c-Jun N-terminal kinase
KO: knockout
LFC: log fold change
MAPK: mitogen-activated protein kinase
MERS-CoV: Middle East Respiratory Syndrome Coronavirus
MHV: mouse hepatitis virus
Mio: Million
MINAR1: Membrane Integral NOTCH2 Associated Receptor 1
MOI: multiplicity of infection
mPTP: mitochondrial permeability transition pore
NGS: next-generation sequencing
PPI: peptidyl-prolyl isomerase
qRT-PCR: quantitative RT-PCR
RIG-I: retinoic acid inducible protein 1
RRA: robust rank aggregation
RT: room temperature
SARS: Severe Acute Respiratory Syndrome
SARS-CoV: Severe Acute Respiratory Syndrome Coronavirus
SARS-CoV-2: Severe Acute Respiratory Syndrome Coronavirus 2
sgRNA: single guide RNA
STRs: short tandem repeat loci
TCID_50_: 50% tissue culture infectious dose
TMEM41B: transmembrane protein 41B
VISA: virus-induced signaling adaptor signaling
VMP1: vacuole membrane protein 1
VSV: vesicular stomatitis virus
WT: wild-type

## References

[1] Hamre D, Procknow JJ. A New Virus Isolated from the Human Respiratory Tract. Proc Soc Exp Biol Med. 1966. doi: 10.3181/00379727-121-30734 4285768

[2] McIntosh K, Dees JH, Becker WB, Kapikian AZ, Chanock RM. Recovery in tracheal organ cultures of novel viruses from patients with respiratory disease. Proc Natl Acad Sci U S A. 1967;57:933–40. doi: 10.1073/pnas.57.4.933 5231356 PMC224637

[3] Van Der Hoek L, Pyrc K, Jebbink MF, Vermeulen-Oost W, RJM B, Wolthers KC, et al. Identification of a new human coronavirus. Nat Med. 2004. doi: 10.1038/nm1024 15034574 PMC7095789

[4] Woo PCY, SKP L, Chu C, Chan K, Tsoi H, Huang Y, et al. Characterization and Complete Genome Sequence of a Novel Coronavirus, Coronavirus HKU1, from Patients with Pneumonia. J Virol. 2005. doi: 10.1128/JVI.79.2.884-895.2005 15613317 PMC538593

[5] Drosten C, Günther S, Preiser W, Van der Werf S, Brodt HR, Becker S, et al. Identification of a novel coronavirus in patients with severe acute respiratory syndrome. N Engl J Med. 2003;348:1967–76. doi: 10.1056/NEJMoa030747 12690091

[6] Zaki AM, van Boheemen S, Bestebroer TM, Osterhaus ADME, Fouchier RAM. Isolation of a Novel Coronavirus from a Man with Pneumonia in Saudi Arabia. N Engl J Med. 2012;367:1814–20. doi: 10.1056/NEJMoa1211721 23075143

[7] Corman VM, Eckerle I, Memish ZA, Liljander AM, Dijkman R, Jonsdottir H, et al. Link of a ubiquitous human coronavirus to dromedary camels. Proc Natl Acad Sci U S A. 2016;113:9864–9. doi: 10.1073/pnas.1604472113 27528677 PMC5024591

[8] Drosten C, Muth D, Corman VM, Hussain R, Al Masri M, HajOmar W, et al. An observational, laboratory-based study of outbreaks of middle east respiratory syndrome coronavirus in Jeddah and Riyadh, Kingdom of Saudi Arabia, 2014. Clin Infect Dis. 2015. doi: 10.1093/cid/ciu812 25323704 PMC4303774

[9] Raj VS, Mou H, Smits SL, DHW D, Müller MA, Dijkman R, et al. Dipeptidyl peptidase 4 is a functional receptor for the emerging human coronavirus-EMC. Nature. 2013;495:251–4. doi: 10.1038/nature12005 23486063 PMC7095326

[10] Yeager CL, Ashmun RA, Williams RK, Cardellichio CB, Shapiro LH, Look AT, et al. Human aminopeptidase N is a receptor for human coronavirus 229E. Nature. 1992. doi: 10.1038/357420a0 1350662 PMC7095410

[11] Li W, Moore MJ, Vasllieva N, Sui J, Wong SK, Berne MA, et al. Angiotensin-converting enzyme 2 is a functional receptor for the SARS coronavirus. Nature. 2003. doi: 10.1038/nature02145 14647384 PMC7095016

[12] Letko M, Marzi A, Munster V. Functional assessment of cell entry and receptor usage for SARS-CoV-2 and other lineage B betacoronaviruses. Nat Microbiol. 2020. doi: 10.1038/s41564-020-0688-y 32094589 PMC7095430

[13] Hoffmann M, Kleine-Weber H, Schroeder S, Krüger N, Herrler T, Erichsen S, et al. SARS-CoV-2 Cell Entry Depends on ACE2 and TMPRSS2 and Is Blocked by a Clinically Proven Protease Inhibitor. Cell. 2020. doi: 10.1016/j.cell.2020.02.052 32142651 PMC7102627

[14] Knoops K, Kikkert M, SHE VDW, Zevenhoven-Dobbe JC, Van Der Meer Y, Koster AJ, et al. SARS-coronavirus replication is supported by a reticulovesicular network of modified endoplasmic reticulum. PLoS Biol. 2008. doi: 10.1371/journal.pbio.0060226 18798692 PMC2535663

[15] Ulasli M, Verheije MH, de Haan CAM, Reggiori F. Qualitative and quantitative ultrastructural analysis of the membrane rearrangements induced by coronavirus. Cell Microbiol. 2010. doi: 10.1111/j.1462-5822.2010.01437.x 20088951 PMC7159092

[16] Oudshoorn D, Rijs K, RWAL L, Groen K, Koster AJ, Snijder EJ, et al. Expression and cleavage of middle east respiratory syndrome coronavirus nsp3-4 polyprotein induce the formation of double-membrane vesicles that mimic those associated with coronaviral RNA replication. mBio. 2017. doi: 10.1128/mBio.01658-17 29162711 PMC5698553

[17] Klumperman J, Locker JK, Meijer A, Horzinek MC, Geuze HJ, Rottier PJ, et al. Coronavirus M proteins accumulate in the Golgi complex beyond the site of virion budding. J Virol. 1994. doi: 10.1128/JVI.68.10.6523-6534.1994 8083990 PMC237073

[18] Ghosh S, Dellibovi-Ragheb TA, Kerviel A, Pak E, Qiu Q, Fisher M, et al. β-Coronaviruses Use Lysosomes for Egress Instead of the Biosynthetic Secretory Pathway. Cell. 2020. doi: 10.1016/j.cell.2020.10.039 33157038 PMC7590812

[19] Sanjana NE, Shalem O, Zhang F. Improved vectors and genome-wide libraries for CRISPR screening. Nat Methods. 2014. doi: 10.1038/nmeth.3047 25075903 PMC4486245

[20] Schneider WM, Shu T, Wu D, Mu J, Wang C, Huang M, et al. Genome-Scale Identification of SARS-CoV-2 and Pan-coronavirus Host Factor Networks. Cell. 2021. doi: 10.1016/j.cell.2020.12.006 33382968 PMC7796900

[21] Ren Y et al. The ORF3a protein of SARS-CoV-2 induces apoptosis in cells. Cell Mol Immunol. 2020;17. doi: 10.1038/s41423-020-0485-9 32555321 PMC7301057

[22] Hoffmann HH, Luna JM, Hoffmann HH, Sánchez-Rivera FJ, Leal AA, et al. Functional interrogation of a SARS-CoV-2 host protein interactome identifies unique and shared coronavirus host factors. Cell Host Microbe. 2020. doi: 10.1016/j.chom.2020.12.009 33357464 PMC7833927

[23] Li W, Xu H, Xiao T, Cong L, Love MI, Zhang F, et al. MAGeCK enables robust identification of essential genes from genome-scale CRISPR/Cas9 knockout screens. Genome Biol. 2014. doi: 10.1186/s13059-014-0554-4 25476604 PMC4290824

[24] Kleine-Weber H, Elzayat MT, Hoffmann M, Pöhlmann S. Functional analysis of potential cleavage sites in the MERS-coronavirus spike protein. Sci Rep. 2018. doi: 10.1038/s41598-018-34859-w 30413791 PMC6226446

[25] Cao B, Zhang L, Liu H, Ma S, Mi K. The Dynamic Expression of Potential Mediators of Severe Acute Respiratory Syndrome Coronavirus 2 Cellular Entry in Fetal, Neonatal, and Adult Rhesus Monkeys. Front Genet. 2021. doi: 10.3389/fgene.2020.607479 33537060 PMC7848180

[26] Zeng X, Vonk JM, van der Plaat DA, Faiz A, Paré PD, Joubert P, et al. Genome-wide interaction study of gene-by-occupational exposures on respiratory symptoms. Environ Int. 2019. doi: 10.1016/j.envint.2018.11.017 30449631

[27] Bühling F, Waldburg N, Reisenauer A, Heimburg A, Golpon H, Welte T, et al. Lysosomal cysteine proteases in the lung: Role in protein processing and immunoregulation. Eur Respir J. 2004. doi: 10.1183/09031936.04.00105304 15083765

[28] Morita K, Hama Y, Izume T, Tamura N, Ueno T, Yamashita Y, et al. Genome-wide CRISPR screen identifies TMEM41B as a gene required for autophagosome formation. J Cell Biol. 2018. doi: 10.1083/jcb.201804132 30093494 PMC6219718

[29] Moretti F, Bergman P, Dodgson S, Marcellin D, Claerr I, Goodwin JM, et al. TMEM 41B is a novel regulator of autophagy and lipid mobilization. EMBO Rep. 2018. doi: 10.15252/embr.201845889 30126924 PMC6123663

[30] Shoemaker CJ, Huang TQ, Weir NR, Polyakov NJ, Schultz SW, Denic V, et al. CRISPR screening using an expanded toolkit of autophagy reporters identifies TMEM41B as a novel autophagy factor. PLoS Biol. 2019. doi: 10.1371/journal.pbio.2007044 30933966 PMC6459555

[31] Pfaender S, Mar KB, Michailidis E, Kratzel A, Boys IN, V’kovski P, et al. LY6E impairs coronavirus fusion and confers immune control of viral disease. Nat Microbiol. 2020. doi: 10.1038/s41564-020-0769-y 32704094 PMC7916999

[32] Case JB, Rothlauf PW, Chen RE, Liu Z, Zhao H, Kim AS, et al. Neutralizing Antibody and Soluble ACE2 Inhibition of a Replication-Competent VSV-SARS-CoV-2 and a Clinical Isolate of SARS-CoV-2. Cell Host Microbe. 2020. doi: 10.1016/j.chom.2020.06.021 32735849 PMC7332453

[33] Bhujabal Z, Birgisdottir ÅB, Sjøttem E, Brenne HB, Øvervatn A, Habisov S, et al. FKBP8 recruits LC3A to mediate Parkin-independent mitophagy. EMBO Rep. 2017. doi: 10.15252/embr.201643147 28381481 PMC5452039

[34] Zhang H, Zhang Q, Gao G, Wang X, Wang T, Kong Z, et al. UBTOR/KIAA1024 regulates neurite outgrowth and neoplasia through mTOR signaling. PLoS Genet. 2018. doi: 10.1371/journal.pgen.1007583 30080879 PMC6095612

[35] Kabeya Y, Mizushima N, Ueno T, Yamamoto A, Kirisako T, Noda T, et al. LC3, a mammalian homologue of yeast Apg8p, is localized in autophagosome membranes after processing. EMBO J. 2000. doi: 10.1093/emboj/19.21.5720 11060023 PMC305793

[36] Inoue K, Sekiyama K, Yamada M, Watanabe T, Yasuda H, Yoshiba M, et al. Combined interferon α2b and cyclosporin A in the treatment of chronic hepatitis C: Controlled trial. J Gastroenterol. 2003. doi: 10.1007/s00535-002-1104-5 12825133

[37] Liu J, Lane WS, Friedman J, Weissman I, Schreiber SL. Calcineurin is a common target of cyclophilin-cyclosporin A and FKBP-FK506 complexes. Cell. 1991. doi: 10.1016/0092-8674(91)90124-h 1715244

[38] Glowacka P, Rudnicka L, Warszawik-Hendzel O, Sikora M, Goldust M, Gajda P, et al. The antiviral properties of cyclosporine. Focus on coronavirus, hepatitis C virus, influenza virus, and human immunodeficiency virus infections. Biology (Basel). 2020. doi: 10.3390/biology9080192 32731331 PMC7463439

[39] Bose S, Mathur M, Bates P, Joshi N, Banerjee AK. Requirement for cyclophilin A for the replication of vesicular stomatitis virus New Jersey serotype. J Gen Virol. 2003. doi: 10.1099/vir.0.19074-0 12810862

[40] V’kovski P, Kratzel A, Steiner S, Stalder H, Thiel V. Coronavirus biology and replication: implications for SARS-CoV-2. Nat Rev Microbiol. 2020. doi: 10.1038/s41579-020-00468-6 33116300 PMC7592455

[41] V’kovski P, Gerber M, Kelly J, Pfaender S, Ebert N, Braga Lagache S, et al. Determination of host proteins composing the microenvironment of coronavirus replicase complexes by proximity-labeling. Elife. 2019. doi: 10.7554/eLife.42037 30632963 PMC6372286

[42] Tay MZ, Poh CM, Rénia L, MacAry PA, Ng LFP. The trinity of COVID-19: immunity, inflammation and intervention. Nat Rev Immunol. 2020. doi: 10.1038/s41577-020-0311-8 32346093 PMC7187672

[43] Bouhaddou M, Memon D, Meyer B, White KM, Rezelj VV, Correa Marrero M, et al. The Global Phosphorylation Landscape of SARS-CoV-2 Infection. Cell. 2020. doi: 10.1016/j.cell.2020.06.034 32645325 PMC7321036

[44] Stukalov A, Girault V, Grass V, Karayel O, Bergant V, Urban C, et al. Multilevel proteomics reveals host perturbations by SARS-CoV-2 and SARS-CoV. Nature. 2021;594. doi: 10.1038/s41586-021-03493-4 33845483

[45] Wang PG, Tang DJ, Hua Z, Wang Z, An J. Sunitinib reduces the infection of SARS-CoV, MERS-CoV and SARS-CoV-2 partially by inhibiting AP2M1 phosphorylation. Cell Discovery. 2020. doi: 10.1038/s41421-020-00217-2 33083006 PMC7550610

[46] Wang R, Simoneau CR, Kulsuptrakul J, Bouhaddou M, Travisano KA, Hayashi JM, et al. Genetic Screens Identify Host Factors for SARS-CoV-2 and Common Cold Coronaviruses. Cell. 2020. doi: 10.1016/j.cell.2020.12.004 33333024 PMC7723770

[47] Gordon DE, Jang GM, Bouhaddou M, Xu J, Obernier K, White KM, et al. A SARS-CoV-2 protein interaction map reveals targets for drug repurposing. Nature. 2020. doi: 10.1038/s41586-020-2286-9 32353859 PMC7431030

[48] Zang R, Case JB, Yutuc E, Ma X, Shen S, MFG C, et al. Cholesterol 25-hydroxylase suppresses SARS-CoV-2 replication by blocking membrane fusion. Proc Natl Acad Sci U S A. 2020. doi: 10.1073/pnas.2012197117 33239446 PMC7749331

[49] Kools P, Van Imschoot G, Van Roy F. Characterization of three novel human cadherin genes (CDH7, CDH19, and CDH20) clustered on chromosome 18q22-q23 and with high homology to chicken cadherin-7. Genomics. 2000. doi: 10.1006/geno.2000.6305 10995570

[50] Mateo M, Generous A, Sinn PL, Cattaneo R. Connections matter—How viruses use cell-cell adhesion components. J Cell Sci. 2015. doi: 10.1242/jcs.159400 26046138 PMC4311127

[51] Hu Q, Zhang F, Duan L, Wang B, Ye Y, Li P, et al. E-cadherin Plays a Role in Hepatitis B Virus Entry Through Affecting Glycosylated Sodium-Taurocholate Cotransporting Polypeptide Distribution. Front Cell Infect Microbiol. 2020. doi: 10.3389/fcimb.2020.00074 32175289 PMC7056903

[52] Dikic I, Elazar Z. Mechanism and medical implications of mammalian autophagy. Nat Rev Mol Cell Biol. 2018. doi: 10.1038/s41580-018-0003-4 29618831

[53] Wei J, Alfajaro MM, PC DW, Hanna RE, Lu-Culligan WJ, Cai WL, et al. Genome-wide CRISPR Screens Reveal Host Factors Critical for SARS-CoV-2 Infection. Cell. 2021. doi: 10.1016/j.cell.2020.10.028 33147444 PMC7574718

[54] Baggen J, Persoons L, Vanstreels E, Jansen S, Van Looveren D, Boeckx B, et al. Genome-wide CRISPR screening identifies TMEM106B as a proviral host factor for SARS-CoV-2. Nat Genet. 2021. doi: 10.1038/s41588-021-00805-2 33686287

[55] Molejon MI, Ropolo A, Re A, Lo, Boggio V, Vaccaro MI. The VMP1-Beclin 1 interaction regulates autophagy induction. Sci Rep. 2013. doi: 10.1038/srep01055 23316280 PMC3542764

[56] Bai X, Ma D, Liu A, Shen X, Wang QJ, Liu Y, et al. Rheb activates mTOR by antagonizing its endogenous inhibitor, FKBP38. Science. 2007. doi: 10.1126/science.1147379 17991864

[57] Saita S, Shirane M, Nakayama KI. Selective escape of proteins from the mitochondria during mitophagy. Nat Commun. 2013. doi: 10.1038/ncomms2400 23361001

[58] Medina DL, Di Paola S, Peluso I, Armani A, De Stefani D, Venditti R, et al. Lysosomal calcium signalling regulates autophagy through calcineurin and TFEB. Nat Cell Biol. 2015. doi: 10.1038/ncb3114 25720963 PMC4801004

[59] Reggiori F, Monastyrska I, Verheije MH, Calì T, Ulasli M, Bianchi S, et al. Coronaviruses hijack the LC3-I-positive EDEMosomes, ER-derived vesicles exporting short-lived ERAD regulators, for replication. Cell Host Microbe. 2010. doi: 10.1016/j.chom.2010.05.013 20542253 PMC7103375

[60] Gosert R, Kanjanahaluethai A, Egger D, Bienz K, Baker SC. RNA Replication of Mouse Hepatitis Virus Takes Place at Double-Membrane Vesicles. J Virol. 2002. doi: 10.1128/jvi.76.8.3697–3708.2002 PMC136101 11907209

[61] Prentice E, Jerome WG, Yoshimori T, Mizushima N, Denison MR. Coronavirus Replication Complex Formation Utilizes Components of Cellular Autophagy. J Biol Chem. 2004. doi: 10.1074/jbc.M306124200 14699140 PMC7957857

[62] Zhao Z, Thackray LB, Miller BC, Lynn TM, Becker MM, Ward E, et al. Coronavirus replication does not require the autophagy gene ATG5. Autophagy. 2007. doi: 10.4161/auto.4782 17700057

[63] Prentice E, McAuliffe J, Lu X, Subbarao K, Denison MR. Identification and Characterization of Severe Acute Respiratory Syndrome Coronavirus Replicase Proteins. J Virol. 2004. doi: 10.1128/JVI.78.18.9977-9986.2004 15331731 PMC514967

[64] Snijder EJ, van der Meer Y, Zevenhoven-Dobbe J, JJM O, van der Meulen J, Koerten HK, et al. Ultrastructure and Origin of Membrane Vesicles Associated with the Severe Acute Respiratory Syndrome Coronavirus Replication Complex. J Virol. 2006. doi: 10.1128/JVI.02501-05 16731931 PMC1472606

[65] Monastyrska I, Ulasli M, PJM R, Guan JL, Reggiori F, CAM DH, et al. An autophagy-independent role for LC3 in equine arteritis virus replication. Autophagy. 2013. doi: 10.4161/auto.22743 23182945 PMC3552881

[66] Bhujabal Z. FKBP8 and the autophagy-inducing Class-III PI3K Complex Roles of LIR dependent interactions. 2017.

[67] Gassen NC, Niemeyer D, Muth D, Corman VM, Martinelli S, Gassen A, et al. SKP2 attenuates autophagy through Beclin1-ubiquitination and its inhibition reduces MERS-Coronavirus infection. Nat Commun. 2019. doi: 10.1038/s41467-019-13659-4 31852899 PMC6920372

[68] Hoffmann H-H, Schneider WM, Rozen-Gagnon K, Miles LA, Schuster F, Razooky B, et al. TMEM41B is a pan-flavivirus host factor. bioRxiv. 2020. doi: 10.1101/2020.10.09.334128 33338421 PMC7954666

[69] Schneider WM, Luna JM, Hoffmann H-H, Sánchez-Rivera FJ, Leal AA, et al. Genome-scale identification of SARS-CoV-2 and pan-coronavirus host factor networks. bioRxiv. 2020. doi: 10.1101/2020.10.07.326462 33382968 PMC7796900

[70] Xu SS, Xu LG, Yuan C, Li SN, Chen T, Wang W, et al. FKBP8 inhibits virus-induced RLR-VISA signaling. J Med Virol. 2019. doi: 10.1002/jmv.25327 30267576

[71] Pfefferle S, Schöpf J, Kögl M, Friedel CC, Müller MA, Carbajo-Lozoya J, et al. The SARS-Coronavirus-host interactome: Identification of cyclophilins as target for pan-Coronavirus inhibitors. PLoS Pathog. 2011. doi: 10.1371/journal.ppat.1002331 22046132 PMC3203193

[72] Carbajo-Lozoya J, Ma-Lauer Y, Malešević M, Theuerkorn M, Kahlert V, Prell E, et al. Human coronavirus NL63 replication is cyclophilin A-dependent and inhibited by non-immunosuppressive cyclosporine A-derivatives including Alisporivir. Virus Res. 2014. doi: 10.1016/j.virusres.2014.02.010 24566223 PMC7114444

[73] Carbajo-Lozoya J, Müller MA, Kallies S, Thiel V, Drosten C, Von Brunn A. Replication of human coronaviruses SARS-CoV, HCoV-NL63 and HCoV-229E is inhibited by the drug FK506. Virus Res. 2012. doi: 10.1016/j.virusres.2012.02.002 22349148 PMC7114512

[74] de Wilde AH, Zevenhoven-Dobbe JC, van der Meer Y, Thiel V, Narayanan K, Makino S, et al. Cyclosporin A inhibits the replication of diverse coronaviruses. J Gen Virol. 2011. doi: 10.1099/vir.0.034983-0 21752960 PMC3352363

[75] de Wilde AH, Falzarano D, Zevenhoven-Dobbe JC, Beugeling C, Fett C, Martellaro C, et al. Alisporivir inhibits MERS- and SARS-coronavirus replication in cell culture, but not SARS-coronavirus infection in a mouse model. Virus Res. 2017. doi: 10.1016/j.virusres.2016.11.011 27840112 PMC7114565

[76] de Wilde AH, Pham U, Posthuma CC, Snijder EJ. Cyclophilins and cyclophilin inhibitors in nidovirus replication. Virology. 2018. doi: 10.1016/j.virol.2018.06.011 30014857 PMC7112023

[77] Softic L, Brillet R, Berry F, Ahnou N, Nevers Q, Morin-Dewaele M, et al. Inhibition of SARS-CoV-2 infection by the cyclophilin inhibitor alisporivir (Debio 025). Antimicrob Agents Chemother. 2020. doi: 10.1128/AAC.00876-20 32376613 PMC7318051

[78] Thiel V, Siddell SG. Reverse genetics of coronaviruses using vaccinia virus vectors. Curr Top Microbiol Immunol. 2005. doi: 10.1007/3-540-26765-4_7 15609513 PMC8387696

[79] Kindler E, Jónsdóttir HR, Muth D, Hamming OJ, Hartmann R, Rodriguez R, et al. Efficient replication of the novel human betacoronavirus EMC on primary human epithelium highlights its zoonotic potential. mBio. 2013. doi: 10.1128/mBio.00611-12 23422412 PMC3573664

[80] Thiel V, Ivanov KA, Putics Á, Hertzig T, Schelle B, Bayer S, et al. Mechanisms and enzymes involved in SARS coronavirus genome expression. J Gen Virol. 2003. doi: 10.1099/vir.0.19424-0 12917450

[81] Hanika A, Larisch B, Steinmann E, Schwegmann-Weßels C, Herrler G, Zimmer G. Use of influenza C virus glycoprotein HEF for generation of vesicular stomatitis virus pseudotypes. J Gen Virol. 2005. doi: 10.1099/vir.0.80788-0 15831958

[82] Irwin DM, Kocher TD, Wilson AC. Evolution of the cytochrome b gene of mammals. J Mol Evol. 1991. doi: 10.1007/BF02515385 1901092

[83] Shalem O, Sanjana NE, Hartenian E, Shi X, Scott DA, Mikkelsen TS, et al. Genome-scale CRISPR-Cas9 knockout screening in human cells. Science. 2014. doi: 10.1126/science.1247005 24336571 PMC4089965

[84] Illumina. Illumina adapter sequences. Illumina. 2009.

[85] Shrestha N et al. Antiviral screen against canine distemper virus-induced membrane fusion activity. Viruses. 2021. doi: 10.3390/v13010128 33477492 PMC7831055

[86] Places G, Hierholzer JC, Killington RA. Cell Culture Cell Culture. Virol Methods Manual. 1996;76:2–6.

[87] Corman VM, Eckerle I, Bleicker T, Zaki A, Landt O, Eschbach-Bludau M, et al. Detection of a novel human coronavirus by real-time reverse-transcription polymerase chain reaction. Eur Secur. 2012. doi: 10.2807/ese.17.39.20285-en 23041020

[88] Corman VM, Landt O, Kaiser M, Molenkamp R, Meijer A, DKW C, et al. Detection of 2019 novel coronavirus (2019-nCoV) by real-time RT-PCR. Eur Secur. 2020;25:2000045. 10.2807/1560-7917.ES.2020.25.3.2000045 PMC6988269 31992387

[89] Thao TTN, Labroussaa F, Ebert N, V’kovski P, Stalder H, Portmann J, et al. Rapid reconstruction of SARS-CoV-2 using a synthetic genomics platform. Nature. 2020. doi: 10.1038/s41586-020-2294-9 32365353

[90] Schindelin J, Arganda-Carreras I, Frise E, Kaynig V, Longair M, Pietzsch T, et al. Fiji: An open-source platform for biological-image analysis. Nat Methods. 2012. doi: 10.1038/nmeth.2019 22743772 PMC3855844

[91] Mutterer J, Zinck E. Quick-and-clean article figures with FigureJ. J Microsc. 2013. doi: 10.1111/jmi.12069 23906423

[92] Stringer C, Michaelos M, Pachitariu M. Cellpose: A generalist algorithm for cellular segmentation. bioRxiv. 2020. doi: 10.1038/s41592-020-01018-x 33318659

