# Supplementary figures and images for: A genome-wide CRISPR screen identifies interactors of the autophagy pathway as conserved coronavirus targets
Source: PLoS Biol. 2021 Dec 28;19(12):e3001490. doi: 10.1371/journal.pbio.3001490 (PMC8741300; doi:10.1371/journal.pbio.3001490)

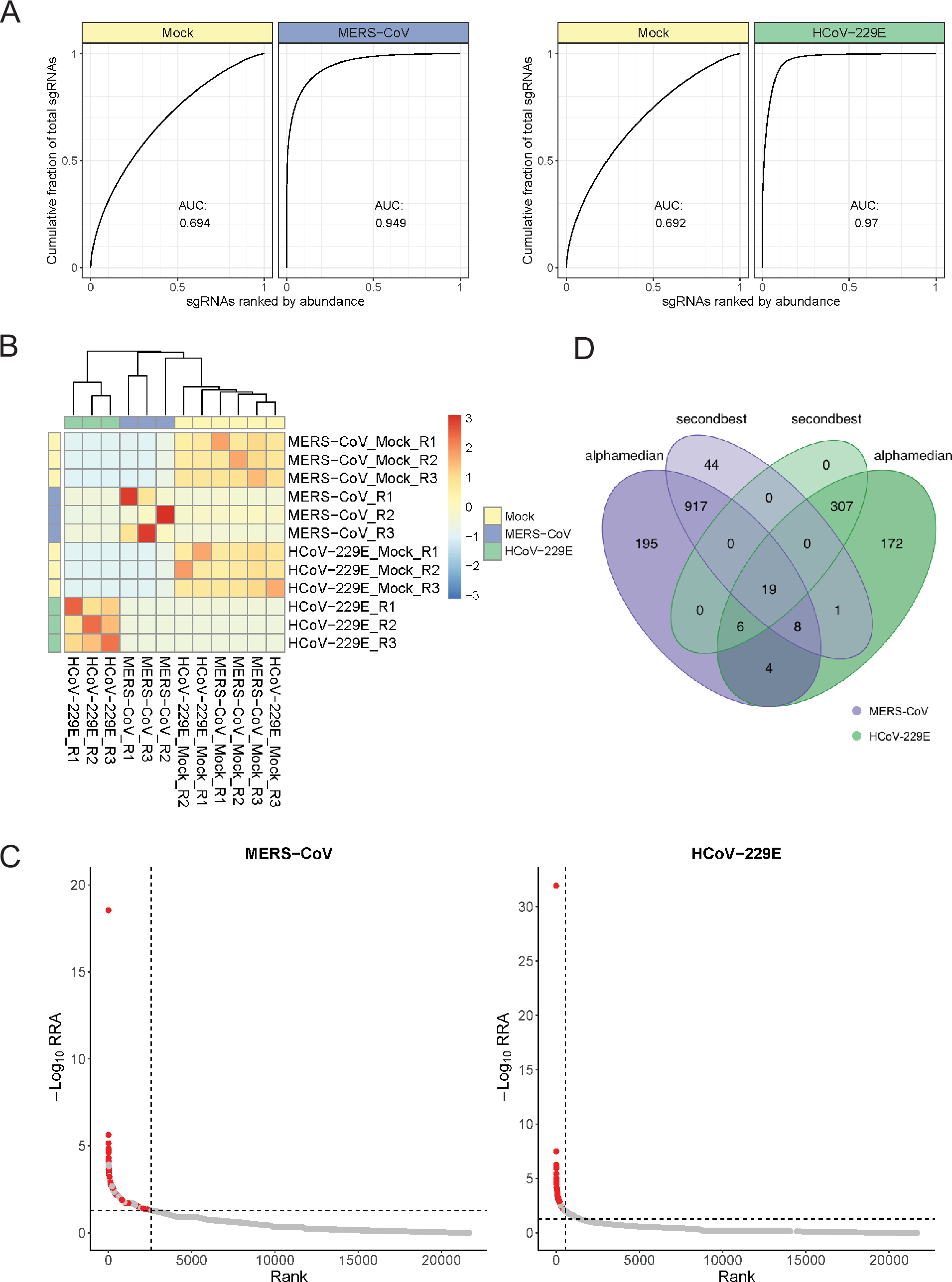

Supplement: S1 Fig — (A) AUC analysis of MERS-CoV and HCoV-229E CRISPR screens evaluating sgRNA library representation in surviving Huh7 cells from uninfected (Mock) and MERS-CoV (left 2 panels) or HCoV-229E (right 2 panels) infected samples. For each CRISPR screen, sgRNA abundance was calculated based on average sgRNA abundance over 3 independent biological replicates. Raw data for calculations can be found in Supporting information S1 Data, tabs 3 and 4. (B) Correlation matrix depicting the Pearson correlation for guide-level normalized read counts among biological replicates and samples from both screens. R1, R2, and R3 represent the biological replicates 1, 2, and 3, respectively. Clustering was performed in pheatmap using correlation as a distance metric. Raw data can be found in Supporting information S1 Data, tab 5. (C) RRA p-value distribution of all genes in the GeCKOv2 library for both MERS-CoV (left) and HCoV-229E (right) CRISPR screens. Genes that met the criteria for significance (RRA p-value ≤0.05 and FC ≥ 2) are highlighted in red. Raw data can be found in Supporting information S1 Data, tab 1. (D) Venn diagram illustrating the overlap between significantly enriched genes from both CRISPR screens that were identified via 2 different RRA-based analysis methods (alpha median and second best). A total of 19 genes were identified by both methods in both MERS-CoV and HCoV-229E CRISPR screens. Raw data can be found in Supporting information S1 Data, tab 1. AUC, area under the curve; HCoV, human coronavirus; MERS-CoV, Middle East Respiratory Syndrome Coronavirus; RRA, robust rank aggregation; sgRNA, single guide RNA. (TIF) [file pbio.3001490.s001.tif]

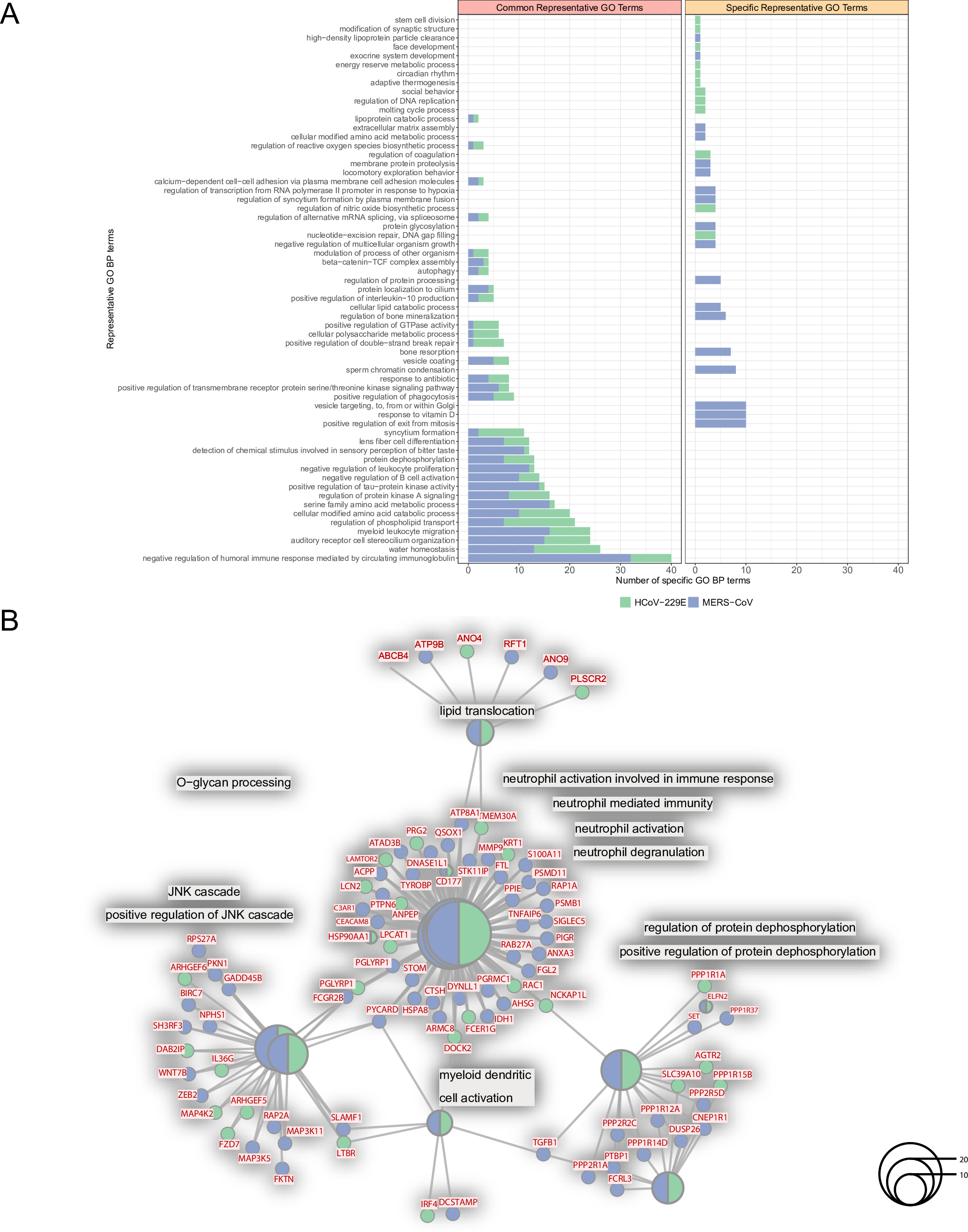

Supplement: S2 Fig — (A) Representative GO terms identified using full list of enriched GO terms for MERS-CoV and HCoV-229E screens (S2 Table). Representative terms found in both screens are shown in the top panel, whereas virus-specific terms are shown in the bottom panel. BP, CC, and MF represent different GO term categories. (B) Specific GO terms enriched in both CoV screens (individual GO terms, not representative GO terms). Raw data can be found in S2 Table. CoV, coronavirus; GO, Gene Ontology; HCoV, human coronavirus; MERS-CoV, Middle East Respiratory Syndrome Coronavirus. (TIF) [file pbio.3001490.s002.tif]

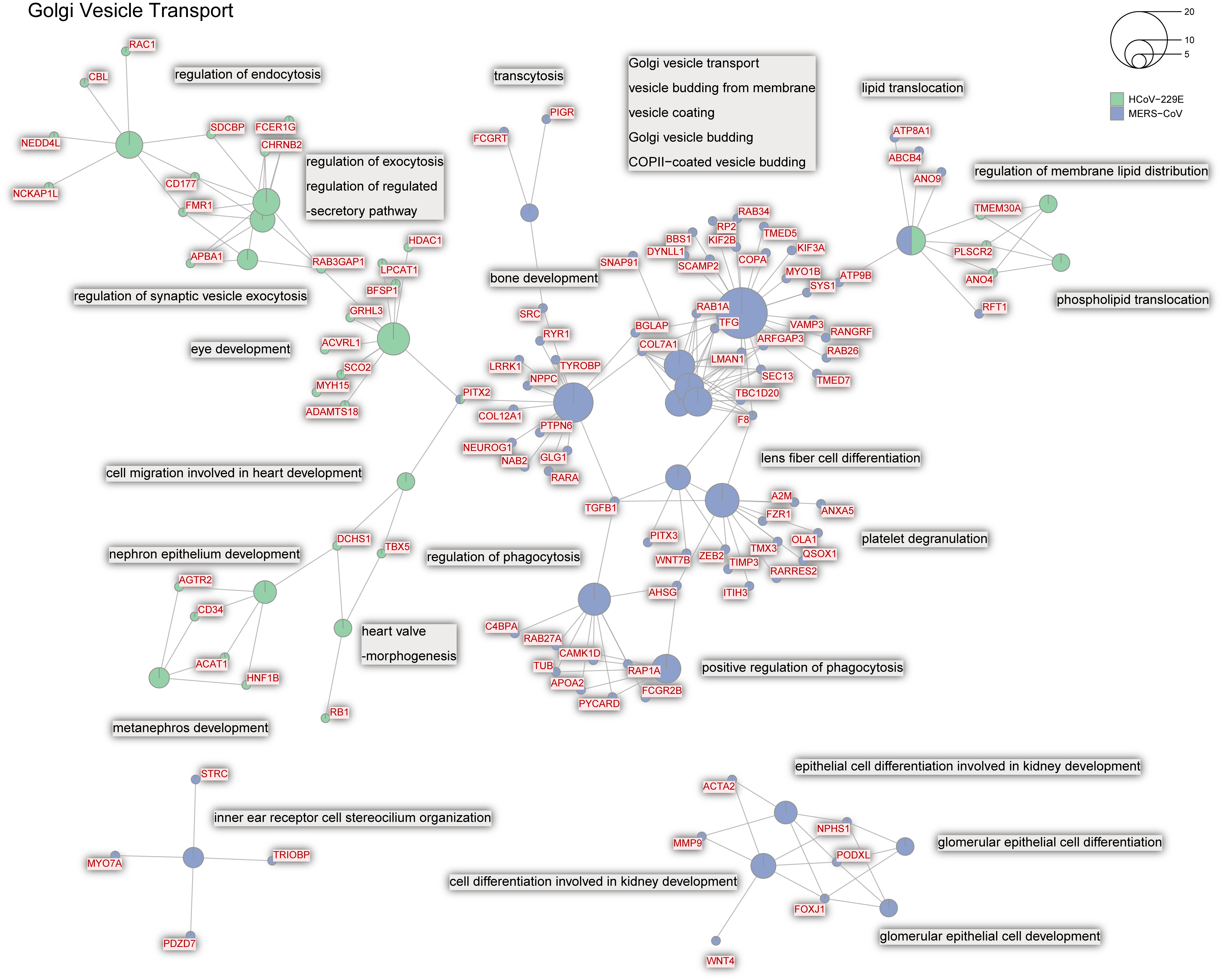

Supplement: S3 Fig — Cnet plot for the Golgi vesicle transport cluster shown in Fig 2A. The plot includes both GO terms that contain 1 or more of the 19 common significantly enriched genes found in both CoV screens (as in Fig 2A and 2B) as well as representative GO terms found in both screens that do not contain these genes. The plot shows the relationship among individual GO terms and genes found in the Golgi vesicle transport cluster. Larger nodes represent individual GO terms, and smaller nodes represent individual gene. Nodes that are functionally related cluster together into a larger network. Node size reflects the number of significantly enriched genes in the node, and color indicates the CoV screen for which the node was significant. Raw data can be found in S2 Table. CoV, coronavirus; GO, Gene Ontology; HCoV, human coronavirus; MERS-CoV, Middle East Respiratory Syndrome Coronavirus. (TIFF) [file pbio.3001490.s003.tiff]

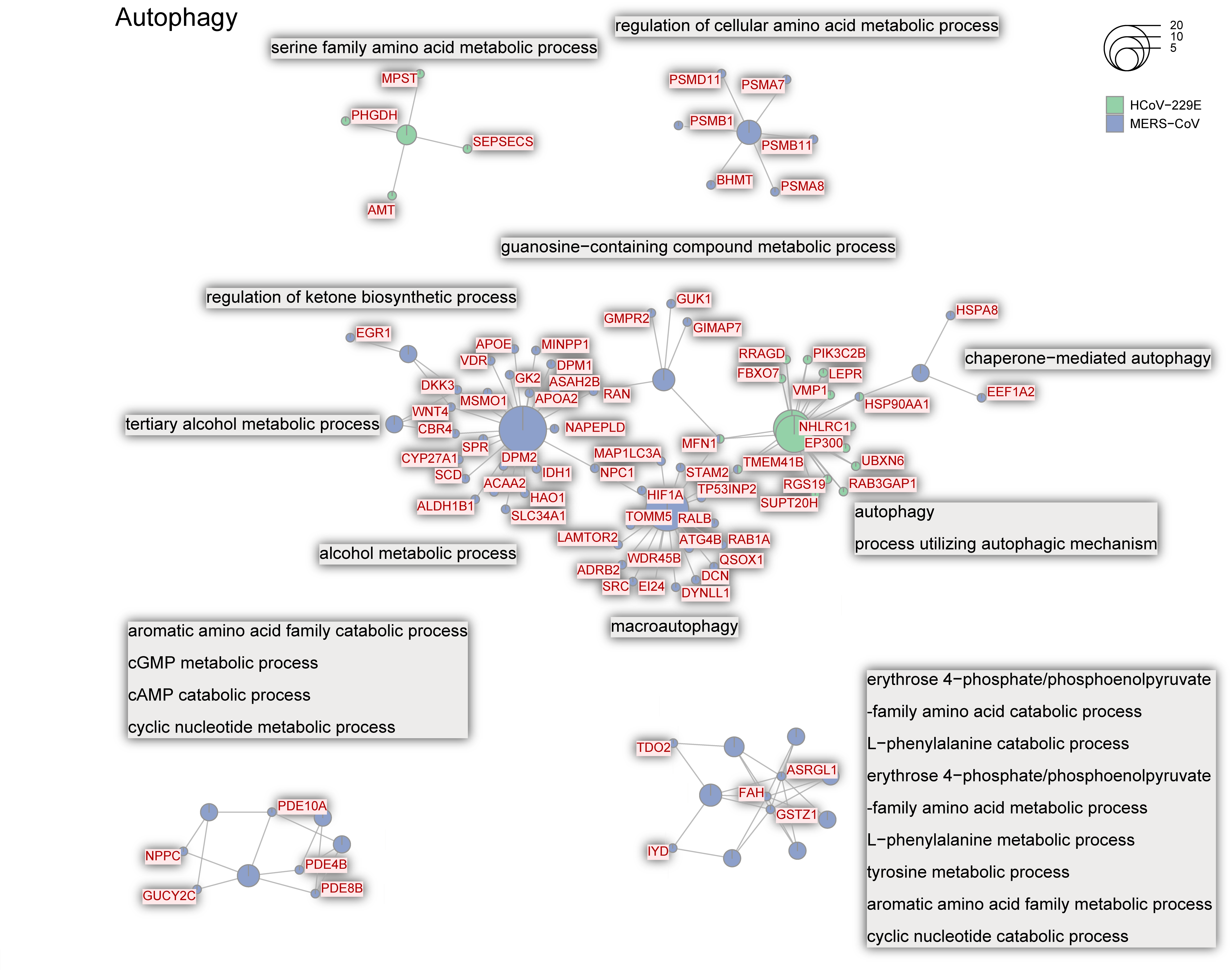

Supplement: S4 Fig — Cnet plot for the autophagy cluster shown in Fig 2A. The plot includes both GO terms that contain 1 or more of the 19 common significantly enriched genes found in both CoV screens (as in Fig 2A and 2B) as well as representative GO terms found in both screens that do not contain these genes. Each plot shows the relationship among individual GO terms and genes found in each biological cluster. Larger nodes represent individual GO terms, and smaller nodes represent individual gene. Nodes that are functionally related cluster together into a larger network. Node size reflects the number of significantly enriched genes in the node, and color indicates the CoV screen for which the node was significant. Raw data can be found in S2 Table. CoV, coronavirus; GO, Gene Ontology; HCoV, human coronavirus; MERS-CoV, Middle East Respiratory Syndrome Coronavirus. (TIF) [file pbio.3001490.s004.tif]

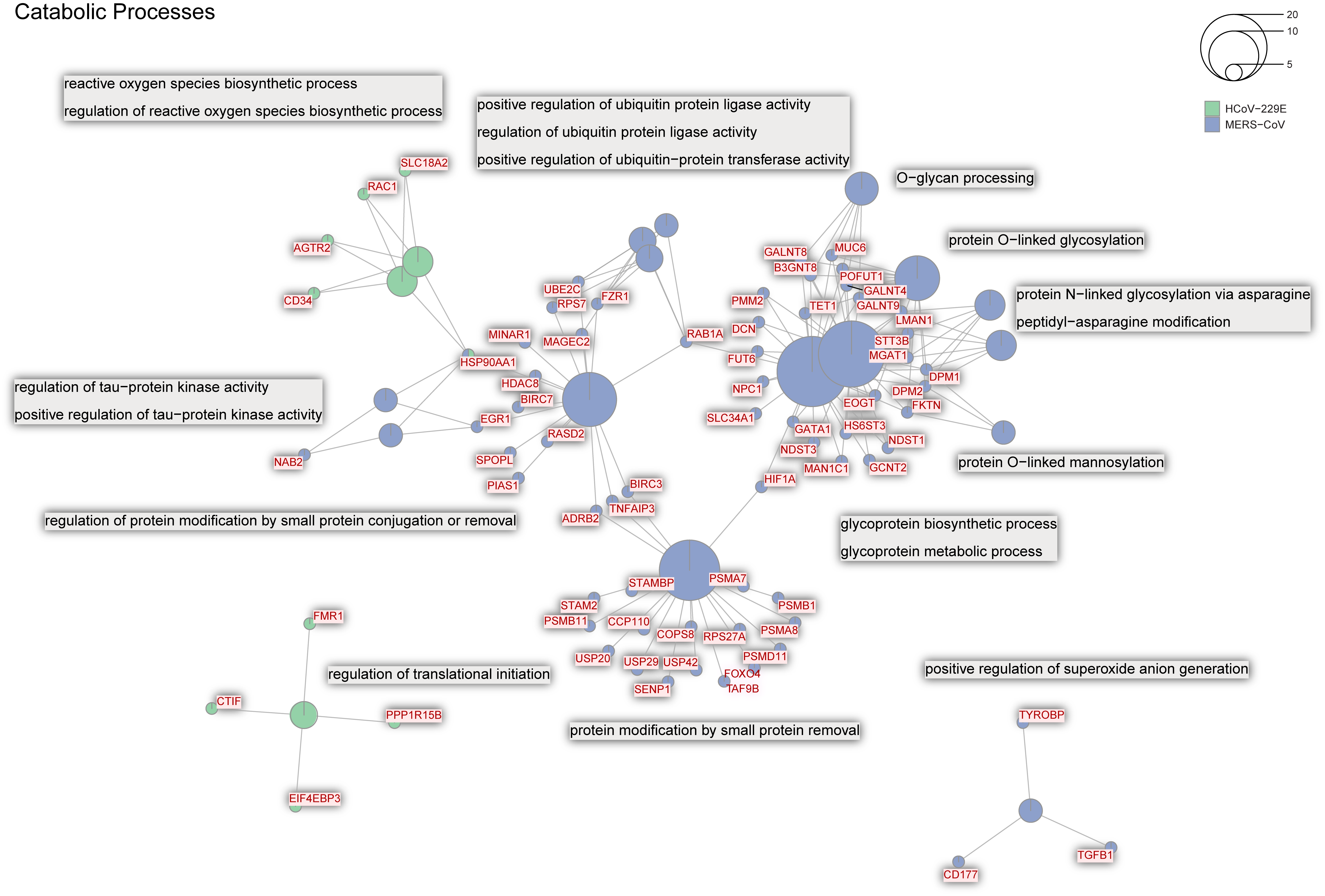

Supplement: S5 Fig — Cnet plot for the catabolic processes cluster shown in Fig 2A. Plots include both GO terms that contain 1 or more of the 19 common significantly enriched genes found in both CoV screens (as in Fig 2A and 2B) as well as representative GO terms found in both screens that do not contain these genes. The plot shows the relationship among individual GO terms and genes found in the catabolic processes cluster. Larger nodes represent individual GO terms, and smaller nodes represent individual gene. Nodes that are functionally related cluster together into a larger network. Node size reflects the number of significantly enriched genes in the node, and color indicates the CoV screen for which the node was significant. Raw data can be found in S2 Table. CoV, coronavirus; GO, Gene Ontology; HCoV, human coronavirus; MERS-CoV, Middle East Respiratory Syndrome Coronavirus. (TIF) [file pbio.3001490.s005.tif]

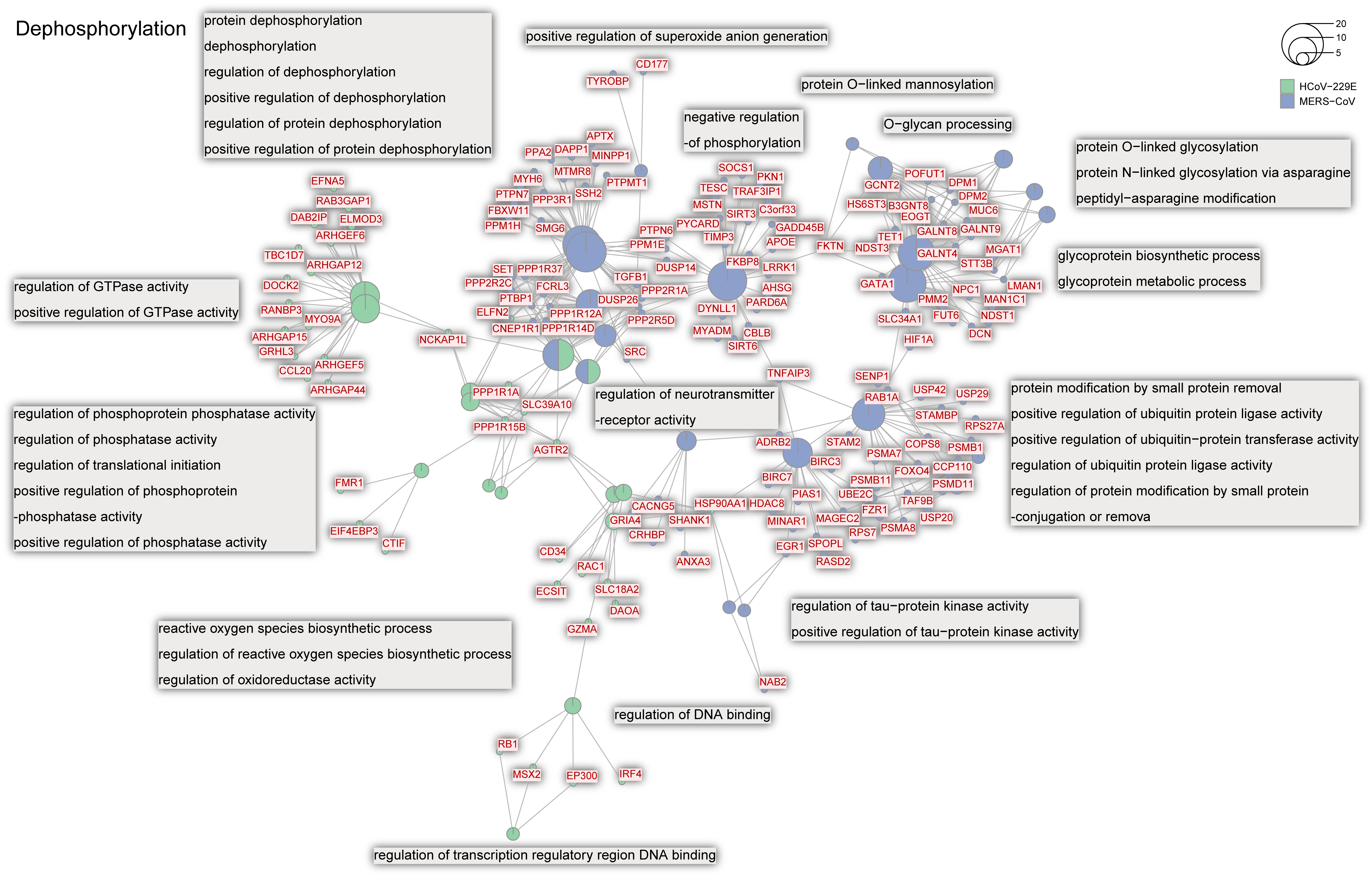

Supplement: S6 Fig — Cnet plot for the dephosphorylation cluster shown in Fig 2A. The plot includes both GO terms that contain 1 or more of the 19 common significantly enriched genes found in both CoV screens (as in Fig 2A and 2B) as well as representative GO terms found in both screens that do not contain these genes. The plot shows the relationship among individual GO terms and genes found in the dephosphorylation cluster. Larger nodes represent individual GO terms, and smaller nodes represent individual gene. Nodes that are functionally related cluster together into a larger network. Node size reflects the number of significantly enriched genes in the node, and color indicates the CoV screen for which the node was significant. Raw data can be found in S2 Table. CoV, coronavirus; GO, Gene Ontology; HCoV, human coronavirus; MERS-CoV, Middle East Respiratory Syndrome Coronavirus. (TIFF) [file pbio.3001490.s006.tiff]

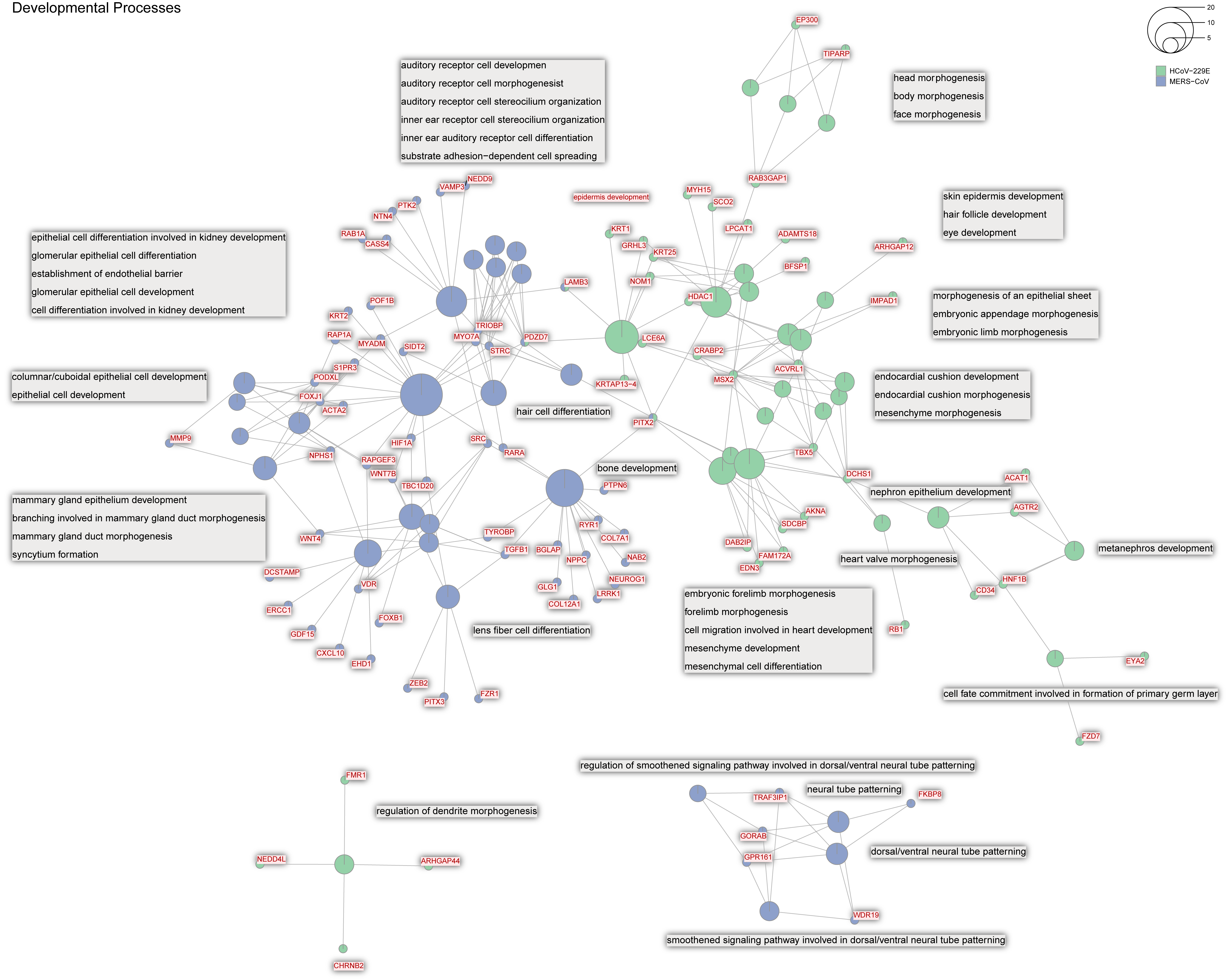

Supplement: S8 Fig — Cnet plot for the developmental processes cluster shown in Fig 2A. The plot includes both GO terms that contain 1 or more of the 19 common significantly enriched genes found in both CoV screens (as in Fig 2A and 2B) as well as representative GO terms found in both screens that do not contain these genes. The plot shows the relationship among individual GO terms and genes found in the developmental processes cluster. Larger nodes represent individual GO terms, and smaller nodes represent individual gene. Nodes that are functionally related cluster together into a larger network. Node size reflects the number of significantly enriched genes in the node, and color indicates the CoV screen for which the node was significant. Raw data can be found in S2 Table. CoV, coronavirus; GO, Gene Ontology; HCoV, human coronavirus; MERS-CoV, Middle East Respiratory Syndrome Coronavirus. (TIFF) [file pbio.3001490.s008.tiff]

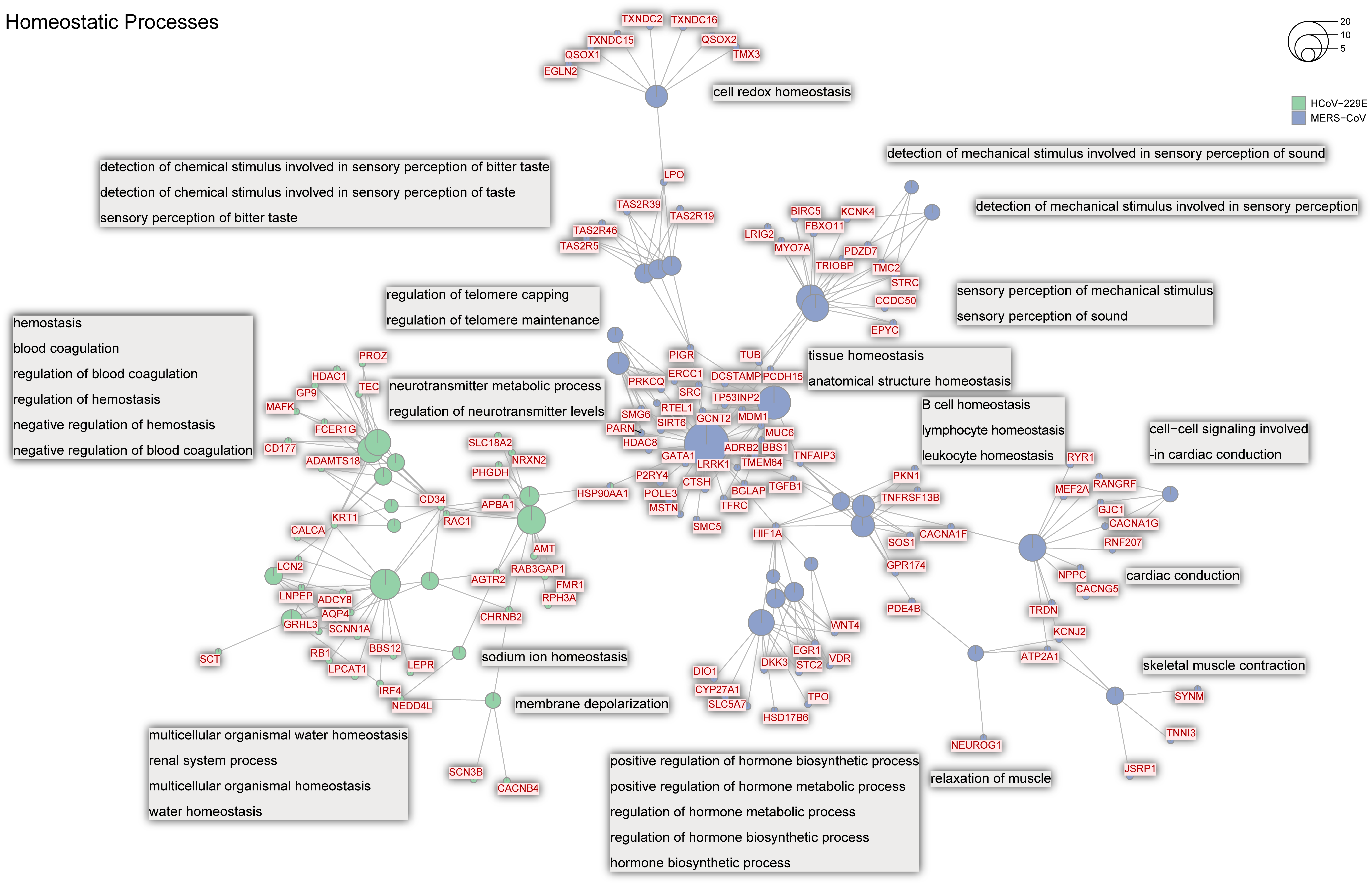

Supplement: S9 Fig — Cnet plot for the homeostatic processes cluster shown in Fig 2A. The plot includes both GO terms that contain 1 or more of the 19 common significantly enriched genes found in both CoV screens (as in Fig 2A and 2B) as well as representative GO terms found in both screens that do not contain these genes. The plot shows the relationship among individual GO terms and genes found in the homeostatic processes cluster. Larger nodes represent individual GO terms, and smaller nodes represent individual gene. Nodes that are functionally related cluster together into a larger network. Node size reflects the number of significantly enriched genes in the node, and color indicates the CoV screen for which the node was significant. Raw data can be found in S2 Table. CoV, coronavirus; GO, Gene Ontology; HCoV, human coronavirus; MERS-CoV, Middle East Respiratory Syndrome Coronavirus. (TIFF) [file pbio.3001490.s009.tiff]

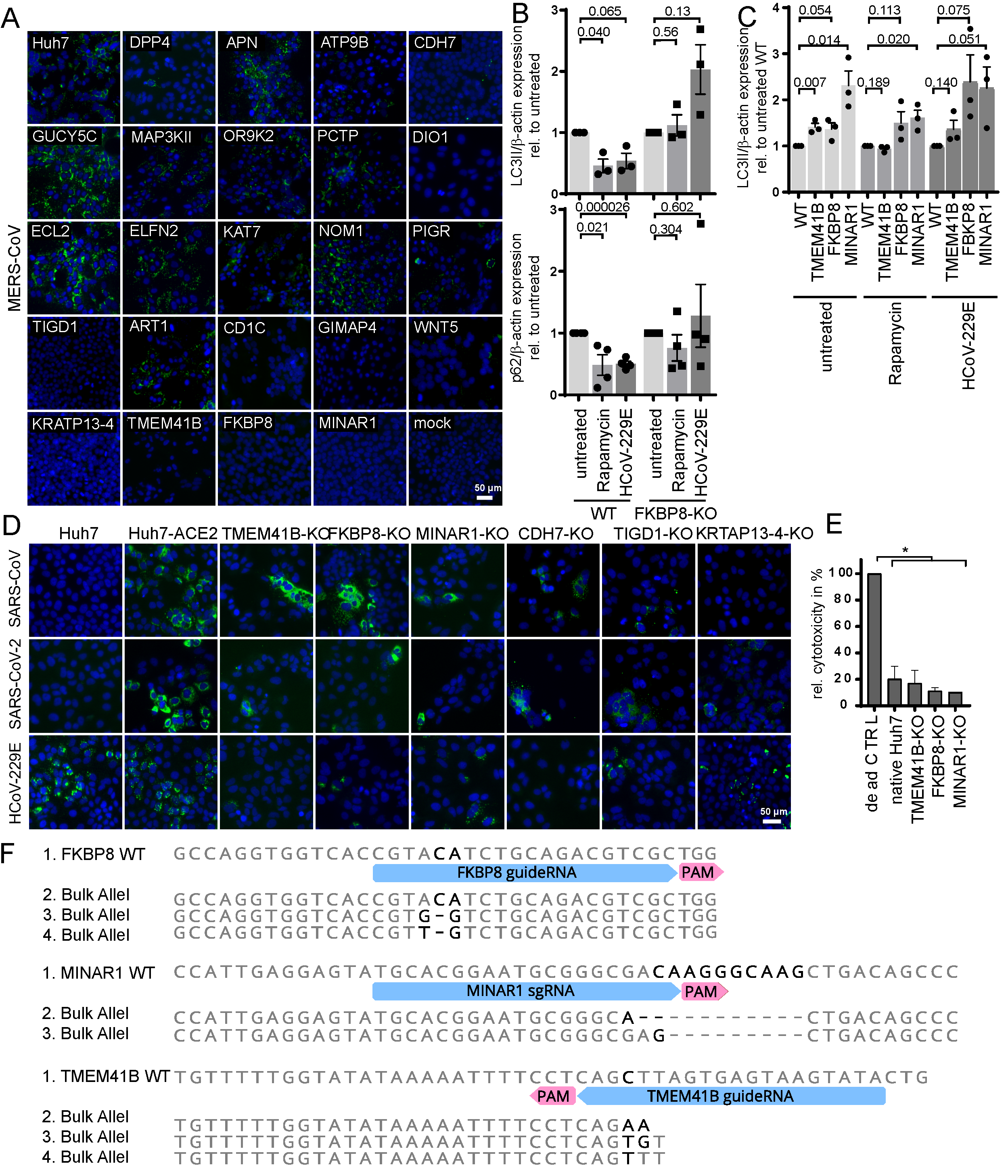

Supplement: S10 Fig — (A) Immunofluorescence staining of MERS-CoV infected of Huh7 cells containing KO of top scoring HDFs. dsRNA is shown in green, and DAPI is shown in blue. (B) Western blot analysis quantification of LC3II and p62 expression. Protein expression is normalized to beta actin and relative to the untreated condition in WT and FKBP8-KO cell treated with rapamycin or infected with HCoV-229E. Quantification data can be found in Supporting information S1 Data, tab 15. Western blot raw images are depicted in S1 Raw Images. (C) Western blot analysis quantification of LC3II expression normalized to beta actin and relative to WT. Protein expression is depicted in TMEM41B-, MINAR1-, and FKBP8-KO cells after treatment with rapamycin and HCoV-229E infection. Statistical significance was determined using the Holm–Sidak method in GraphPad Prism. p-Values are indication, and p-values <0.05 are defined as statistically significant. Quantification data can be found in Supporting information S1 Data, tab 16. Western blot raw images are shown in S1 Raw Images. (D) Immunofluorescence staining of HCoV-229E, SARS-CoV, and SARS-CoV-2 infected Huh7 cells with TMEM41B, FKBP8, and MINAR1-KO, as well as a stable ACE2 expression. dsRNA is shown in green, DAPI is shown in blue, and ACE2 is shown in red. Scale bar is 50 μm. All images were acquired using an Evos Auto FL2 and processed in Fiji. (E) Relative cytotoxicity of TMEM41-KO, FKBP8-KO, and MINAR1-KO is depicted in %. Two-tailed unpaired Student t test was used to determine significance in GraphPad Prism 8.3.1. (F) Sanger sequencing of FKBP8-KO, MINAR1-KO, and TMEM41B-KO verifies Cas-9–mediated double-strand break in multiple alleles of the KO cells. PAM sequence is indicated in red, and binding site of sgRNA is indicated in blue. Used reagents are listed in detail in Table 1. ACE2, angiotensin converting enzyme 2; CoV, coronavirus; FKBP8, FK506 binding protein 8; HCoV, human coronavirus; HDF, host dependency factor; KO, knockout; MERS-CoV, Midd [file pbio.3001490.s010.tiff]

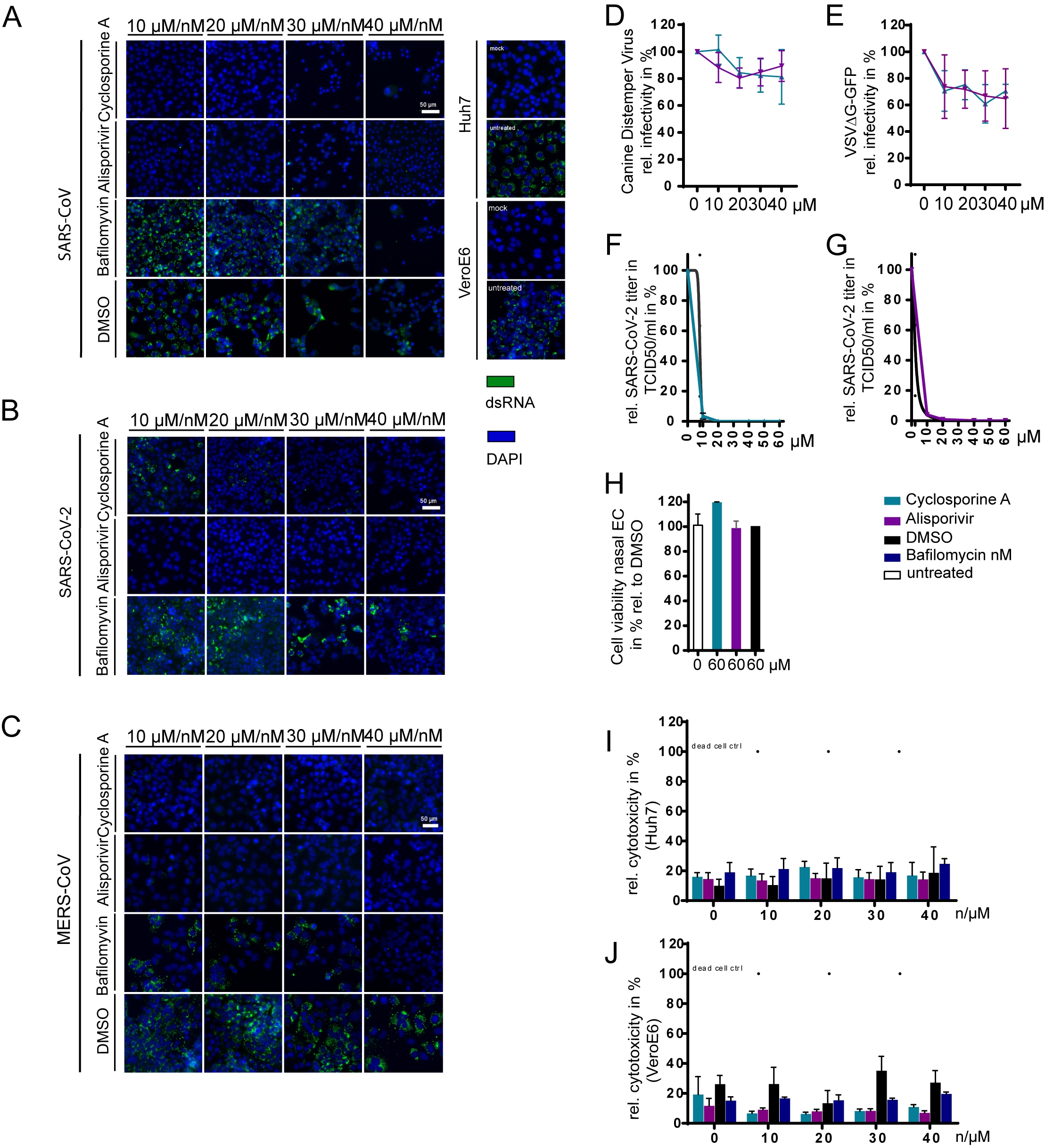

Supplement: S11 Fig — Immunofluorescence staining of SARS-CoV (A), as well as SARS-CoV-2 (B) infected VeroE6 cells and MERS-CoV (C) infected Huh7 cells following cyclosporine A and alisporivir treatment at 10 μM to 40 μM and bafilomycin A1 treatment at 10 nM to 40 nM, as well as DMSO CTRL at respective volumes 24 hours postinfection/inhibitor treatment. dsRNA is shown in green, and DAPI is shown in blue. Scale bar is 50 μm. All images were acquired using an EVOS FL Auto 2 imaging system with a 10× air objective. (D) Relative infectivity of CDV (raw data can be found in Supporting information S1 Data, tab 17) and (E) VSVΔG in % after treatment with cyclosporine A (teal) and alisporivir (purple) (raw data can be found in Supporting information S1 Data, tab 18). (F, G) Inhibitor treated primary nasal epithelial cell cultures displayed as inhibitor versus normalized response. IC50 value is marked with dotted line and indicated on y-axis. Calculations were performed in GraphPad Prism 8.3.1. Raw data can be found in Supporting information S1 Data, tab 19. (H) Relative nasal epithelial cell culture viability upon treatment of 60 μM cyclosporine A and alisporivir normalized to DMSO. Raw data can be found in Supporting information S1 Data, tab 20. Cyclosporine A, alisporivir, and bafilomycin A1, treatment-mediated cytotoxicity in Huh7 cells (J), and VeroE6 cells (K) shown relative to dead cell control. Raw data can be found in Supporting information S1 Data, tabs 21 and 22. Used reagents are listed in detail in Table 1. MERS-CoV, Middle East Respiratory Syndrome Coronavirus; SARS-CoV, Severe Acute Respiratory Syndrome Coronavirus; SARS-CoV-2, Severe Acute Respiratory Syndrome Coronavirus 2. (TIF) [file pbio.3001490.s011.tif]
