# Supplementary material for: A genome-wide CRISPR screen identifies interactors of the autophagy pathway as conserved coronavirus targets
Source: PLoS Biol. 2021 Dec 28;19(12):e3001490. doi: 10.1371/journal.pbio.3001490 (PMC8741300; doi:10.1371/journal.pbio.3001490)
Supplement: S1 Raw Images — Page 1 (upper panel): Western blots for Fig 3G, TMEM41B. (Middle panel) Western blots for Fig 3G, FKBP8. (Lower panel) Western blots for Fig 3G, MINAR1. Page 2: Western blots for S10B Fig graph 1. Page 3: Western blots for S10B Fig graph 2. Page 4: Western blots for S10C Fig, rapamycin treatment. Page 5: Western blots for S10C Fig, HCoV-229E infection. FKBP8, FK506 binding protein 8; HCoV, human coronavirus; MINAR1, Membrane Integral NOTCH2 Associated Receptor 1; TMEM41B, transmembrane protein 41B. (PDF) [file pbio.3001490.s015.pdf]

Figure 3G TMEM41B

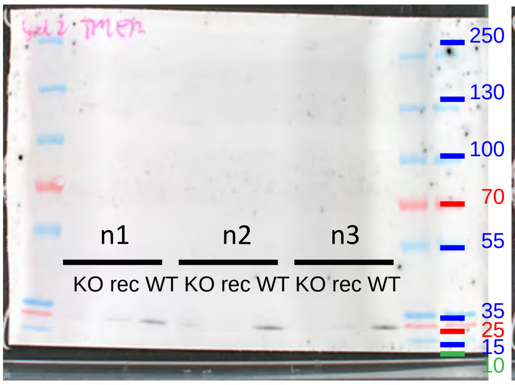

TMEM41B (22kDa), 5 sec

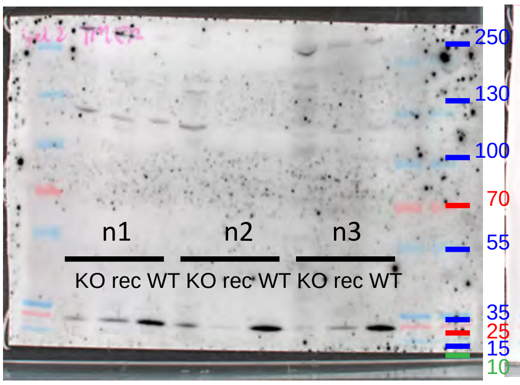

TMEM41B (22kDa), 2 min

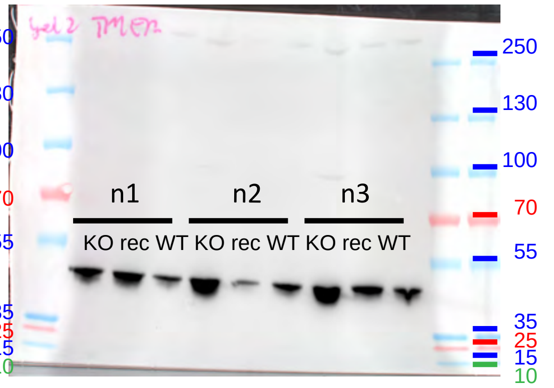

beta actin (42kDa)

Figure 3G FKBP8

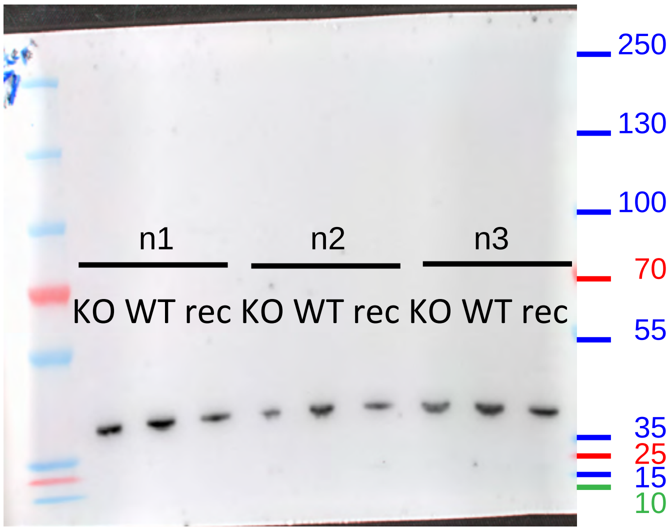

anti-FKBP8 (38kDa)

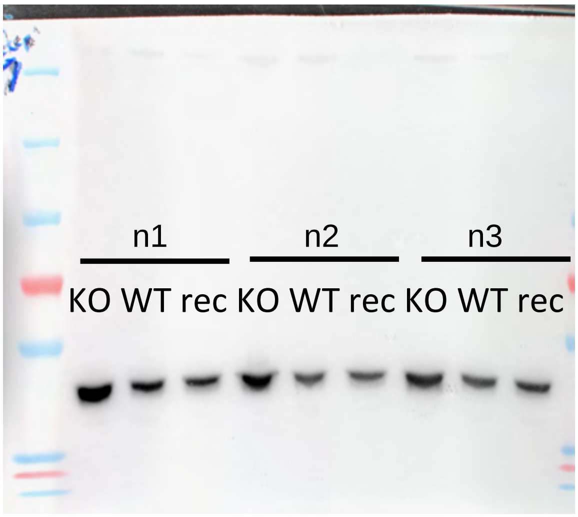

anti beta actin (42kDa)

Figure 3G MINAR1-GFP

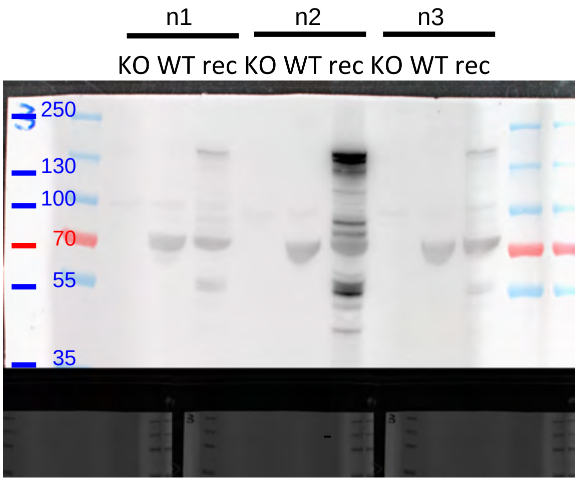

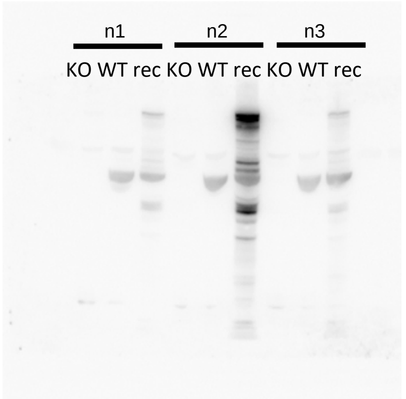

anti-GFP (MINAR1-GFP 130kDa)

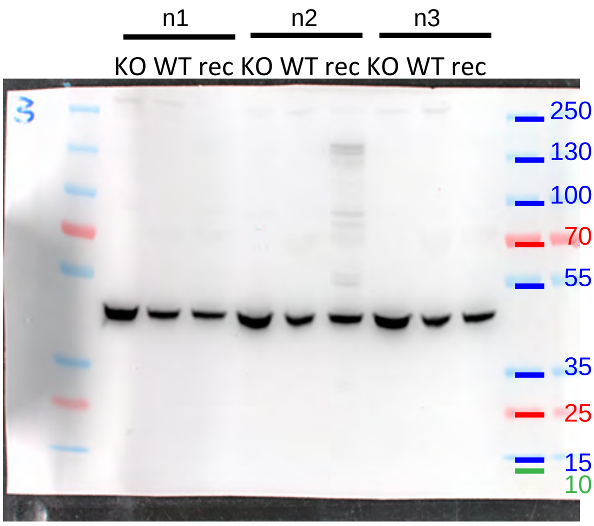

anti-beta actin (42kDa)

Western Blots Figure S10

Figure S10B graph 1

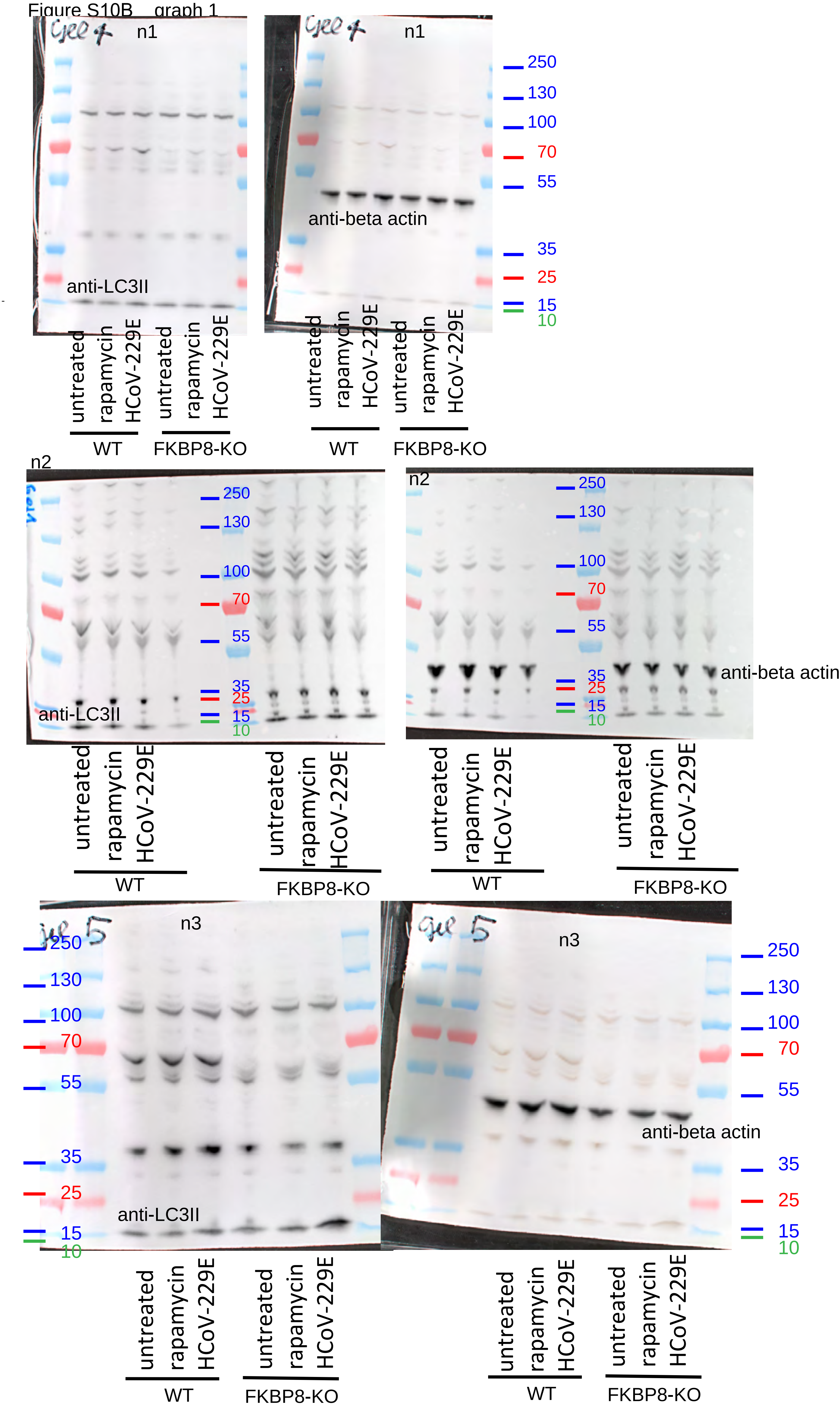

Figure S10B graph2

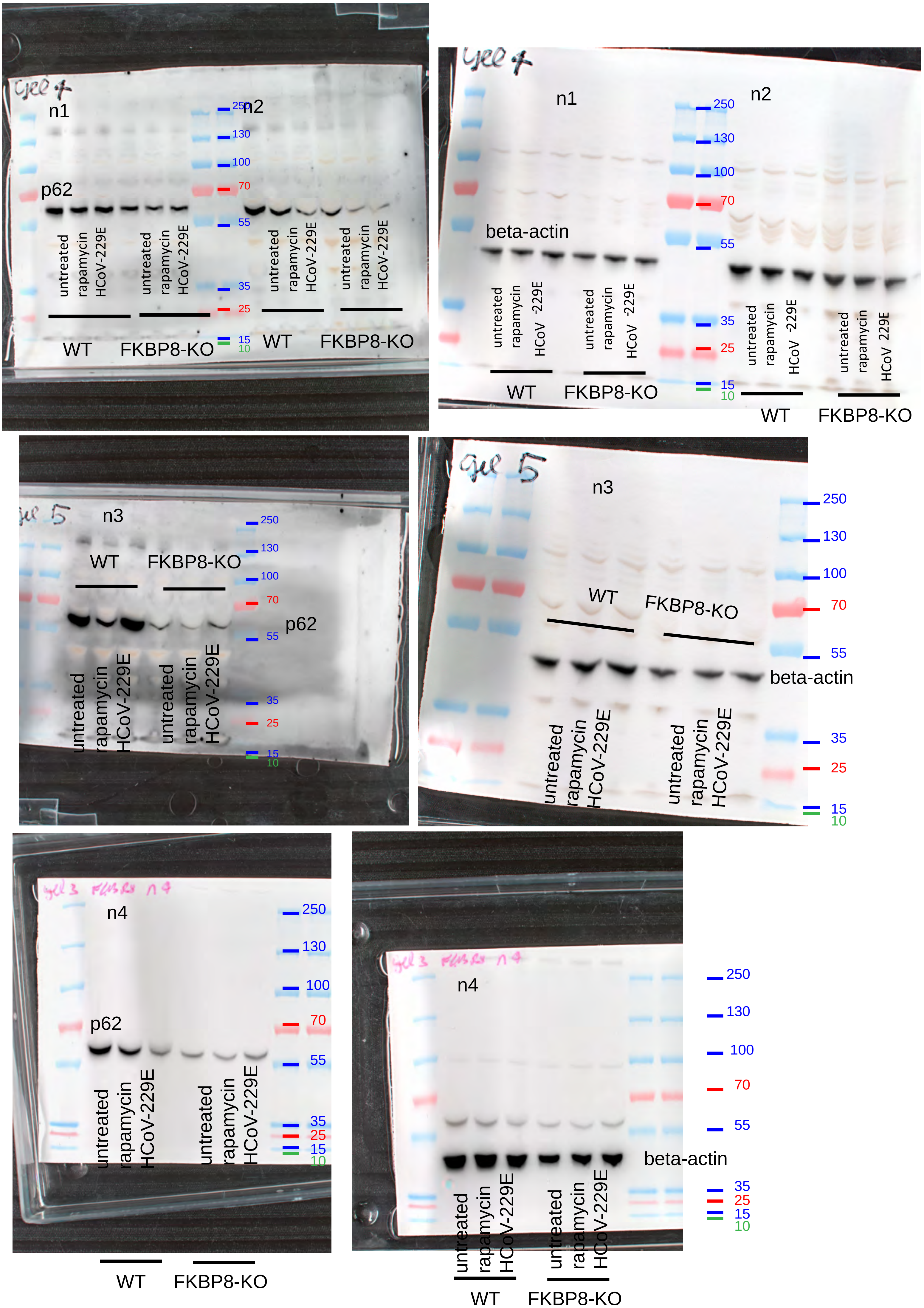

Figure S10C Rapamycin treatment

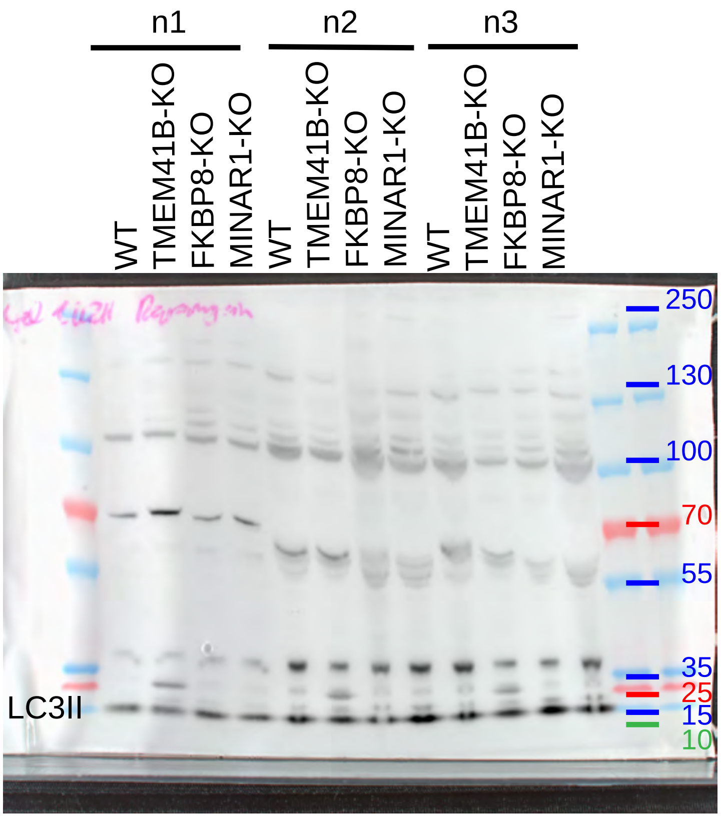

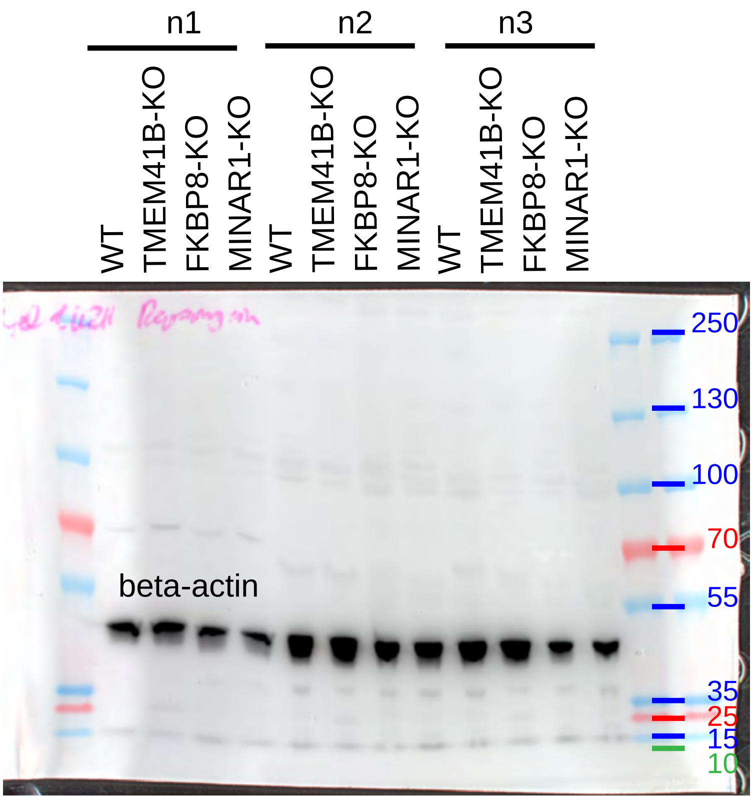

Figure S4C

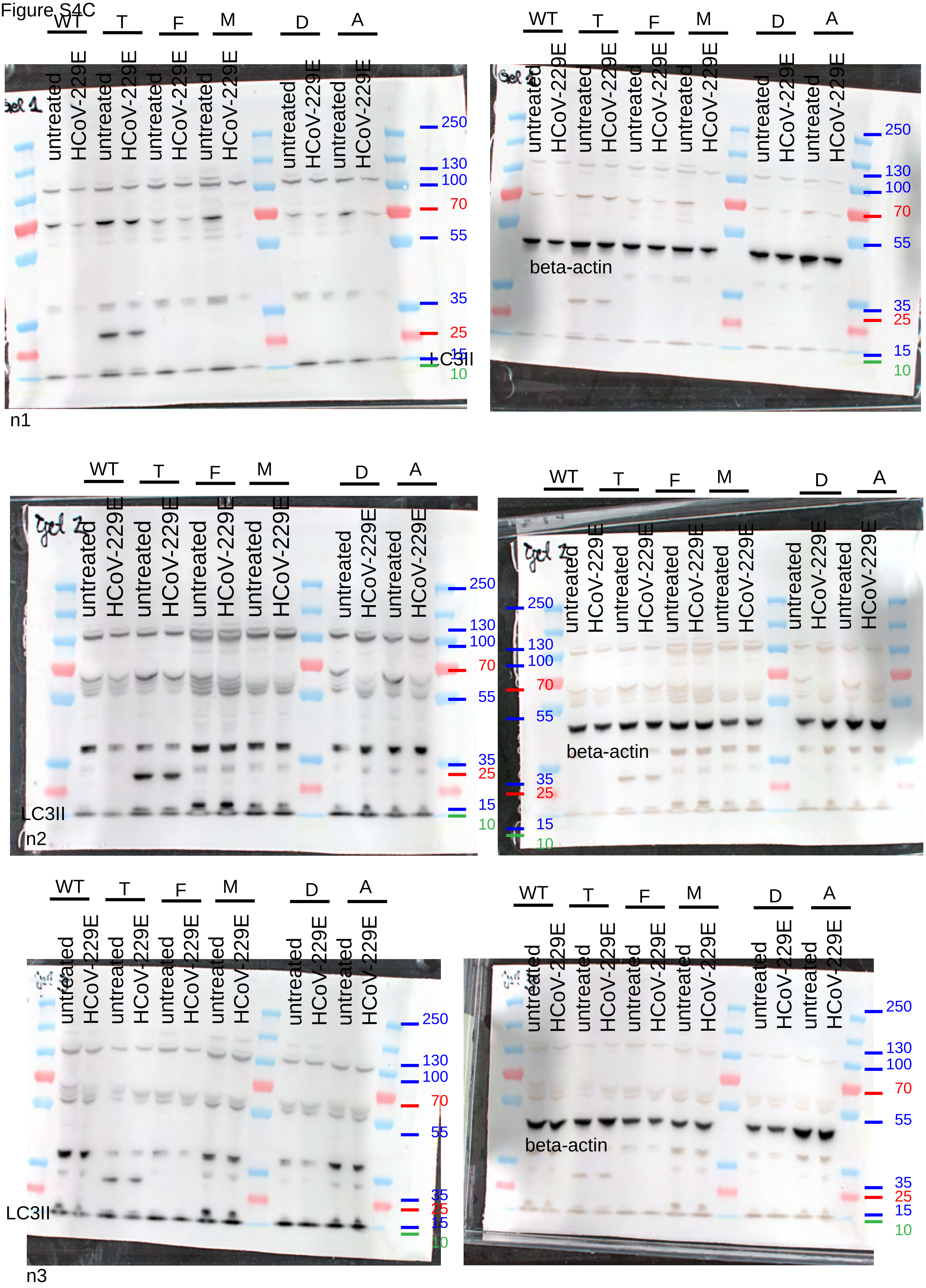

WT, T=TMEM41B-KO, F=FKBP8-KO, M=MINAR1-KO, D=DDP4-KO, A=APN-KO
